# Suvorexant for Primary Insomnia: A Systematic Review and Meta-Analysis of Randomized Placebo-Controlled Trials

**DOI:** 10.1371/journal.pone.0136910

**Published:** 2015-08-28

**Authors:** Taro Kishi, Shinji Matsunaga, Nakao Iwata

**Affiliations:** Department of Psychiatry, Fujita Health University School of Medicine, Toyoake, Aichi, Japan; University of Florence, ITALY

## Abstract

**Objective:**

We performed a systematic review and meta-analysis of double-blind, randomized, placebo-controlled trials evaluating suvorexant for primary insomnia.

**Methods:**

Relevant studies were identified through searches of PubMed, databases of the Cochrane Library, and PsycINFO citations through June 27, 2015. We performed a systematic review and meta-analysis of suvorexant trial efficacy and safety outcomes. The primary efficacy outcomes were either subjective total sleep time (sTST) or subjective time-to-sleep onset (sTSO) at 1 month. The secondary outcomes were other efficacy outcomes, discontinuation rate, and individual adverse events. The risk ratio, number-needed-to-treat/harm, and weighted mean difference (WMD) and 95% confidence intervals (CI) based on a random effects model were calculated.

**Results:**

The computerized literature database search initially yielded 48 results, from which 37 articles were excluded following a review of titles and abstracts and another eight review articles after full-text review. Thus, we identified 4 trials that included a total of 3,076 patients. Suvorexant was superior to placebo with regard to the two primary efficacy outcomes (sTST: WMD = −20.16, 95% CI = −25.01 to −15.30, 1889 patients, 3 trials, sTSO: WMD = −7.62, 95% CI = −11.03 to −4.21, 1889 patients, 3 trials) and was not different from placebo in trial discontinuations. Suvorexant caused a higher incidence than placebo of at least one side effects, abnormal dreams, somnolence, excessive daytime sleepiness/sedation, fatigue, dry mouth, and rebound insomnia.

**Conclusions:**

Our analysis of published trial results suggests that suvorexant is effective in treating primary insomnia and is well-tolerated.

## Introduction

Primary insomnia is defined as sleeplessness that is not attributable to a medical, psychiatric, or environmental cause (Diagnostic and Statistical Manual of Mental Disorders, Fourth Edition, Text Revision: DSM-IV-TR). The prevalence of primary insomnia is reported to be approximately 1.6% in the general population in Finland [1]. Primary insomnia can lead to psychiatric disorders, such as major depressive disorder [2]. Medications used in the treatment of insomnia include nonbenzodiazepine receptor agonists, benzodiazepine receptor agonists, the selective melatonin receptor agonist ramelteon, and sedating antidepressants [3, 4]. However, these medications have associated risks of adverse events. Ramelteon is associated with somnolence [3]. Benzodiazepines are associated with events indicative of abuse potential [5] and motor vehicle accidents/violations [6], as well as rebound insomnia on withdrawal [5].

Suvorexant, a reversible dual orexin receptor antagonist, was approved in 2014 for marketing by the U.S. Food & Drug Administration (FDA) for insomnia. Orexins are neuropeptides secreted from the lateral hypothalamus neurons that are involved in regulating the sleep–wake cycle and play a role in keeping people awake [7, 8]. Two orexin neuropeptides, orexin-A (OXA) and orexin-B (OXB), have been identified, which act with different affinities through binding to 2 G-protein coupled receptors, OX1R and OX2R. Suvorexant binds reversibly to both receptors and inhibits the activation of the arousal system, thus, facilitating sleep induction and maintenance [8]. This mechanism represents a potential favorable characteristic of suvorexant over benzodiazepines, since benzodiazepines act through benzodiazepine receptors that are associated with a risk for physical dependence with chronic use [8]. To the best of our knowledge, there are four studies of suvorexant use for the treatment of patients with primary insomnia, conducted as phase 2 [9] and 3 trials [10, 11]. However, although the results of a systematic review and meta-analyses are considered to present a higher level of evidence than those from individual trials [12], there is no systematic review and meta-analysis of suvorexant with regard to the efficacy, tolerability, and safety in patients with primary insomnia. A meta-analysis can increase the statistical power for group comparisons and can overcome the limitation of sample size in underpowered studies [12]. To synthesize the available trial evidence, we carried out a systematic review and a meta-analysis of suvorexant in patients with primary insomnia to identify the characteristics of suvorexant by assessing the efficacy, discontinuation rate, and side effects of suvorexant versus placebo in the treatment of patients with primary insomnia.

## Methods

This systematic review and meta-analysis was performed according to the Preferred Reporting Items for Systematic Reviews and Meta-Analyses (PRISMA) guidelines [13] (**S1 PRISMA Checklist**).

### Inclusion Criteria, Search Strategy, Data Extraction, and Outcomes

We selected double-blind, randomized, placebo-controlled trials (RCTs) evaluating suvorexant treatment for patients with primary insomnia. Relevant studies were identified through searches of PubMed, databases of the Cochrane Library, and PsycINFO citations through June 27, 2015. The English key words “suvorexant” and “insomnia” were searched without language restriction. In addition, we evaluated information in the Japanese drug package insert for suvorexant and assessed data from phase 2 and phase 3 trials of suvorexant in *ClinicalTrials.gov* (https://clinicaltrials.gov/). When the data required for the meta-analysis were missing, the corresponding authors and/or pharmaceutical company were contacted for additional information. Two authors (T.K. and S.M.) independently extracted, checked, and entered the data into the Review Manager software (Version 5.3 for Windows, Cochrane Collaboration, http://tech.cochrane.org/Revman).

### Data Synthesis and Statistical Analysis

The data synthesis is presented in **S1 Table**. The primary outcomes for measuring efficacy were either subjective total sleep time (sTST) or subjective time-to-sleep onset (sTSO) at month 1. The secondary outcomes were as follows: sTST and sTSO at week 1 and month 3; subjective wake after sleep onset (sWASO), subjective quality of sleep (sQUAL), and subjective number of awakenings (sNAW) at week 1, month 1, and month 3; latency to persistent sleep (LPS) and wake after sleep onset (WASO) at day 1, month 1, and month 3; subjective refreshed feeling on waking (sFRESH) and Insomnia Severity Index (ISI) score [14] at month 1 and month 3; clinician global impression of severity (CGI-S) [15], patient global impression of severity (PGI-S) [15], clinician global impression of improvement (CGI-I) [15], and patient global impression of improvement (PGI-I) [15] at week 2, month 1, and month 3; response rate at month 3 (responders: ISI ≥6-point improvement from the baseline) [11]; and discontinuation rate. Moreover, we analyzed reported adverse events that occurred with high incidence (≥5%) or that were related to sleep and psychiatric symptoms despite low incidence (<5%), including cataplexy, excessive daytime sleepiness/sedation, sleep paralysis, complex sleep-related behaviors, hypnagogic hallucination, hypnopompic hallucination, abnormal dream, suicidal ideation, and events suggesting drug-abuse potential. In addition, we evaluated rebound insomnia (the proportion of patients in each treatment group with worsening beyond their baseline sTST and sTSO was calculated for each of the first 3 nights of run-out as well as on any of the 3 nights) and withdrawal symptoms (the proportion of patients with newly emergent or worsening of 3 or more symptoms on the Tyrer Withdrawal Symptom Questionnaire [16] was calculated for each of the first 3 nights of run-out as well as across all 3 nights) following suvorexant removal in the run-out phase. Based on the studies included in the systematic review and meta-analysis [11], the discontinuation rate and withdrawal symptoms (Tyrer Withdrawal Symptom Questionnaire) were compared between the switching suvorexant to suvorexant group and the switching suvorexant to placebo group; and rebound insomnia was compared between the switching suvorexant to placebo group and the switching placebo to placebo group.

We based our analyses on intention-to-treat (ITT) or modified ITT (i.e., at least one dose or at least one follow-up assessment) data. However, we included only data before cross-over (first phase) from the cross-over study [9] to increase the sample size as much as possible. The systematic review and meta-analysis was performed using Review Manager. To combine the studies, we used the conservative random effects model described by DerSimonian and Laird [17] to account for the possibility that the underlying effects differed across studies and populations that are usually heterogeneous. For continuous data, weighted mean difference (WMD) was calculated. For dichotomous data, the risk ratio (RR) was estimated along with its 95% confidence interval (CI). When there were several suvorexant treatment groups with different suvorexant doses, we used the combined data from all suvorexant treatment doses for dichotomous data, as recommended by the Cochrane Collaboration. [12] However, although we did not combine continuous data from different suvorexant treatment dose groups (e.g., 10 mg/day, 20 mg/day, 40 mg/day or 80 mg/day); since the FDA approved suvorexant at a maximum dose of 20 mg/day (elderly patients: 15 mg/day), we selected data from the suvorexant 20 mg/day (elderly patients: 15 mg/day) treatment group. In this study, when the random effects model showed significant between-group differences, the number-needed-to-treat/harm (NNT/NNH) was calculated. Following this, the NNT/NNH values were derived from the risk differences (RDs) using the formula NNT/NNH = 1/RD. We assessed the methodological qualities of the articles included in the systematic review and meta-analysis on the basis of the Cochrane risk of bias criteria (Cochrane Collaboration, http://www.cochrane.org/). Statistical heterogeneity was assessed with Cochran’s Q statistic test and the I^2^ statistics. However, significant heterogeneity was not detected in the primary outcomes (sTST or sTSO at month 1); thus, we did not perform a sensitivity analysis. Although we combined primary outcome data from 2 suvorexant 20 mg (approved dose) studies with that from one suvorexant 40 mg (not approved dose) study, because the number of studies examining low and high doses of suvorexant is too few to support a separate meta-analysis, we did not perform a subgroup analysis based on suvorexant dosing (i.e., low dose versus high dose) to investigate potential dose effects on efficacy and safety.

## Results

### Study Characteristics

**Fig 1:**
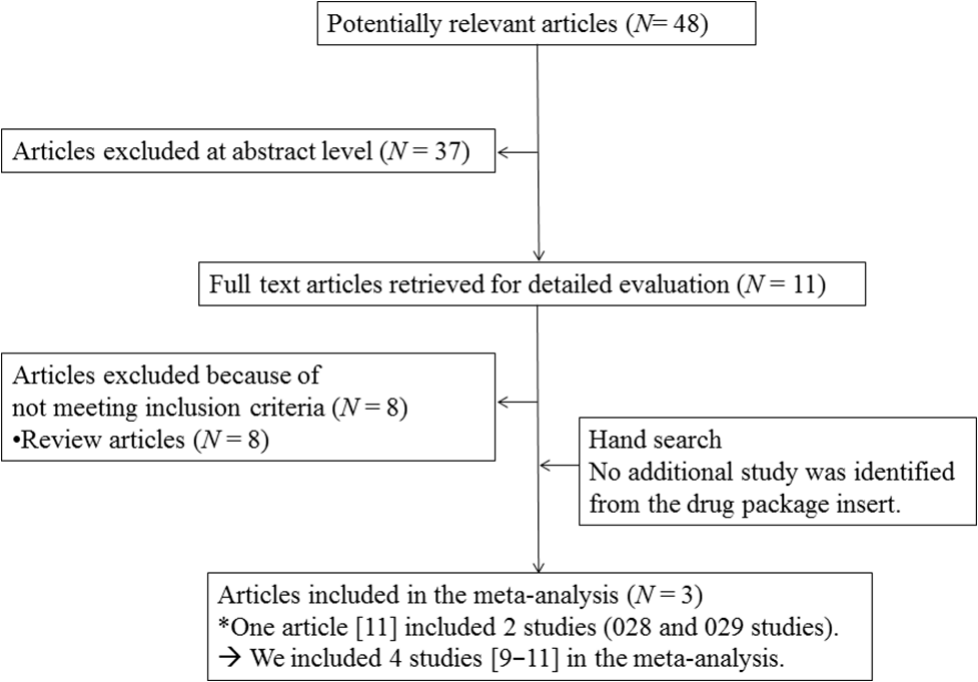
Preferred Reporting Items for Systematic reviews and Meta-Analysis (PRISMA) flow diagram.

The initial computerized search yielded 48 results (**Fig 1**). We excluded 37 articles following a review of the titles and abstracts and 8 review articles after full-text review. One article [11] included 2 studies (028 and 029 studies). We identified 4 studies [9–11] that included 3,076 patients: 3 studies were double-blind RCTs that mentioned the required study design details, while the fourth study [9] was a double-blind cross-over RCT. All the studies were published in English and were conducted in multiple countries. The study duration was 1 month in one study [9], 3 months in two studies [11], and 1 year study in one study [10]. All studies were industry sponsored. Primary insomnia in all studies was diagnosed according to DSM-IV-TR. The mean patient age was 56.6 years, 38.2% were male, and 77.0% were white. Three of the studies had a run-in period to exclude placebo responders and used polysomnography, sleep diary, and questionnaire for evaluating efficacy outcomes [9, 11] (**S1 and S2 Tables**). The remaining study [10] used a sleep diary and questionnaire for evaluating efficacy outcomes (**S1 and S2 Tables**). All studies were of high methodological quality according to the Cochrane Risk of Bias Criteria, since they were all double-blind RCTs that mentioned the required study design details as follows: all studies had adequate sequence generation and adequate concealed allocation. Participants or assessors in all studies were judged to be adequately blinded. All studies disclosed the involvement of industry sponsorship and used ITT or modified ITT data. However, one study [9] was a double-blind, cross-over RCT. This study did not report data before cross-over in all outcomes other than LPS and discontinuation rate. The 028 and 029 studies used the same protocol, which enhances the chances of yielding similar results [11]. Other characteristics of the studies are summarized in **S2 Table**.

### Efficacy Outcomes

**Fig 2:**
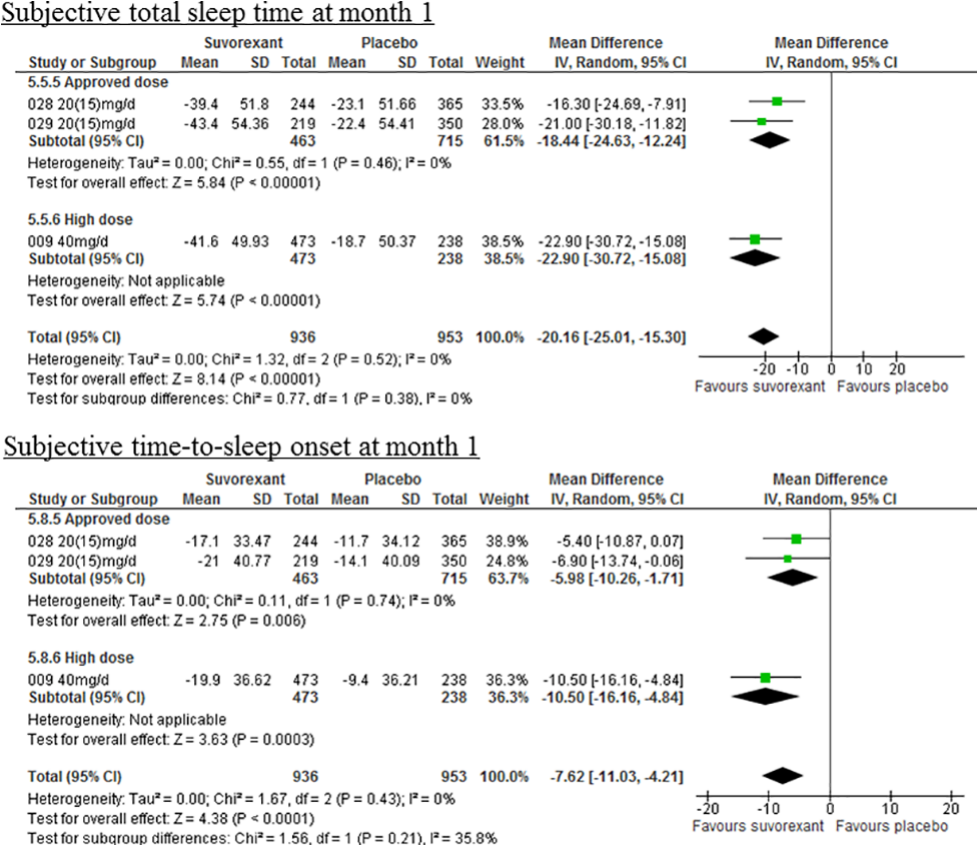
Forest plots of study primary outcomes.

Suvorexant was superior to placebo with regard to not only all primary efficacy outcomes (**Fig 2**), but also all secondary efficacy outcomes with the exceptions of sQUAL at week 1, sWASO and LPS at month 3, and sNAW at any time-point (**Tables 1 and 2, and S1 Fig**).

**Table 1:** Efficacy outcome (diary measures) results.

Diary Measures	N	n	WMD	95% CI	P(Z)^a^	I^2^	P(Q)^b^
sTST at Week 1	3	1979	−19.06	−27.06, −11.06	<0.00001	73	0.02
sTST at Month 1	3	1889	−20.16	−25.01, −15.30	<0.00001	0	0.52
sTST at Month 3	2	1089	−15.97	−27.22, −4.73	0.005	63	0.10
sTSO at Week 1	3	1979	−7.69	−10.72, −4.66	<0.00001	5	0.35
sTSO at Month 1	3	1889	−7.62	−11.03, −4.21	<0.0001	0	0.43
sTSO at Month 3	2	1089	−5.96	−10.01, −1.91	0.004	0	0.59
sWASO at Week 1	2	1219	−5.68	−9.18, −2.18	0.001	0	0.49
sWASO at Month 1	3	1915	−7.75	−10.87, −4.62	<0.00001	0	0.62
sWASO at Month 3	2	1089	−4.82	−9.99. 0.36	0.07	34	0.22
sQUAL at Week 1	2	1219	−0.05	−0.15, 0.05	0.33	0	0.33
sQUAL at Month 1	3	1915	−0.17	−0.25, −0.09	<0.0001	0	0.52
sQUAL at Month 3	2	1089	−0.10	−0.20, −0.00	0.05	0	1.00
sFRESH at Month 1	3	1915	−0.17	−0.25, −0.09	<0.0001	0	0.53
sFRESH at Month 3	2	1089	−0.15	−0.25, −0.05	0.003	0	0.33
sNAW at Week 1	2	1219	0.05	−0.05. 0.15	0.33	0	0.33
sNAW at Month 1	3	1915	0.03	−0.05, 0.11	0.42	0	0.53
sNAW at Month 3	2	1089	0.00	−0.10, 0.10	1.00	0	1.00

^a^P(Z): The significance of the pooled effect size was determined with Z test.

^b^P(Q): Cochrane’s Q statistic test used to assess the heterogeneity.

Abbreviations: 95% CI, 95% confidence interval; N, number of comparisons; n, number of patients; sFRESH, subjective refreshed feeling on waking (0–4 scale); sNAW, subjective number of awakenings; sQUAL, subjective quality of sleep (1–4 scale); sTSO, subjective time to sleep onset (minutes); sTST, subjective total sleep time (minutes); sWASO, subjective wake after sleep onset (minutes); WMD, weighted mean difference.

**Table 2:** Analysis of efficacy outcomes from rating scales and polysomnography.

**Rating Scale**	**N**	**n**	**WMD**	**95% CI**	**P(Z)** ^**a**^	**I** ^**2**^	**P(Q)** ^**b**^
ISI at Month 1	3	1899	−1.35	−1.78, −0.93	<0.00001	0	0.64
ISI at Month 3	2	1084	−1.18	−1.78, −0.59	<0.0001	0	0.75
CGI-S at Week 2	2	1154	−0.35	−0.45, −0.25	<0.00001	0	0.33
CGI-S at Month 1	3	1898	−0.37	−0.45, −0.29	<0.00001	0	0.53
CGI-S at Month 3	2	1084	−0.30	−0.40, −0.20	<0.00001	0	1.00
PGI-S at Week 2	2	1154	−0.35	−0.45, −0.25	<0.00001	0	0.32
PGI-S at Month 1	3	1898	−0.34	−0.42, −0.27	<0.00001	0	0.44
PGI-S at Month 3	2	1084	−0.30	−0.42, −0.18	<0.00001	0	1.00
CGI-I at Week 2	2	1154	−0.33	−0.45, −0.21	<0.00001	0	0.46
CGI-I at Month 1	3	1898	−0.36	−0.45, −0.27	<0.00001	0	0.57
CGI-I at Month 3	2	1084	−0.45	−0.61, −0.29	<0.00001	0	0.54
PGI-I at Week 2	2	1154	−0.37	−0.49, −0.25	<0.00001	0	0.46
PGI-I at Month 1	3	1898	−0.47	−0.59, −0.36	<0.00001	13	0.32
PGI-I at Month 3	2	1084	−0.33	−0.45, −0.21	<0.00001	0	0.46
**Polysomnography**	**N**	**n**	**WMD**	**95% CI**	**P(Z)**	**I** ^**2**^	**P(Q)**
LPS at Day 1	3	1071	−10.83	−15.13, −6.52	<0.00001	0	0.63
LPS at Month 1	3	1008	−10.82	−16.72, −4.93	0.0003	35	0.22
LPS at Month 3	2	805	−4.69	−12.27, 2.89	0.23	58	0.12
WASO at Day 1	2	906	−34.35	−39.59, −29.10	<0.00001	0	0.41
WASO at Month 1	2	859	−25.32	−31.25, −19.39	<0.00001	0	0.72
WASO at Month 3	2	802	−23.78	−38.08, −9.47	0.001	82	0.02
**Response rate**	**N**	**n**	**RR**	**95% CI**	**P(Z)**	**I** ^**2**^	**P(Q)**
Month 3*	2	1705	0.78	0.71–0.85	<0.00001	0	0.48

^a^P(Z): The significance of the pooled effect size was determined with Z test.

^b^P(Q): Cochrane’s Q statistic test used to assess the heterogeneity.

*Number need to treat = 8; 95% CI = 6–13; P(Z)<0.00001; I^2^ = 0; P(Q) = 0.73.

Abbreviations: 95% CI, 95% confidence interval; CGI-I, Clinical Global Impression-Improvement scale (1–7 scale); CGI-S, Clinical Global Impression-Severity scale (1–7 scale); ISI, Insomnia Severity Index (0–28 scale); LPS, latency to onset of persistent sleep (minutes); PGI-I, Patient Global Impression-Improvement scale (1–7 scale); PGI-S, Patient Global Impression-Severity scale (0–5 scale); N, number of comparisons; n, number of patients; RR, risk ratio; WASO, wakefulness after persistent sleep onset (minutes); WMD, weighted mean difference.

#### Discontinuation Rate and Individual Adverse Events

Suvorexant did not differ from the placebo with regard to discontinuation due to all-cause, inefficacy, and intolerability (**Table 3 and S2 Fig**). Except for the lower incidence of back pain in the suvorexant group than in the placebo group, at least one side effects, abnormal dreams, somnolence, excessive daytime sleepiness/sedation, fatigue, and dry mouth was more frequent in the suvorexant group than in the placebo group (**Table 3 and S2 Fig**). Suvorexant did not differ from placebo with regard to other individual adverse events (**Table 3 and S2 Fig**).

**Table 3:** Treatment discontinuation and individual adverse events.

	N	n	RR	95% CI	P(Z)^a^	I^2^	P(Q)^b^	NNT	95% CI	P(Z)^a^	I^2^	P(Q)^b^
Discontinuation due to all cause	4	3076	0.96	0.82–1.11	0.56	0	0.73					
Discontinuation due to intolerability	4	3076	0.94	0.60–1.47	0.77	44	0.15					
Discontinuation due to inefficacy	4	3076	0.76	0.53–1.10	0.14	0	0.73					
At least one adverse events	3	2809	1.06	0.99–1.14	0.09	0	0.73					
At least one side effects	3	2809	1.58	1.35–1.85	<0.00001	0	0.54	10	7–20	<0.00001	45	0.16
Serious adverse event	3	2809	0.49	0.14–1.66	0.25	71	0.03					
Somnolence	3	2809	3.16	2.18–4.57	<0.00001	0	0.39	14	10–25	<0.00001	65	0.06
Excessive daytime sleepiness/sedation	3	2809	3.34	1.08–10.32	0.04	0	0.85	100	100–∞	0.03	22	0.28
Fatigue	3	2809	2.09	1.08–4.06	0.03	35	0.21	50	25–∞	0.04	62	0.07
Cataplexy	3	2809	Not estimable									
Sleep paralysis	3	2809	2.74	0.47–16.0	0.26	0	0.93					
Complex sleep–related behaviors	3	2809	1.65	0.17–15.86	0.66	0	0.93					
Hypnagogic hallucination	3	2809	2.31	0.38–13.95	0.36	0	0.94					
Hypnopompic hallucination	3	2809	1.65	0.17–15.86	0.66	0	0.93					
Abnormal dreams	2	2030	2.87	1.10–7.52	0.03	0	0.67	100	50–∞	0.009	0	0.76
Suicidal ideation	3	2809	1.72	0.24–12.13	0.59	20	0.28					
Events suggesting drug–abuse potential	3	2809	1.05	0.67–1.65	0.84	0	0.87					
Fall	3	2809	0.84	0.44–1.62	0.60	0	0.42					
Headache	3	2809	1.13	0.85–1.51	0.38	0	0.66					
Dizziness	3	2809	0.87	0.57–1.31	0.50	0	0.77					
Back pain	3	2809	0.52	0.28–0.98	0.04	0	0.70	Not significant				
Dry mouth	3	2809	1.99	1.10–3.61	0.02	0	0.54	Not significant				
Nasopharyngitis	3	2809	0.95	0.71–1.28	0.74	0	0.89					
Motor vehicle accidents/violations	3	2809	1.16	0.52–2.60	0.72	14	0.31					

^a^P(Z): The significance of the pooled effect size was determined using the Z test.

^b^P(Q): Cochrane’s Q statistic test was used to assess data heterogeneity.

Abbreviations: 95% CI, 95% confidence interval; N, number of comparisons; n, number of patients; NNH, number need to harm; RR, risk ratio; ∞, infinitude.

#### Withdrawal Symptoms in Run-Out Phase

The switching suvorexant to placebo group showed more rebound insomnia (sTST) at day 1, day 3, and days 1–3 than the switching placebo to placebo group (**Table 4 and S3 Fig**). Suvorexant did not differ from placebo with regard to withdrawal symptoms and rebound insomnia (sTSO) at day 1, day 2, day 3, and days 1–3 (**Table 4 and S3 Fig**). There were no significant differences in the discontinuation rate between the suvorexant and placebo treatment groups (**Table 4 and S3 Fig**).

**Table 4:** The results regarding the outcome related to run-out phase.

**The switching suvorexant to suvorexant group versus The switching suvorexant to placebo group**												
	**N**	**n**	**RR**	**95% CI**	**P(Z)** ^**a**^	**I** ^**2**^	**P(Q)** ^**b**^	**NNH**	**95% CI**	**P(Z)** ^**a**^	**I** ^**2**^	**P(Q)** ^**b**^
Discontinuation due to all cause	3	1406	0.84	0.33–2.12	0.72	0	0.95					
Discontinuation due to intolerability	3	1406	0.22	0.03–1.95	0.18	0	0.71					
Discontinuation due to inefficacy	3	1406	0.35	0.01–8.64	0.52	na						
Withdrawal (TWSQ) at Day 1	3	1132	0.67	0.33–1.38	0.28	0	0.71					
Withdrawal (TWSQ) at Day 2	3	1125	0.83	0.47–1.45	0.51	0	0.43					
Withdrawal (TWSQ) at Day 3	3	1101	1.01	0.51–1.97	0.99	0	0.87					
Withdrawal (TWSQ) at Day 1–3	3	1207	0.98	0.64–1.51	0.94	0	1.00					
**The switching suvorexant to placebo group versus The switching placebo to placebo group**												
	**N**	**n**	**RR**	**95% CI**	**P(Z)** ^**a**^	**I** ^**2**^	**P(Q)** ^**b**^	**NNH**	**95% CI**	**P(Z)** ^**a**^	**I** ^**2**^	**P(Q)** ^**b**^
Rebound insomnia (sTST) at Day 1	3	1398	1.20	1.02–1.41	0.03	0	0.99	20	10–100	0.03	0	0.99
Rebound insomnia (sTST) at Day 2	3	1399	1.21	1.00–1.46	0.05	2	0.36					
Rebound insomnia (sTST) at Day 3	3	1387	1.26	1.05–1.51	0.01	0	0.41	17	9–100	0.01	0	0.45
Rebound insomnia (sTST) at Day 1–3	3	1486	1.24	1.10–1.40	0.0005	0	0.43	11	7–25	0.0004	0	0.44
Rebound insomnia (sTSO) at Day 1	3	1398	1.09	0.91–1.32	0.35	0	0.39					
Rebound insomnia (sTSO) at Day 2	3	1399	1.05	0.85–1.30	0.64	0	0.54					
Rebound insomnia (sTSO) at Day 3	3	1387	1.06	0.85–1.33	0.60	25	0.26					
Rebound insomnia (sTSO) at Day 1–3	3	1486	1.09	0.94–1.26	0.24	12	0.32					

^a^P(Z): The significance of the pooled effect size was determined with Z test.

^b^P(Q): Cochrane’s Q statistic test used to assess the heterogeneity.

Abbreviations: 95% CI, 95% confidence interval; N, number of comparisons; n, number of patients; NNH, number need to harm; RR, risk ratio; TWSQ, Tyrer Withdrawal Symptom Questionnaire; sTSO, subjective time to sleep onset; sTST, subjective total sleep time.

## Discussion

This is the first comprehensive, systematic review and meta-analysis of suvorexant compared with placebo for the treatment of patients with primary insomnia. Suvorexant was superior to placebo with regard to the two primary efficacy outcomes (sTST: WMD = −20.16, 95% CI = −25.01 to −15.30, sTSO: WMD = −7.62, 95% CI = −11.03 to −4.21). Suvorexant was also superior to placebo with regard to other subjective and objective efficacy outcomes, with the exceptions of sQUAL at week 1, sWASO and LPS at month 3, and sNAW at all time-points. Effect size of all efficacy outcomes except for CGI-I seemed to become subtly smaller over time. A recent meta-analysis of ramelteon [3] showed reduced subject sleep latency and improved sleep quality and LPS, with a similar effect size as suvorexant. However, ramelteon was not superior to placebo with regard to sTST, sWASO, sNAW, and WASO [3]. Ramelteon was also associated with a higher incidence of somnolence than placebo, but had a similar RR as was reported for suvorexant [3]. Another meta-analysis [4] showed that benzodiazepines were superior to placebo with regard to sWASO, WASO, and sleep quality with a similar effect size to that of suvorexant, while they did not differ from placebo with regard to sTST. This meta-analysis [4] also reported that antidepressants were superior to placebo with regard to sTST, WASO, and sleep quality, again with a similar effect size to that of suvorexant. Given these data, the efficacy of suvorexant appears to be similar to that of ramelteon, benzodiazepine, and antidepressants. Moreover, similar to other psychotropic drugs, suvorexant caused higher incidences of at least one side effects, from among somnolence, excessive daytime sleepiness/sedation, fatigue, and dry mouth compared to placebo. The results of our meta-analysis shows that suvorexant did not induce the risk of suicide ideation and/or behavior, events indicative of abuse potential, or motor vehicle accidents/violations compared with placebo. Benzodiazepines were reported to be associated with events indicative of abuse potential, withdrawal symptoms, rebound insomnia [5], and motor vehicle accidents/violations [6]. In addition, although suvorexant caused more rebound insomnia (sTST) than placebo, suvorexant did not differ from placebo in any measured outcomes related to withdrawal symptoms and rebound insomnia (sTSO). Although the meta-analyses for benzodiazepines and antidepressants did not include safety outcomes, such as individual side effects, and we did not directly compare the results of our meta-analysis with those from previous meta-analyses because we did not perform a multiple treatment meta-analysis of these interventions, suvorexant still seems to have efficacy similar to other psychotropic drugs and to be well-tolerated.

High-dose suvorexant seemed to cause a higher incidence of side effects than low-dose suvorexant [9–11]. Although suvorexant is primarily metabolized through cytochrome P450, mainly by CYP3A4 with considerably less contribution by CYP2C19, it also has the potential to inhibit CYP3A4 and intestinal P-glycoprotein [18]. Because the suvorexant blood level and risk of side effects will be higher with concomitant use of CYP3A4 inhibitors such as grapefruit juice, azole antifungals, macrolide antibiotics, and fluvoxamine, concomitant drugs must be used carefully to avoid potential drug-drug interactions.

Suvorexant caused a higher incidence of abnormal dreams than placebo despite a small effect size (NNH = 100). In a mouse study [19], suvorexant increased total rapid eye movement (REM) sleep time, which is consistent with the results reported for patients with primary insomnia [9]. Increased REM may trigger a higher incidence of abnormal dreaming. However, suvorexant did not differ from placebo with regard to the risk of drug-induced narcolepsy (complex sleep-related behaviors, hallucinations, sleep paralysis, and cataplexy) in the meta-analysis. Several investigations have reported a role for orexin in psychiatric disorders such as mood disorders, anxiety disorders, and schizophrenia, and have suggested that orexin receptor antagonists, including suvorexant, may have therapeutic effects against these psychiatric disorders [20–22]. Patients with psychiatric disorders frequently suffer from various degrees of insomnia [23]. However, no study has examined the efficacy and safety of suvorexant for the treatment of psychiatric patients with insomnia, and such a study is warranted in the future.

A previous systematic review has described the efficacy and safety of suvorexant for the treatment of primary insomnia [24]. However, the methodology of our study was different in that we performed a meta-analysis assessing the efficacy, discontinuation rate, and side effects of suvorexant versus placebo following PRISMA reporting guidelines. Moreover, there were differences in inclusion criteria, data extraction and quality assessment between our study and the past study.

There are several limitations to the present analysis. First, the number of studies included in this meta-analysis is small. Although we combined data on primary outcomes from 2 suvorexant 20 mg (approved dose) studies [11] with data from one suvorexant 40 mg (not approved dose) study [10], because number of studies examined was low and the number of patients treated with high dose suvorexant was too small to allow a separate meta-analysis, we did not perform a subgroup analysis for suvorexant dosing (i.e., approved dose versus not approved dose) to investigate dose effects on efficacy and safety. However, when this high dose study was excluded from the primary outcomes, suvorexant was superior to placebo in sTST and sTSO at 1 month, although the effect size did not notably change (**Fig 2**). Second, because 3 out of the 4 studies evaluated [9, 11] had short trial durations, we could not determine whether suvorexant will have long-term effects on insomnia. Third, because a Funnel plot is generally used only if 10 or more studies are included in the meta-analysis, we did not utilize this plot for exploring potential publication bias.

## Conclusions

Suvorexant exhibited good efficacy in the treatment of primary insomnia and was well-tolerated. Moreover, suvorexant did not show any change in risk of suicide ideation and/or behavior or events indicative of abuse potential. However, the small number of studies included in this meta-analysis necessitates a longitudinal suvorexant study to be performed with a large patient sample to strengthen our findings.

## Supporting Information

S1 PRISMA Checklist (DOC)

S1 Fig Forest plots of efficacy secondary outcomes. (PDF)

S2 Fig Forest plots of safety secondary outcomes. (PDF)

S3 Fig Forest plots of study run-out phase outcomes. (PDF)

S1 Table Data synthesis. (PDF)

S2 Table Study, patient, and treatment characteristics for the analyzed randomized, controlled trials. (PDF)
