# Supplementary material for: Suvorexant for Primary Insomnia: A Systematic Review and Meta-Analysis of Randomized Placebo-Controlled Trials
Source: PLoS One. 2015 Aug 28;10(8):e0136910. doi: 10.1371/journal.pone.0136910 (PMC4552781; doi:10.1371/journal.pone.0136910)

S1 Fig. Forest plots of efficacy secondary outcomes.

S1-1 Fig. Subjective total sleep time (minutes) at Week 1

Study or Subgroup	Suvorexant Mean	Suvorexant SD	Suvorexant Total	Placebo Mean	Placebo SD	Placebo Total	Weight	Mean Difference IV, Random, 95% CI
**4.4.5 Approved dose**								
028 20(15)mg/d	-28.2	41.78	248	-14.6	41.55	376	34.7%	-13.60 [-20.28, -6.92]
029 20(15)mg/d	-30.8	46.53	231	-14	45.75	364	32.3%	-16.80 [-24.42, -9.18]
**Subtotal (95% CI)**			**479**			**740**	**67.0%**	**-14.99 [-20.02, -9.97]**
Heterogeneity: Tau² = 0.00; Chi² = 0.38, df = 1 (P = 0.54); I² = 0%								
Test for overall effect: Z = 5.85 (P < 0.00001)								
**4.4.6 High dose**								
009 40mg/d	-41.1	48.3	508	-14.1	48.6	252	33.0%	-27.00 [-34.32, -19.68]
**Subtotal (95% CI)**			**508**			**252**	**33.0%**	**-27.00 [-34.32, -19.68]**
Heterogeneity: Not applicable								
Test for overall effect: Z = 7.23 (P < 0.00001)								
**Total (95% CI)**			**987**			**992**	**100.0%**	**-19.06 [-27.06, -11.06]**
Heterogeneity: Tau² = 36.46; Chi² = 7.40, df = 2 (P = 0.02); I² = 73%								
Test for overall effect: Z = 4.67 (P < 0.00001)								
Test for subgroup differences: Chi² = 7.02, df = 1 (P = 0.008), I² = 85.8%								

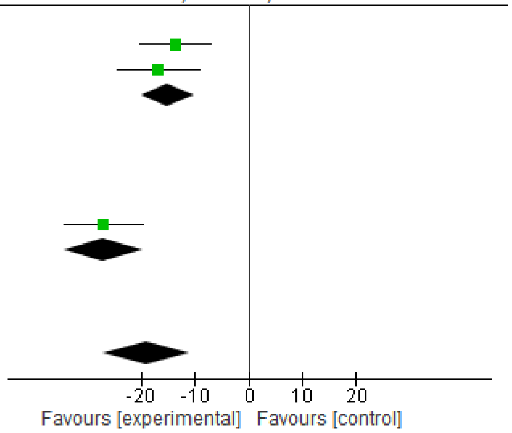

S1-2 Fig. Subjective total sleep time (minutes) at Month 1

Study or Subgroup	Suvorexant Mean	Suvorexant SD	Suvorexant Total	Placebo Mean	Placebo SD	Placebo Total	Weight	Mean Difference IV, Random, 95% CI
**4.5.5 Approved dose**								
028 20(15)mg/d	-39.4	51.8	244	-23.1	51.66	365	33.5%	-16.30 [-24.69, -7.91]
029 20(15)mg/d	-43.4	54.36	219	-22.4	54.41	350	28.0%	-21.00 [-30.18, -11.82]
**Subtotal (95% CI)**			**463**			**715**	**61.5%**	**-18.44 [-24.63, -12.24]**
Heterogeneity: Tau² = 0.00; Chi² = 0.55, df = 1 (P = 0.46); I² = 0%								
Test for overall effect: Z = 5.84 (P < 0.00001)								
**4.5.6 High dose**								
009 40mg/d	-41.6	49.93	473	-18.7	50.37	238	38.5%	-22.90 [-30.72, -15.08]
**Subtotal (95% CI)**			**473**			**238**	**38.5%**	**-22.90 [-30.72, -15.08]**
Heterogeneity: Not applicable								
Test for overall effect: Z = 5.74 (P < 0.00001)								
**Total (95% CI)**			**936**			**953**	**100.0%**	**-20.16 [-25.01, -15.30]**
Heterogeneity: Tau² = 0.00; Chi² = 1.32, df = 2 (P = 0.52); I² = 0%								
Test for overall effect: Z = 8.14 (P < 0.00001)								
Test for subgroup differences: Chi² = 0.77, df = 1 (P = 0.38), I² = 0%								

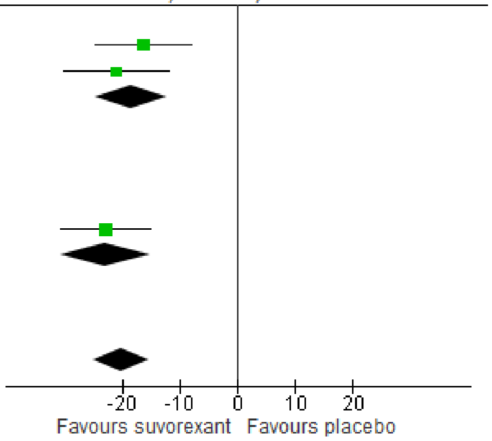

S1-3 Fig. Subjective total sleep time (minutes) at Month 3

Study or Subgroup	Suvorexant Mean	Suvorexant SD	Suvorexant Total	Placebo Mean	Placebo SD	Placebo Total	Weight	Mean Difference IV, Random, 95% CI
**4.6.5 Approved dose**								
028 20(15)mg/d	-51.2	53.16	228	-40.6	51.67	339	53.3%	-10.60 [-19.42, -1.78]
029 20(15)mg/d	-59.8	59.44	197	-37.7	59.79	325	46.7%	-22.10 [-32.64, -11.56]
**Subtotal (95% CI)**			**425**			**664**	**100.0%**	**-15.97 [-27.22, -4.73]**
Heterogeneity: Tau² = 41.52; Chi² = 2.69, df = 1 (P = 0.10); I² = 63%								
Test for overall effect: Z = 2.78 (P = 0.005)								
**Total (95% CI)**			**425**			**664**	**100.0%**	**-15.97 [-27.22, -4.73]**
Heterogeneity: Tau² = 41.52; Chi² = 2.69, df = 1 (P = 0.10); I² = 63%								
Test for overall effect: Z = 2.78 (P = 0.005)								
Test for subgroup differences: Not applicable								

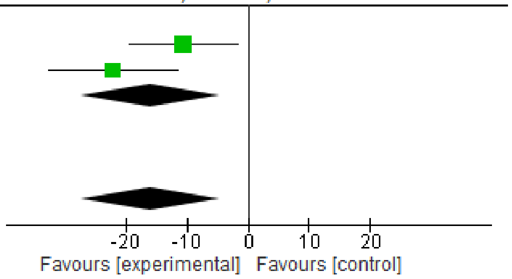

S1-4 Fig. Subjective time to sleep onset (minutes) at Week 1

Study or Subgroup	Suvorexant Mean	Suvorexant SD	Suvorexant Total	Placebo Mean	Placebo SD	Placebo Total	Weight	Mean Difference IV, Random, 95% CI
**4.7.5 Approved dose**								
028 20(15)mg/d	-15.2	28.12	248	-9.6	28.69	376	41.4%	-5.60 [-10.15, -1.05]
029 20(15)mg/d	-14.2	32.57	231	-6.7	33.1	364	29.9%	-7.50 [-12.90, -2.10]
**Subtotal (95% CI)**			**479**			**740**	**71.3%**	**-6.39 [-9.87, -2.91]**
Heterogeneity: Tau² = 0.00; Chi² = 0.28, df = 1 (P = 0.60); I² = 0%								
Test for overall effect: Z = 3.60 (P = 0.0003)								
**4.7.6 High dose**								
009 40mg/d	-17.7	36.8	508	-6.8	36.45	252	28.7%	-10.90 [-16.42, -5.38]
**Subtotal (95% CI)**			**508**			**252**	**28.7%**	**-10.90 [-16.42, -5.38]**
Heterogeneity: Not applicable								
Test for overall effect: Z = 3.87 (P = 0.0001)								
**Total (95% CI)**			**987**			**992**	**100.0%**	**-7.69 [-10.72, -4.66]**
Heterogeneity: Tau² = 0.40; Chi² = 2.11, df = 2 (P = 0.35); I² = 5%								
Test for overall effect: Z = 4.97 (P < 0.00001)								
Test for subgroup differences: Chi² = 1.84, df = 1 (P = 0.18), I² = 45.6%								

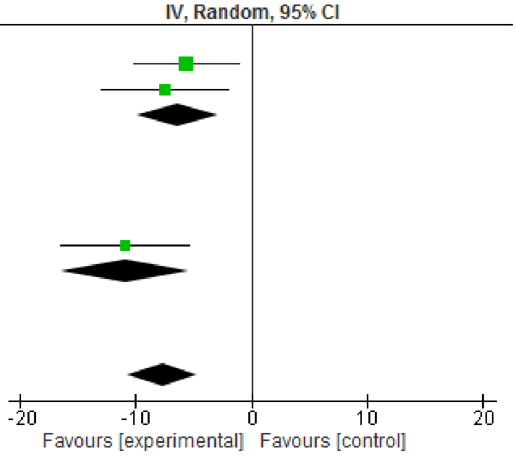

S1-5 Fig. Subjective time to sleep onset (minutes) at Month 1

Study or Subgroup	Suvorexant Mean	Suvorexant SD	Suvorexant Total	Placebo Mean	Placebo SD	Placebo Total	Weight	Mean Difference IV, Random, 95% CI
**4.8.5 Approved dose**								
028 20(15)mg/d	-17.1	33.47	244	-11.7	34.12	365	38.9%	-5.40 [-10.87, 0.07]
029 20(15)mg/d	-21	40.77	219	-14.1	40.09	350	24.8%	-6.90 [-13.74, -0.06]
**Subtotal (95% CI)**			**463**			**715**	**63.7%**	**-5.98 [-10.26, -1.71]**
Heterogeneity: Tau² = 0.00; Chi² = 0.11, df = 1 (P = 0.74); I² = 0%								
Test for overall effect: Z = 2.75 (P = 0.006)								
**4.8.6 High dose**								
009 40mg/d	-19.9	36.62	473	-9.4	36.21	238	36.3%	-10.50 [-16.16, -4.84]
**Subtotal (95% CI)**			**473**			**238**	**36.3%**	**-10.50 [-16.16, -4.84]**
Heterogeneity: Not applicable								
Test for overall effect: Z = 3.63 (P = 0.0003)								
**Total (95% CI)**			**936**			**953**	**100.0%**	**-7.62 [-11.03, -4.21]**
Heterogeneity: Tau² = 0.00; Chi² = 1.67, df = 2 (P = 0.43); I² = 0%								
Test for overall effect: Z = 4.38 (P < 0.0001)								
Test for subgroup differences: Chi² = 1.56, df = 1 (P = 0.21), I² = 35.8%								

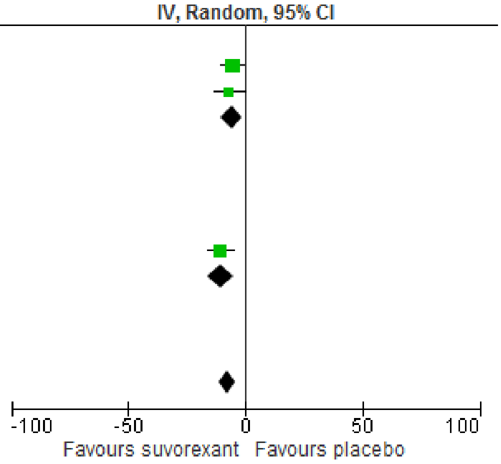

S1-6 Fig. Subjective time to sleep onset (minutes) at Month 3

Study or Subgroup	Suvorexant Mean	Suvorexant SD	Suvorexant Total	Placebo Mean	Placebo SD	Placebo Total	Weight	Mean Difference IV, Random, 95% CI
**4.9.5 Approved dose**								
028 20(15)mg/d	-22.5	29.27	228	-17.3	29.12	339	68.3%	-5.20 [-10.10, -0.30]
029 20(15)mg/d	-28.1	40.82	197	-20.5	40.47	325	31.7%	-7.60 [-14.80, -0.40]
**Subtotal (95% CI)**			**425**			**664**	**100.0%**	**-5.96 [-10.01, -1.91]**
Heterogeneity: Tau² = 0.00; Chi² = 0.29, df = 1 (P = 0.59); I² = 0%								
Test for overall effect: Z = 2.88 (P = 0.004)								
**Total (95% CI)**			**425**			**664**	**100.0%**	**-5.96 [-10.01, -1.91]**
Heterogeneity: Tau² = 0.00; Chi² = 0.29, df = 1 (P = 0.59); I² = 0%								
Test for overall effect: Z = 2.88 (P = 0.004)								
Test for subgroup differences: Not applicable								

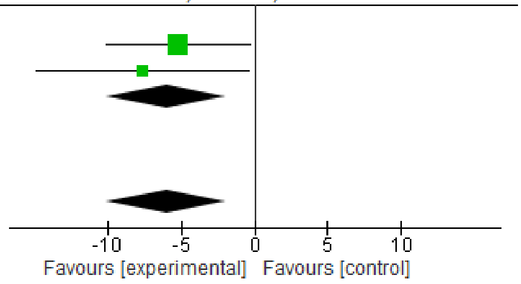

S1-7 Fig. Subjective wake after sleep onset (minutes) at Week 1

Study or Subgroup	Suvorexant Mean	Suvorexant SD	Suvorexant Total	Placebo Mean	Placebo SD	Placebo Total	Weight	Mean Difference IV, Random, 95% CI
**4.41.5 Approved dose**								
028 20(15)mg/d	-17.3	28.12	248	-10.6	28.69	376	59.3%	-6.70 [-11.25, -2.15]
029 20(15)mg/d	-17.8	33.34	231	-13.6	33.1	364	40.7%	-4.20 [-9.68, 1.28]
**Subtotal (95% CI)**			**479**			**740**	**100.0%**	**-5.68 [-9.18, -2.18]**
Heterogeneity: Tau² = 0.00; Chi² = 0.47, df = 1 (P = 0.49); I² = 0%								
Test for overall effect: Z = 3.18 (P = 0.001)								
**Total (95% CI)**			**479**			**740**	**100.0%**	**-5.68 [-9.18, -2.18]**
Heterogeneity: Tau² = 0.00; Chi² = 0.47, df = 1 (P = 0.49); I² = 0%								
Test for overall effect: Z = 3.18 (P = 0.001)								
Test for subgroup differences: Not applicable								

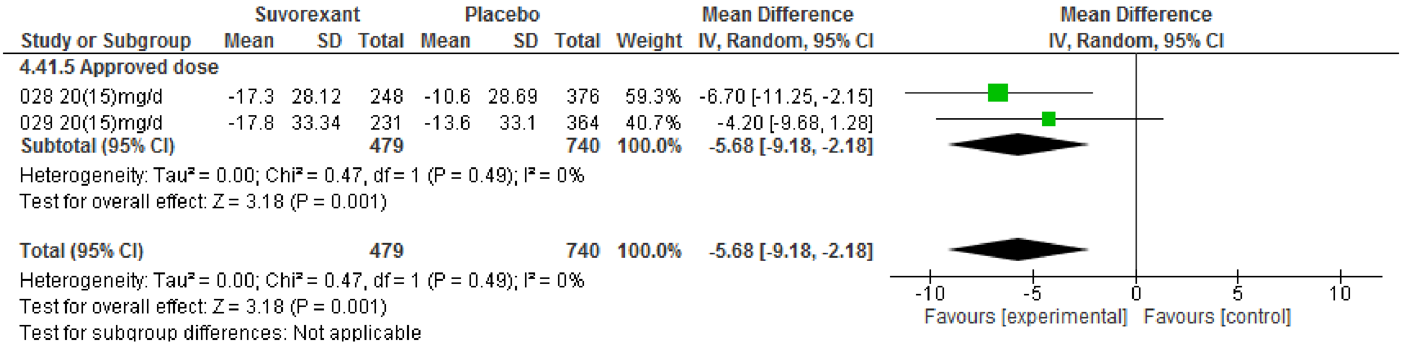

S1-8 Fig. Subjective wake after sleep onset (minutes) at Month 1

Study or Subgroup	Suvorexant Mean	Suvorexant SD	Suvorexant Total	Placebo Mean	Placebo SD	Placebo Total	Weight	Mean Difference IV, Random, 95% CI
**4.42.5 Approved dose**								
028 20(15)mg/d	-23.3	35.07	244	-17.9	35.09	365	30.3%	-5.40 [-11.09, 0.29]
029 20(15)mg/d	-29.2	35.49	219	-20.8	35.32	350	27.3%	-8.40 [-14.38, -2.42]
**Subtotal (95% CI)**			**463**			**715**	**57.6%**	**-6.82 [-10.94, -2.70]**
Heterogeneity: Tau² = 0.00; Chi² = 0.51, df = 1 (P = 0.48); I² = 0%								
Test for overall effect: Z = 3.25 (P = 0.001)								
**4.42.6 High dose**								
009 40mg/d	-23.5	31.69	492	-14.5	31.15	245	42.4%	-9.00 [-13.80, -4.20]
**Subtotal (95% CI)**			**492**			**245**	**42.4%**	**-9.00 [-13.80, -4.20]**
Heterogeneity: Not applicable								
Test for overall effect: Z = 3.67 (P = 0.0002)								
**Total (95% CI)**			**955**			**960**	**100.0%**	**-7.75 [-10.87, -4.62]**
Heterogeneity: Tau² = 0.00; Chi² = 0.96, df = 2 (P = 0.62); I² = 0%								
Test for overall effect: Z = 4.86 (P < 0.00001)								
Test for subgroup differences: Chi² = 0.45, df = 1 (P = 0.50), I² = 0%								

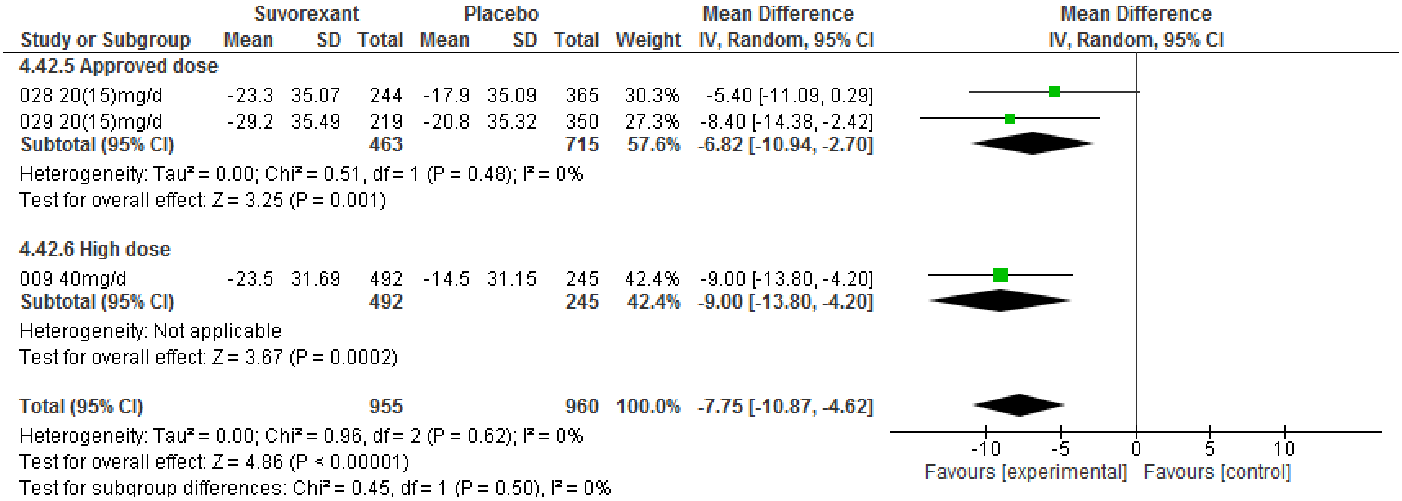

S1-9 Fig. Subjective wake after sleep onset (minutes) at Month 3

Study or Subgroup	Suvorexant Mean	Suvorexant SD	Suvorexant Total	Placebo Mean	Placebo SD	Placebo Total	Weight	Mean Difference IV, Random, 95% CI
**4.43.5 Approved dose**								
028 20(15)mg/d	-32	33.13	228	-29.6	32.88	339	54.4%	-2.40 [-7.94, 3.14]
029 20(15)mg/d	-37.5	35.81	197	-29.8	35.87	325	45.6%	-7.70 [-14.04, -1.36]
**Subtotal (95% CI)**			**425**			**664**	**100.0%**	**-4.82 [-9.99, 0.36]**
Heterogeneity: Tau² = 4.81; Chi² = 1.52, df = 1 (P = 0.22); I² = 34%								
Test for overall effect: Z = 1.82 (P = 0.07)								
**Total (95% CI)**			**425**			**664**	**100.0%**	**-4.82 [-9.99, 0.36]**
Heterogeneity: Tau² = 4.81; Chi² = 1.52, df = 1 (P = 0.22); I² = 34%								
Test for overall effect: Z = 1.82 (P = 0.07)								
Test for subgroup differences: Not applicable								

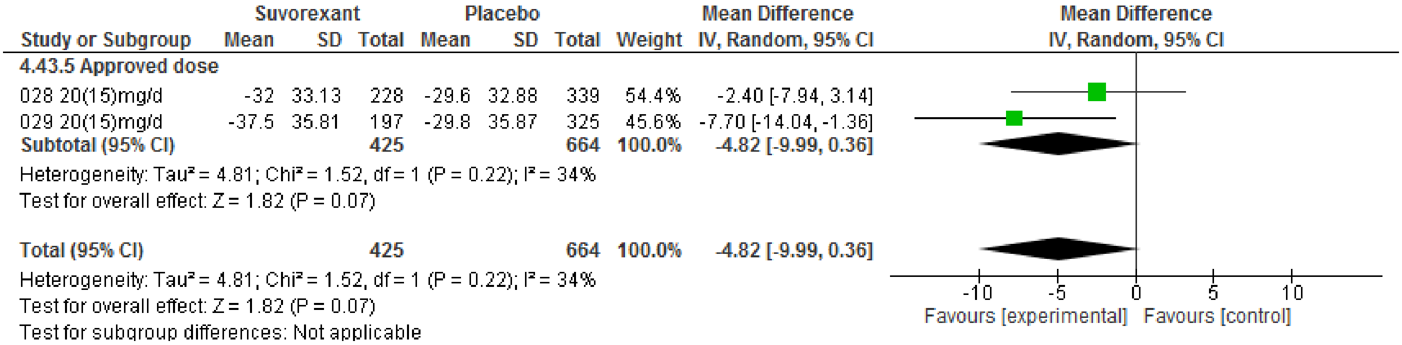

S1-10 Fig. Subjective quality of sleep (1–4 scale) at Week 1

Study or Subgroup	Suvorexant Mean	Suvorexant SD	Suvorexant Total	Placebo Mean	Placebo SD	Placebo Total	Weight	Mean Difference IV, Random, 95% CI
**4.18.5 Approved dose**								
028 20(15)mg/d	-0.2	0.803	248	-0.2	0.989	376	50.0%	0.00 [-0.14, 0.14]
029 20(15)mg/d	-0.2	0.775	231	-0.1	0.973	364	50.0%	-0.10 [-0.24, 0.04]
**Subtotal (95% CI)**			**479**			**740**	**100.0%**	**-0.05 [-0.15, 0.05]**
Heterogeneity: Tau² = 0.00; Chi² = 0.96, df = 1 (P = 0.33); I² = 0%								
Test for overall effect: Z = 0.98 (P = 0.33)								
**Total (95% CI)**			**479**			**740**	**100.0%**	**-0.05 [-0.15, 0.05]**
Heterogeneity: Tau² = 0.00; Chi² = 0.96, df = 1 (P = 0.33); I² = 0%								
Test for overall effect: Z = 0.98 (P = 0.33)								
Test for subgroup differences: Not applicable								

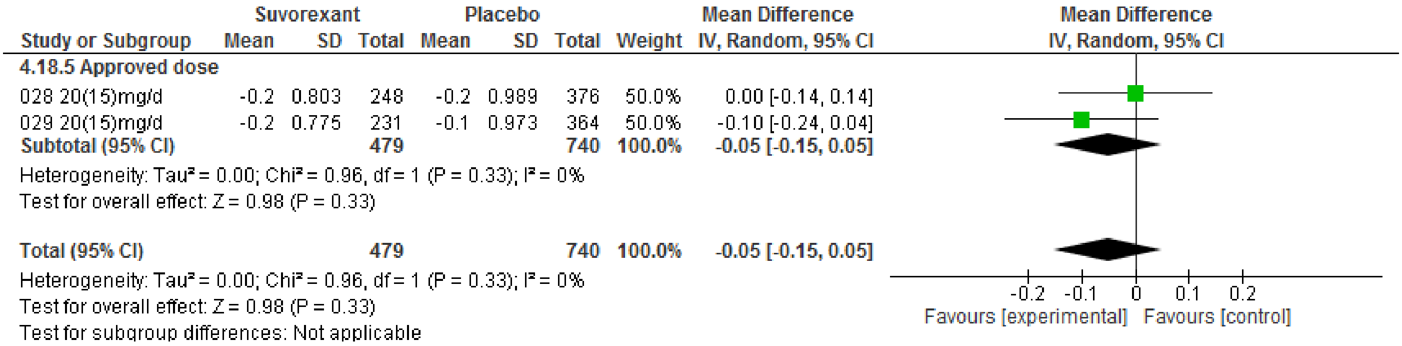

S1-11 Fig. Subjective quality of sleep (1–4 scale) at Month 1

Study or Subgroup	Suvorexant Mean	Suvorexant SD	Suvorexant Total	Placebo Mean	Placebo SD	Placebo Total	Weight	Mean Difference IV, Random, 95% CI
**4.19.5 Approved dose**								
028 20(15)mg/d	-0.3	0.797	244	-0.2	0.975	365	31.8%	-0.10 [-0.24, 0.04]
029 20(15)mg/d	-0.4	0.755	219	-0.2	0.955	350	31.8%	-0.20 [-0.34, -0.06]
**Subtotal (95% CI)**			**463**			**715**	**63.5%**	**-0.15 [-0.25, -0.05]**
Heterogeneity: Tau² = 0.00; Chi² = 0.96, df = 1 (P = 0.33); I² = 0%								
Test for overall effect: Z = 2.94 (P = 0.003)								
**4.19.6 High dose**								
009 40mg/d	-0.3	0.975	492	-0.1	0.799	245	36.5%	-0.20 [-0.33, -0.07]
**Subtotal (95% CI)**			**492**			**245**	**36.5%**	**-0.20 [-0.33, -0.07]**
Heterogeneity: Not applicable								
Test for overall effect: Z = 2.97 (P = 0.003)								
**Total (95% CI)**			**955**			**960**	**100.0%**	**-0.17 [-0.25, -0.09]**
Heterogeneity: Tau² = 0.00; Chi² = 1.31, df = 2 (P = 0.52); I² = 0%								
Test for overall effect: Z = 4.14 (P < 0.0001)								
Test for subgroup differences: Chi² = 0.35, df = 1 (P = 0.55), I² = 0%								

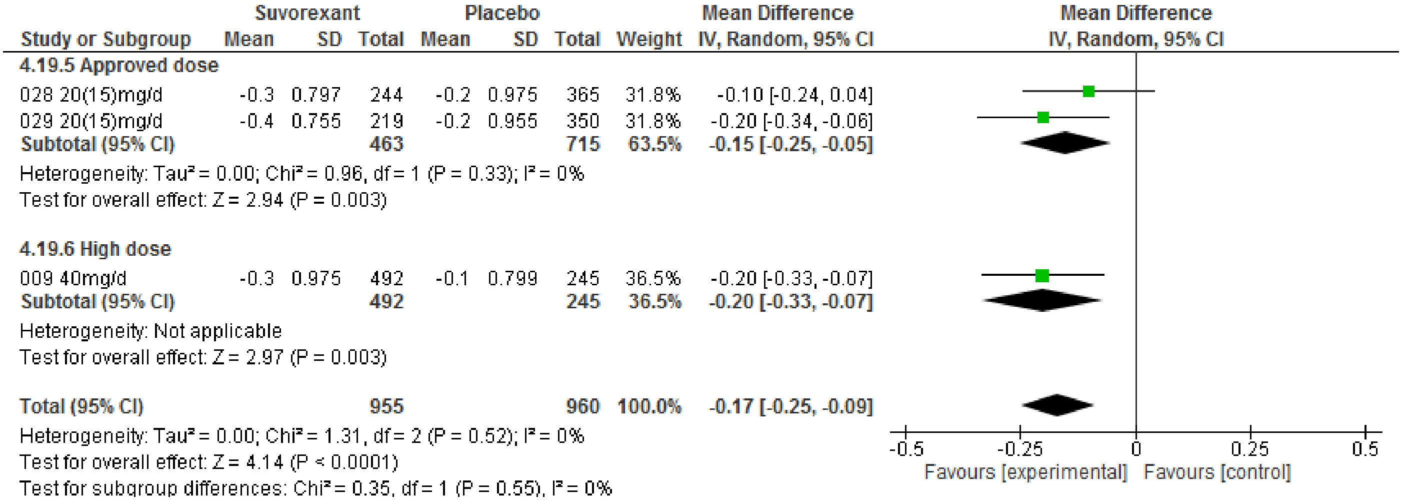

S1-12 Fig. Subjective quality of sleep (1–4 scale) at Month 3

Study or Subgroup	Suvorexant Mean	Suvorexant SD	Suvorexant Total	Placebo Mean	Placebo SD	Placebo Total	Weight	Mean Difference IV, Random, 95% CI
**4.20.5 Approved dose**								
028 20(15)mg/d	-0.4	0.77	228	-0.3	0.939	339	50.0%	-0.10 [-0.24, 0.04]
029 20(15)mg/d	-0.5	0.716	197	-0.4	0.92	325	50.0%	-0.10 [-0.24, 0.04]
**Subtotal (95% CI)**			**425**			**664**	**100.0%**	**-0.10 [-0.20, -0.00]**
Heterogeneity: Tau² = 0.00; Chi² = 0.00, df = 1 (P = 1.00); I² = 0%								
Test for overall effect: Z = 1.96 (P = 0.05)								
**Total (95% CI)**			**425**			**664**	**100.0%**	**-0.10 [-0.20, -0.00]**
Heterogeneity: Tau² = 0.00; Chi² = 0.00, df = 1 (P = 1.00); I² = 0%								
Test for overall effect: Z = 1.96 (P = 0.05)								
Test for subgroup differences: Not applicable								

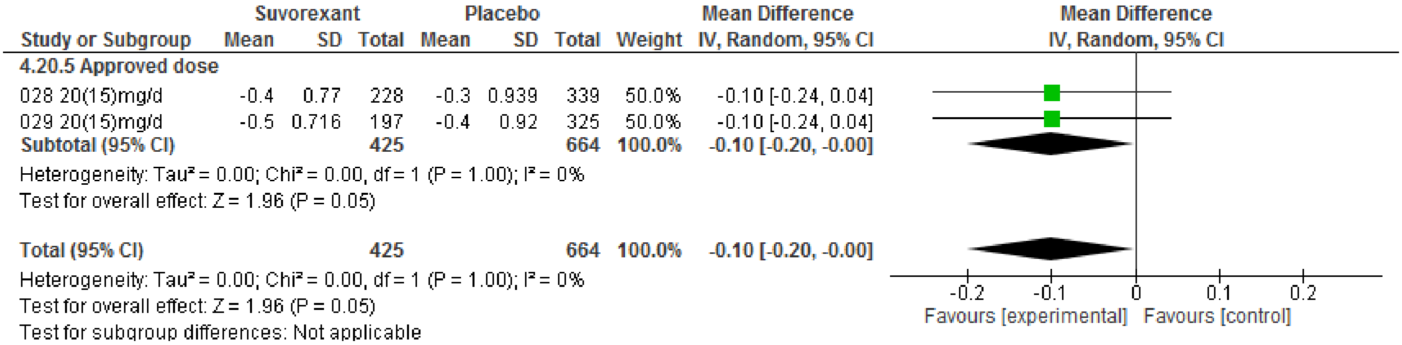

S1-13 Fig. Subjective refreshed feeling on waking (0–4 scale) at Month 1

Study or Subgroup	Suvorexant Mean	Suvorexant SD	Suvorexant Total	Placebo Mean	Placebo SD	Placebo Total	Weight	Mean Difference IV, Random, 95% CI
**4.21.5 Approved dose**								
028 20(15)mg/d	-0.4	0.797	244	-0.3	0.975	365	33.3%	-0.10 [-0.24, 0.04]
029 20(15)mg/d	-0.5	0.755	219	-0.3	0.955	350	33.3%	-0.20 [-0.34, -0.06]
**Subtotal (95% CI)**			**463**			**715**	**66.6%**	**-0.15 [-0.25, -0.05]**
Heterogeneity: Tau² = 0.00; Chi² = 0.96, df = 1 (P = 0.33); I² = 0%								
Test for overall effect: Z = 2.94 (P = 0.003)								
**4.21.6 High dose**								
009 40mg/d	-0.4	1.13	492	-0.2	0.799	245	33.4%	-0.20 [-0.34, -0.06]
**Subtotal (95% CI)**			**492**			**245**	**33.4%**	**-0.20 [-0.34, -0.06]**
Heterogeneity: Not applicable								
Test for overall effect: Z = 2.77 (P = 0.006)								
**Total (95% CI)**			**955**			**960**	**100.0%**	**-0.17 [-0.25, -0.09]**
Heterogeneity: Tau² = 0.00; Chi² = 1.28, df = 2 (P = 0.53); I² = 0%								
Test for overall effect: Z = 4.00 (P < 0.0001)								
Test for subgroup differences: Chi² = 0.32, df = 1 (P = 0.57), I² = 0%								

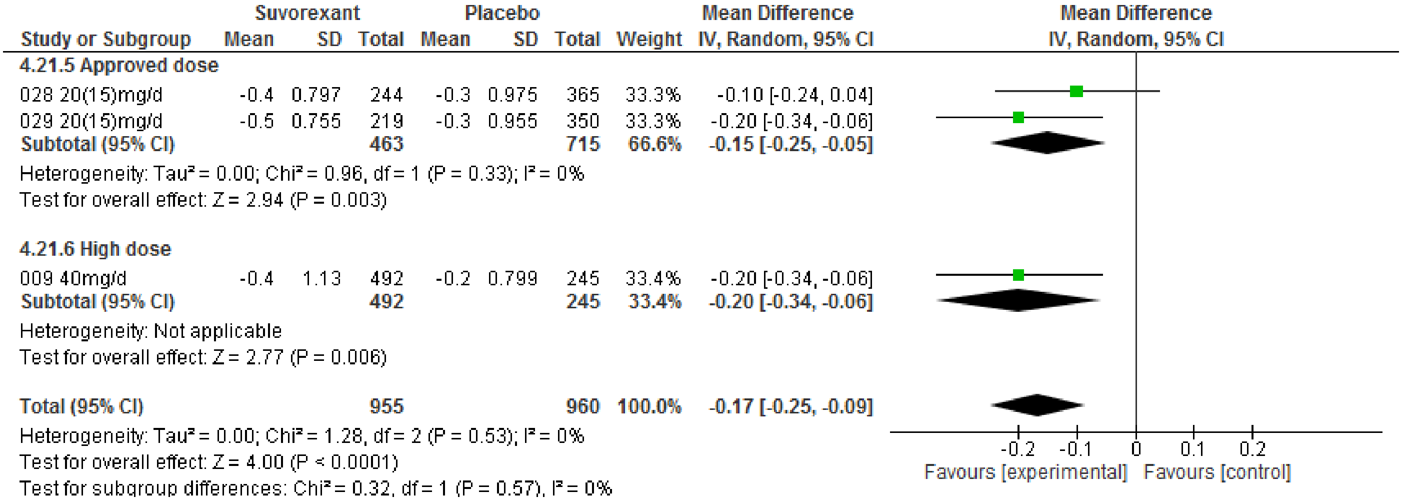

S1-14 Fig. Subjective refreshed feeling on waking (0–4 scale) at Month 3

Study or Subgroup	Suvorexant Mean	Suvorexant SD	Suvorexant Total	Placebo Mean	Placebo SD	Placebo Total	Weight	Mean Difference IV, Random, 95% CI
**4.22.5 Approved dose**								
028 20(15)mg/d	-0.6	0.77	228	-0.5	0.939	339	50.0%	-0.10 [-0.24, 0.04]
029 20(15)mg/d	-0.6	0.716	197	-0.4	0.92	325	50.0%	-0.20 [-0.34, -0.06]
**Subtotal (95% CI)**			**425**			**664**	**100.0%**	**-0.15 [-0.25, -0.05]**
Heterogeneity: Tau² = 0.00; Chi² = 0.96, df = 1 (P = 0.33); I² = 0%								
Test for overall effect: Z = 2.94 (P = 0.003)								
**Total (95% CI)**			**425**			**664**	**100.0%**	**-0.15 [-0.25, -0.05]**
Heterogeneity: Tau² = 0.00; Chi² = 0.96, df = 1 (P = 0.33); I² = 0%								
Test for overall effect: Z = 2.94 (P = 0.003)								
Test for subgroup differences: Not applicable								

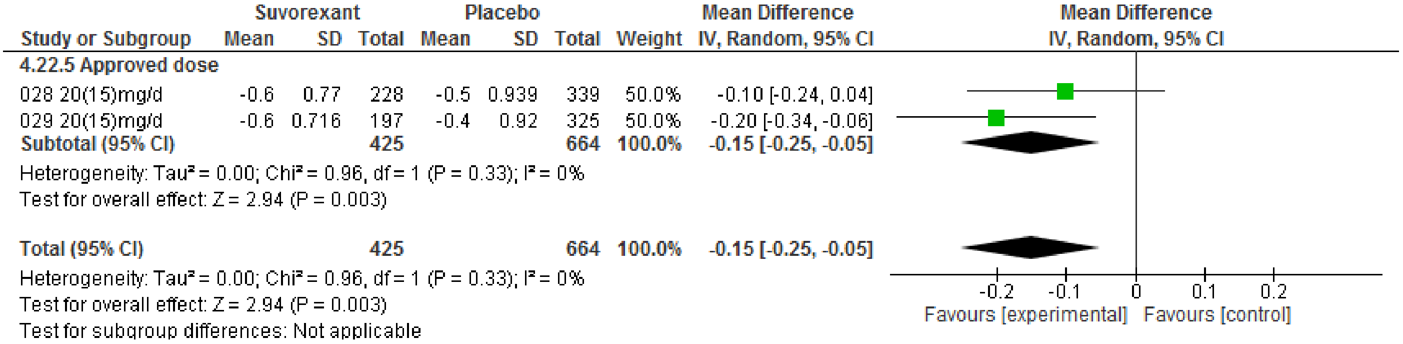

S1-15 Fig. Subjective number of awakenings at Week 1

Study or Subgroup	Suvorexant Mean	Suvorexant SD	Suvorexant Total	Placebo Mean	Placebo SD	Placebo Total	Weight	Mean Difference IV, Random, 95% CI
**4.23.5 Approved dose**								
028 20(15)mg/d	-0.2	0.803	248	-0.2	0.989	376	50.0%	0.00 [-0.14, 0.14]
029 20(15)mg/d	-0.1	0.775	231	-0.2	0.973	364	50.0%	0.10 [-0.04, 0.24]
**Subtotal (95% CI)**			**479**			**740**	**100.0%**	**0.05 [-0.05, 0.15]**
Heterogeneity: Tau² = 0.00; Chi² = 0.96, df = 1 (P = 0.33); I² = 0%								
Test for overall effect: Z = 0.98 (P = 0.33)								
**Total (95% CI)**			**479**			**740**	**100.0%**	**0.05 [-0.05, 0.15]**
Heterogeneity: Tau² = 0.00; Chi² = 0.96, df = 1 (P = 0.33); I² = 0%								
Test for overall effect: Z = 0.98 (P = 0.33)								
Test for subgroup differences: Not applicable								

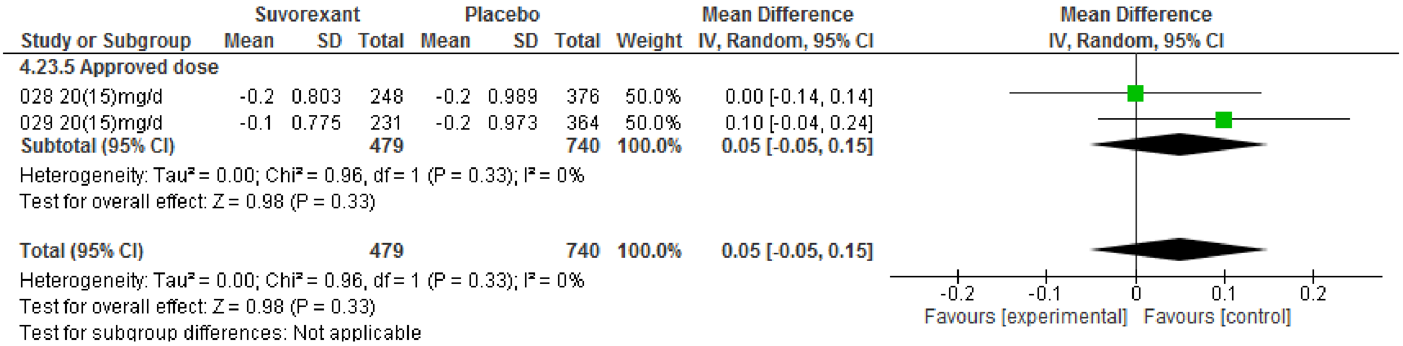

S1-16 Fig. Subjective number of awakenings at Month 1

Study or Subgroup	Suvorexant Mean	Suvorexant SD	Suvorexant Total	Placebo Mean	Placebo SD	Placebo Total	Weight	Mean Difference IV, Random, 95% CI
**4.24.5 Approved dose**								
028 20(15)mg/d	-0.3	0.797	244	-0.3	0.975	365	33.3%	0.00 [-0.14, 0.14]
029 20(15)mg/d	-0.3	0.755	219	-0.3	0.954	350	33.3%	0.00 [-0.14, 0.14]
**Subtotal (95% CI)**			**463**			**715**	**66.6%**	**0.00 [-0.10, 0.10]**
Heterogeneity: Tau² = 0.00; Chi² = 0.00, df = 1 (P = 1.00); I² = 0%								
Test for overall effect: Z = 0.00 (P = 1.00)								
**4.24.6 High dose**								
009 40mg/d	-0.2	1.13	492	-0.3	0.799	245	33.4%	0.10 [-0.04, 0.24]
**Subtotal (95% CI)**			**492**			**245**	**33.4%**	**0.10 [-0.04, 0.24]**
Heterogeneity: Not applicable								
Test for overall effect: Z = 1.39 (P = 0.17)								
**Total (95% CI)**			**955**			**960**	**100.0%**	**0.03 [-0.05, 0.11]**
Heterogeneity: Tau² = 0.00; Chi² = 1.28, df = 2 (P = 0.53); I² = 0%								
Test for overall effect: Z = 0.80 (P = 0.42)								
Test for subgroup differences: Chi² = 1.28, df = 1 (P = 0.26), I² = 22.0%								

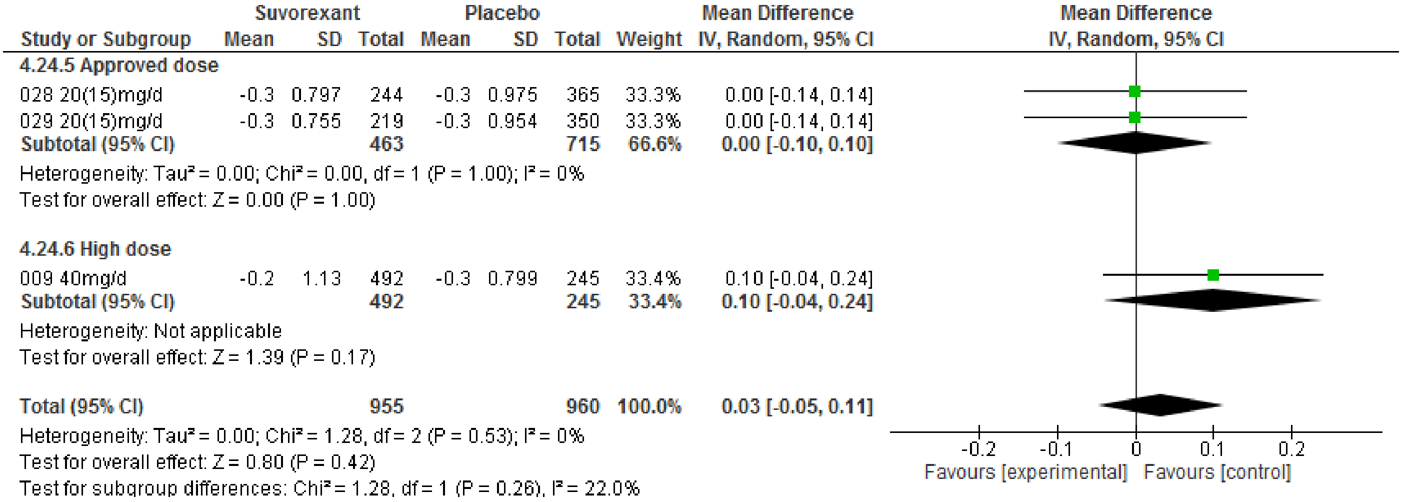

S1-17 Fig. Subjective number of awakenings at Month 3

Study or Subgroup	Suvorexant Mean	Suvorexant SD	Suvorexant Total	Placebo Mean	Placebo SD	Placebo Total	Weight	Mean Difference IV, Random, 95% CI
**4.25.5 Approved dose**								
028 20(15)mg/d	-0.4	0.77	228	-0.4	0.939	339	50.0%	0.00 [-0.14, 0.14]
029 20(15)mg/d	-0.5	0.716	197	-0.5	0.92	325	50.0%	0.00 [-0.14, 0.14]
**Subtotal (95% CI)**			**425**			**664**	**100.0%**	**0.00 [-0.10, 0.10]**
Heterogeneity: Tau² = 0.00; Chi² = 0.00, df = 1 (P = 1.00); I² = 0%								
Test for overall effect: Z = 0.00 (P = 1.00)								
**Total (95% CI)**			**425**			**664**	**100.0%**	**0.00 [-0.10, 0.10]**
Heterogeneity: Tau² = 0.00; Chi² = 0.00, df = 1 (P = 1.00); I² = 0%								
Test for overall effect: Z = 0.00 (P = 1.00)								
Test for subgroup differences: Not applicable								

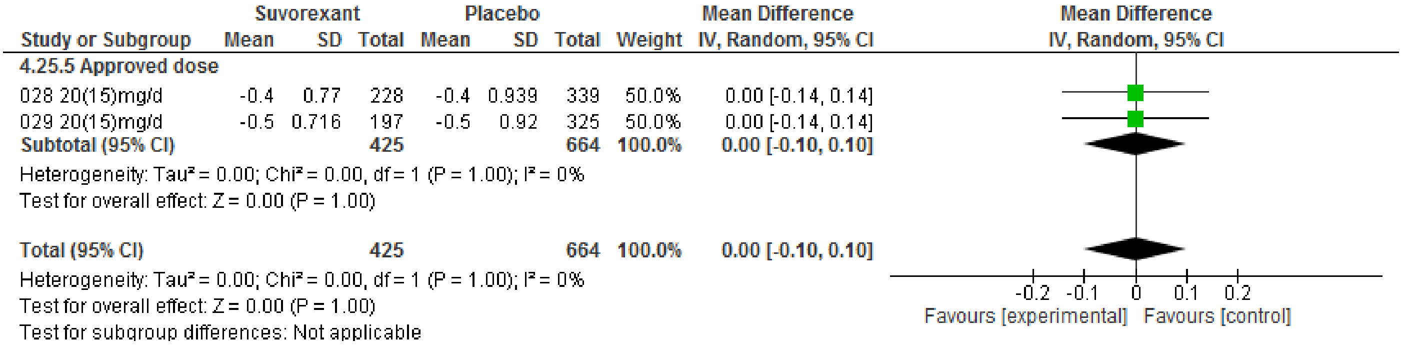

S1-18 Fig. Insomnia Severity Index (0–28 scale) at Month 1

	Suvorexant			Placebo				Mean Difference
**Study or Subgroup**	**Mean**	**SD**	**Total**	**Mean**	**SD**	**Total**	**Weight**	**IV, Random, 95% CI**
**4.26.5 Approved dose**								
028 20(15)mg/d	-4	3.96	241	-2.9	4.84	360	35.9%	-1.10 [-1.81, -0.39]
029 20(15)mg/d	-4.8	4.47	214	-3.2	4.75	347	29.5%	-1.60 [-2.38, -0.82]
**Subtotal (95% CI)**			**455**			**707**	**65.5%**	**-1.33 [-1.85, -0.80]**
Heterogeneity: Tau² = 0.00; Chi² = 0.87, df = 1 (P = 0.35); I² = 0%								
Test for overall effect: Z = 4.96 (P < 0.00001)								
**4.26.6 High dose**								
009 40mg/d	-3.6	4.53	492	-2.2	4.79	245	34.5%	-1.40 [-2.12, -0.68]
**Subtotal (95% CI)**			**492**			**245**	**34.5%**	**-1.40 [-2.12, -0.68]**
Heterogeneity: Not applicable								
Test for overall effect: Z = 3.81 (P = 0.0001)								
**Total (95% CI)**			**947**			**952**	**100.0%**	**-1.35 [-1.78, -0.93]**
Heterogeneity: Tau² = 0.00; Chi² = 0.89, df = 2 (P = 0.64); I² = 0%								
Test for overall effect: Z = 6.25 (P < 0.00001)								
Test for subgroup differences: Chi² = 0.03, df = 1 (P = 0.87), I² = 0%								

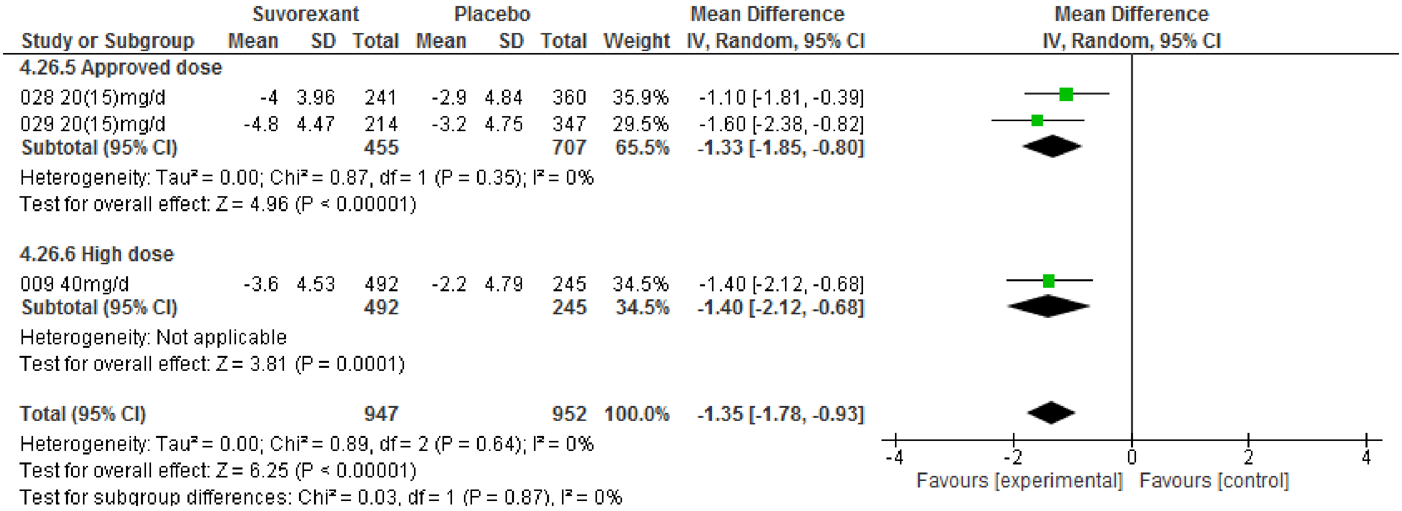

S1-19 Fig. Insomnia Severity Index (0–28 scale) at Month 3

	Suvorexant			Placebo				Mean Difference
**Study or Subgroup**	**Mean**	**SD**	**Total**	**Mean**	**SD**	**Total**	**Weight**	**IV, Random, 95% CI**
**4.27.5 Approved dose**								
028 20(15)mg/d	-5.7	4.62	228	-4.6	4.7	339	58.2%	-1.10 [-1.88, -0.32]
029 20(15)mg/d	-6.6	5.01	197	-5.3	5.48	320	41.8%	-1.30 [-2.22, -0.38]
**Subtotal (95% CI)**			**425**			**659**	**100.0%**	**-1.18 [-1.78, -0.59]**
Heterogeneity: Tau² = 0.00; Chi² = 0.11, df = 1 (P = 0.75); I² = 0%								
Test for overall effect: Z = 3.89 (P < 0.0001)								
**Total (95% CI)**			**425**			**659**	**100.0%**	**-1.18 [-1.78, -0.59]**
Heterogeneity: Tau² = 0.00; Chi² = 0.11, df = 1 (P = 0.75); I² = 0%								
Test for overall effect: Z = 3.89 (P < 0.0001)								
Test for subgroup differences: Not applicable								

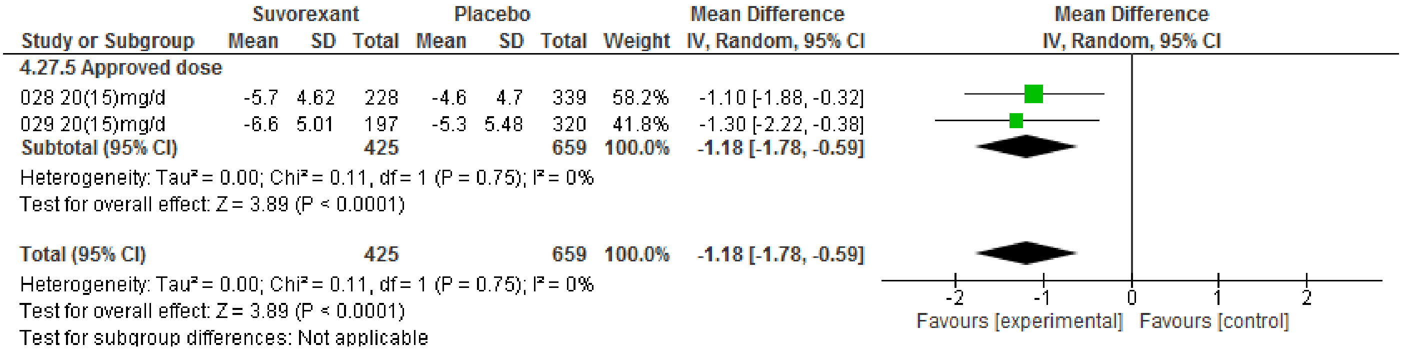

S1-20 Fig. Clinical Global Impression-Severity scale (1–7 scale) at Week 2

Study or Subgroup	Suvorexant Mean	Suvorexant SD	Suvorexant Total	Placebo Mean	Placebo SD	Placebo Total	Weight	Mean Difference IV, Random, 95% CI
**4.28.5 Approved dose**								
028 20(15)mg/d	-0.8	0.787	238	-0.4	0.967	359	50.0%	-0.40 [-0.54, -0.26]
029 20(15)mg/d	-0.8	0.743	212	-0.5	0.948	345	50.0%	-0.30 [-0.44, -0.16]
**Subtotal (95% CI)**			**450**			**704**	**100.0%**	**-0.35 [-0.45, -0.25]**
Heterogeneity: Tau² = 0.00; Chi² = 0.96, df = 1 (P = 0.33); I² = 0%								
Test for overall effect: Z = 6.86 (P < 0.00001)								
**Total (95% CI)**			**450**			**704**	**100.0%**	**-0.35 [-0.45, -0.25]**
Heterogeneity: Tau² = 0.00; Chi² = 0.96, df = 1 (P = 0.33); I² = 0%								
Test for overall effect: Z = 6.86 (P < 0.00001)								
Test for subgroup differences: Not applicable								

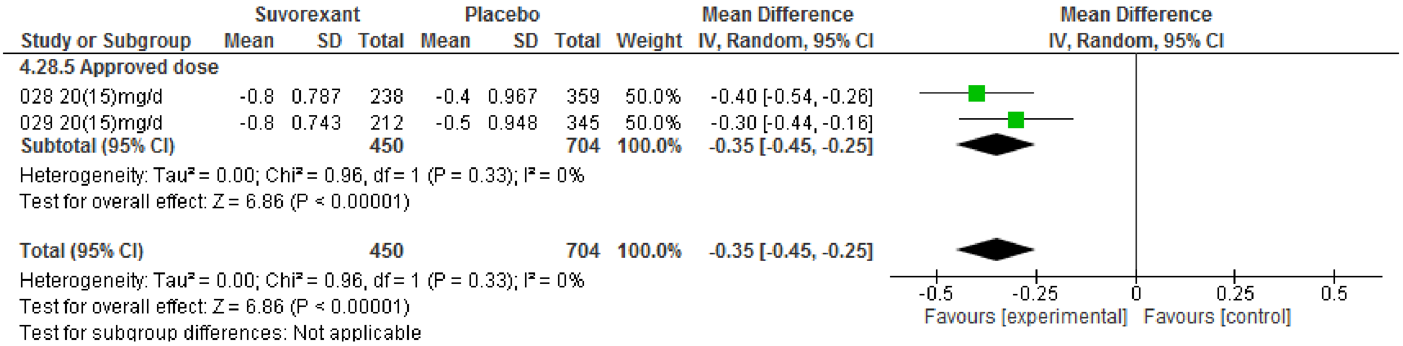

S1-21 Fig. Clinical Global Impression-Severity scale (1–7 scale) at Month 1

Study or Subgroup	Suvorexant Mean	Suvorexant SD	Suvorexant Total	Placebo Mean	Placebo SD	Placebo Total	Weight	Mean Difference IV, Random, 95% CI
**4.29.5 Approved dose**								
028 20(15)mg/d	-1	0.792	241	-0.6	0.968	360	33.3%	-0.40 [-0.54, -0.26]
029 20(15)mg/d	-1	0.745	213	-0.7	0.95	347	33.3%	-0.30 [-0.44, -0.16]
**Subtotal (95% CI)**			**454**			**707**	**66.6%**	**-0.35 [-0.45, -0.25]**
Heterogeneity: Tau² = 0.00; Chi² = 0.96, df = 1 (P = 0.33); I² = 0%								
Test for overall effect: Z = 6.86 (P < 0.00001)								
**4.29.6 High dose**								
009 40mg/d	-0.9	1.13	492	-0.5	0.799	245	33.4%	-0.40 [-0.54, -0.26]
**Subtotal (95% CI)**			**492**			**245**	**33.4%**	**-0.40 [-0.54, -0.26]**
Heterogeneity: Not applicable								
Test for overall effect: Z = 5.55 (P < 0.00001)								
**Total (95% CI)**			**946**			**952**	**100.0%**	**-0.37 [-0.45, -0.29]**
Heterogeneity: Tau² = 0.00; Chi² = 1.28, df = 2 (P = 0.53); I² = 0%								
Test for overall effect: Z = 8.80 (P < 0.00001)								
Test for subgroup differences: Chi² = 0.32, df = 1 (P = 0.57), I² = 0%								

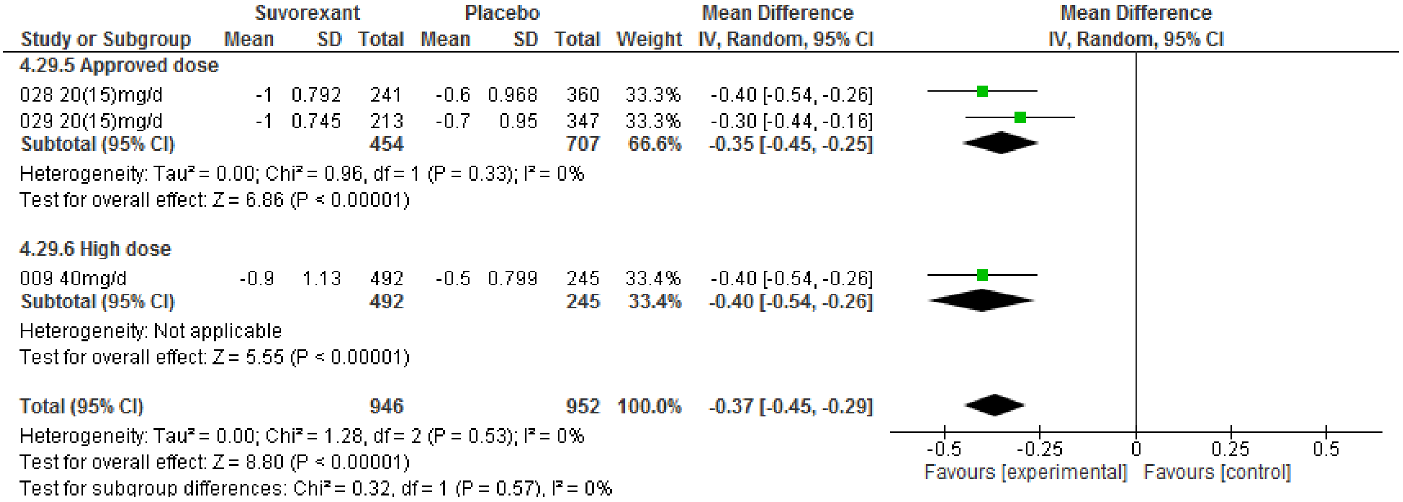

S1-22 Fig. Clinical Global Impression-Severity scale (1–7 scale) at Month 3

Study or Subgroup	Suvorexant Mean	Suvorexant SD	Suvorexant Total	Placebo Mean	Placebo SD	Placebo Total	Weight	Mean Difference IV, Random, 95% CI
**4.30.5 Approved dose**								
028 20(15)mg/d	-1.2	0.77	228	-0.9	0.939	339	50.0%	-0.30 [-0.44, -0.16]
029 20(15)mg/d	-1.3	0.716	197	-1	0.913	320	50.0%	-0.30 [-0.44, -0.16]
**Subtotal (95% CI)**			**425**			**659**	**100.0%**	**-0.30 [-0.40, -0.20]**
Heterogeneity: Tau² = 0.00; Chi² = 0.00, df = 1 (P = 1.00); I² = 0%								
Test for overall effect: Z = 5.88 (P < 0.00001)								
**Total (95% CI)**			**425**			**659**	**100.0%**	**-0.30 [-0.40, -0.20]**
Heterogeneity: Tau² = 0.00; Chi² = 0.00, df = 1 (P = 1.00); I² = 0%								
Test for overall effect: Z = 5.88 (P < 0.00001)								
Test for subgroup differences: Not applicable								

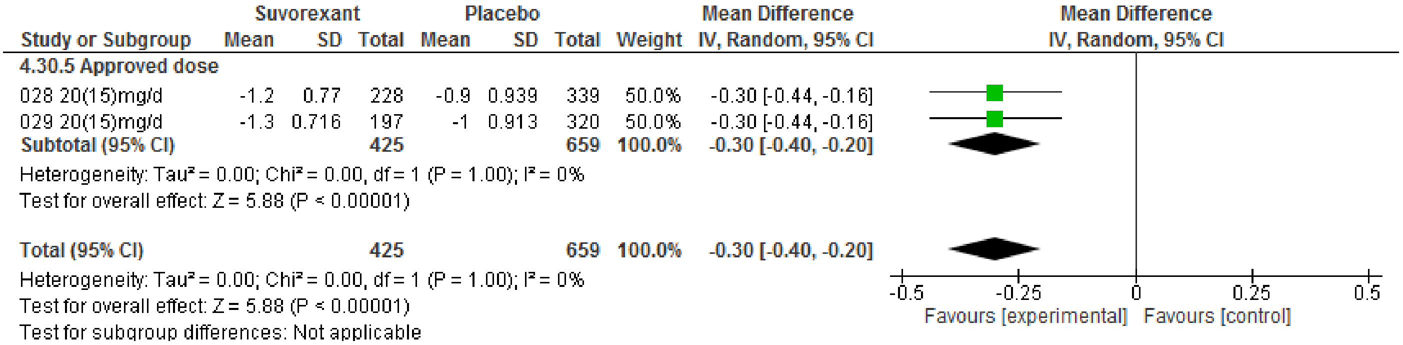

S1-23 Fig. Patient Global Impression-Severity scale (0–5 scale) at Week 2

Study or Subgroup	Suvorexant Mean	Suvorexant SD	Suvorexant Total	Placebo Mean	Placebo SD	Placebo Total	Weight	Mean Difference IV, Random, 95% CI
**4.31.5 Approved dose**								
028 20(15)mg/d	-0.6	0.787	238	-0.2	0.967	359	49.1%	-0.40 [-0.54, -0.26]
029 20(15)mg/d	-0.7	0.716	212	-0.4	0.948	345	50.9%	-0.30 [-0.44, -0.16]
**Subtotal (95% CI)**			**450**			**704**	**100.0%**	**-0.35 [-0.45, -0.25]**
Heterogeneity: Tau² = 0.00; Chi² = 0.98, df = 1 (P = 0.32); I² = 0%								
Test for overall effect: Z = 6.90 (P < 0.00001)								
**Total (95% CI)**			**450**			**704**	**100.0%**	**-0.35 [-0.45, -0.25]**
Heterogeneity: Tau² = 0.00; Chi² = 0.98, df = 1 (P = 0.32); I² = 0%								
Test for overall effect: Z = 6.90 (P < 0.00001)								
Test for subgroup differences: Not applicable								

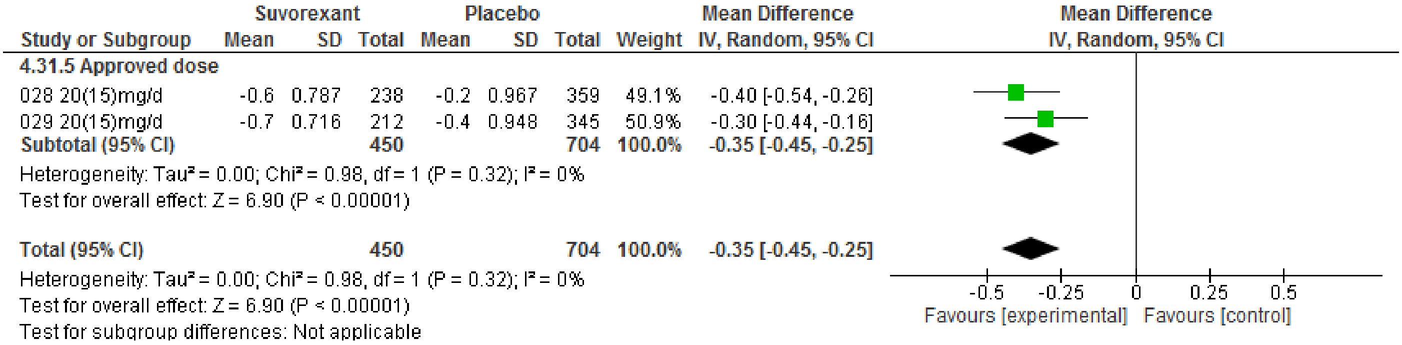

S1-24 Fig. Patient Global Impression-Severity scale (0–5 scale) at Month 1

Study or Subgroup	Suvorexant Mean	Suvorexant SD	Suvorexant Total	Placebo Mean	Placebo SD	Placebo Total	Weight	Mean Difference IV, Random, 95% CI
**4.32.5 Approved dose**								
028 20(15)mg/d	-0.7	0.792	241	-0.4	0.968	360	28.8%	-0.30 [-0.44, -0.16]
029 20(15)mg/d	-0.9	0.45	213	-0.5	0.95	347	42.3%	-0.40 [-0.52, -0.28]
**Subtotal (95% CI)**			**454**			**707**	**71.1%**	**-0.36 [-0.45, -0.26]**
Heterogeneity: Tau² = 0.00; Chi² = 1.14, df = 1 (P = 0.29); I² = 12%								
Test for overall effect: Z = 7.27 (P < 0.00001)								
**4.32.6 High dose**								
009 40mg/d	-0.8	1.13	492	-0.5	0.799	245	28.9%	-0.30 [-0.44, -0.16]
**Subtotal (95% CI)**			**492**			**245**	**28.9%**	**-0.30 [-0.44, -0.16]**
Heterogeneity: Not applicable								
Test for overall effect: Z = 4.16 (P < 0.0001)								
**Total (95% CI)**			**946**			**952**	**100.0%**	**-0.34 [-0.42, -0.27]**
Heterogeneity: Tau² = 0.00; Chi² = 1.63, df = 2 (P = 0.44); I² = 0%								
Test for overall effect: Z = 8.83 (P < 0.00001)								
Test for subgroup differences: Chi² = 0.44, df = 1 (P = 0.50), I² = 0%								

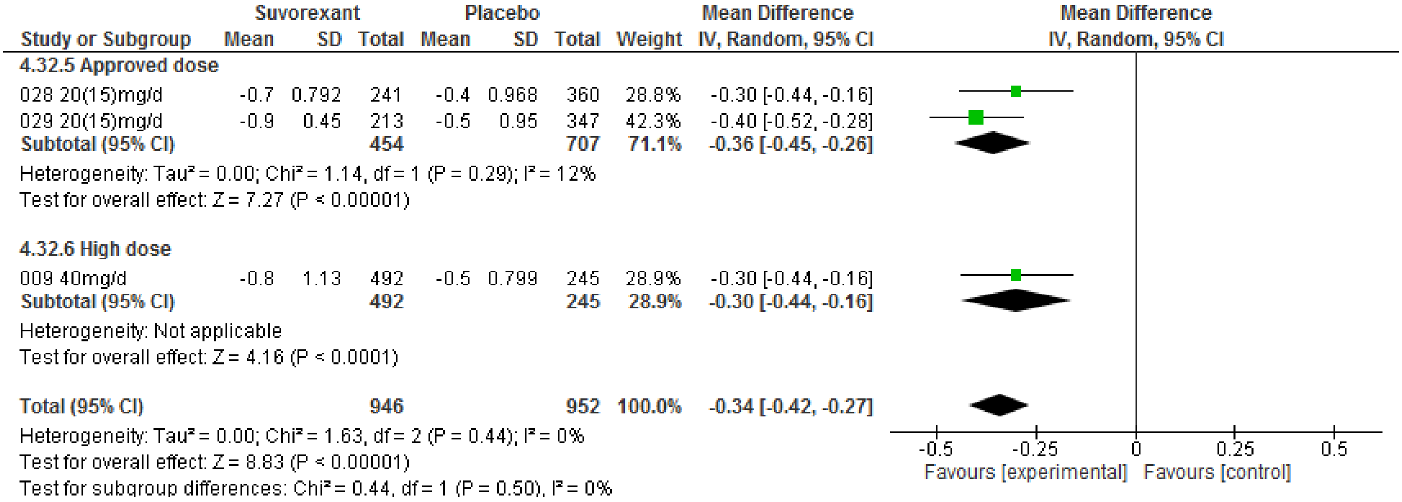

S1-25 Fig. Patient Global Impression-Severity scale (0–5 scale) at Month 3

Study or Subgroup	Suvorexant Mean	Suvorexant SD	Suvorexant Total	Placebo Mean	Placebo SD	Placebo Total	Weight	Mean Difference IV, Random, 95% CI
**4.33.5 Approved dose**								
028 20(15)mg/d	-1	0.77	228	-0.7	0.939	339	71.4%	-0.30 [-0.44, -0.16]
029 20(15)mg/d	-1.2	1.43	197	-0.9	0.913	320	28.6%	-0.30 [-0.52, -0.08]
**Subtotal (95% CI)**			**425**			**659**	**100.0%**	**-0.30 [-0.42, -0.18]**
Heterogeneity: Tau² = 0.00; Chi² = 0.00, df = 1 (P = 1.00); I² = 0%								
Test for overall effect: Z = 4.92 (P < 0.00001)								
**Total (95% CI)**			**425**			**659**	**100.0%**	**-0.30 [-0.42, -0.18]**
Heterogeneity: Tau² = 0.00; Chi² = 0.00, df = 1 (P = 1.00); I² = 0%								
Test for overall effect: Z = 4.92 (P < 0.00001)								
Test for subgroup differences: Not applicable								

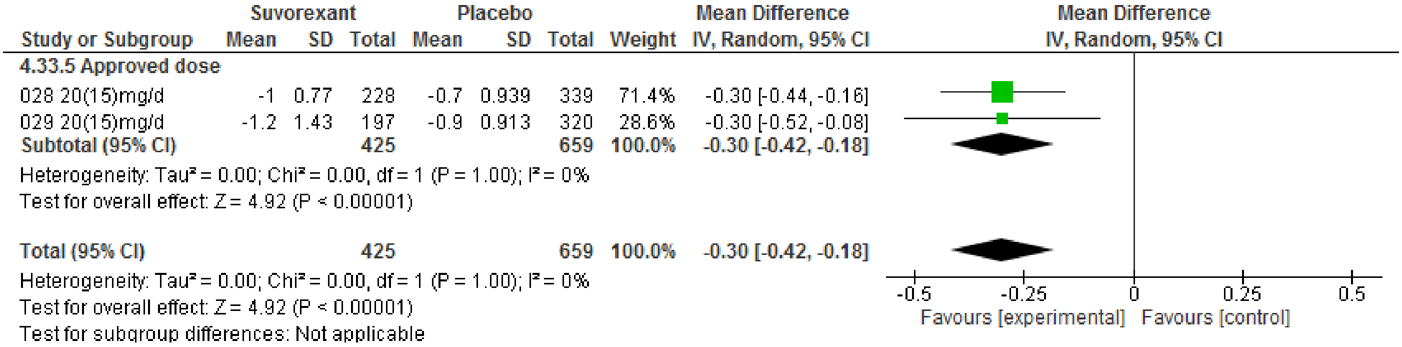

S1-26 Fig. Clinical Global Impression-Improvement scale (1–7 scale) at Week 2

Study or Subgroup	Suvorexant Mean	Suvorexant SD	Suvorexant Total	Placebo Mean	Placebo SD	Placebo Total	Weight	Mean Difference IV, Random, 95% CI
**4.34.5 Approved dose**								
028 20(15)mg/d	3	1.57	238	3.4	0.967	359	28.7%	-0.40 [-0.62, -0.18]
029 20(15)mg/d	3	0.743	212	3.3	0.948	345	71.3%	-0.30 [-0.44, -0.16]
**Subtotal (95% CI)**			**450**			**704**	**100.0%**	**-0.33 [-0.45, -0.21]**
Heterogeneity: $Tau^2 = 0.00$; $Chi^2 = 0.55$, df = 1 (P = 0.46); $I^2 = 0\%$								
Test for overall effect: Z = 5.39 (P < 0.00001)								
**Total (95% CI)**			**450**			**704**	**100.0%**	**-0.33 [-0.45, -0.21]**
Heterogeneity: $Tau^2 = 0.00$; $Chi^2 = 0.55$, df = 1 (P = 0.46); $I^2 = 0\%$								
Test for overall effect: Z = 5.39 (P < 0.00001)								
Test for subgroup differences: Not applicable								

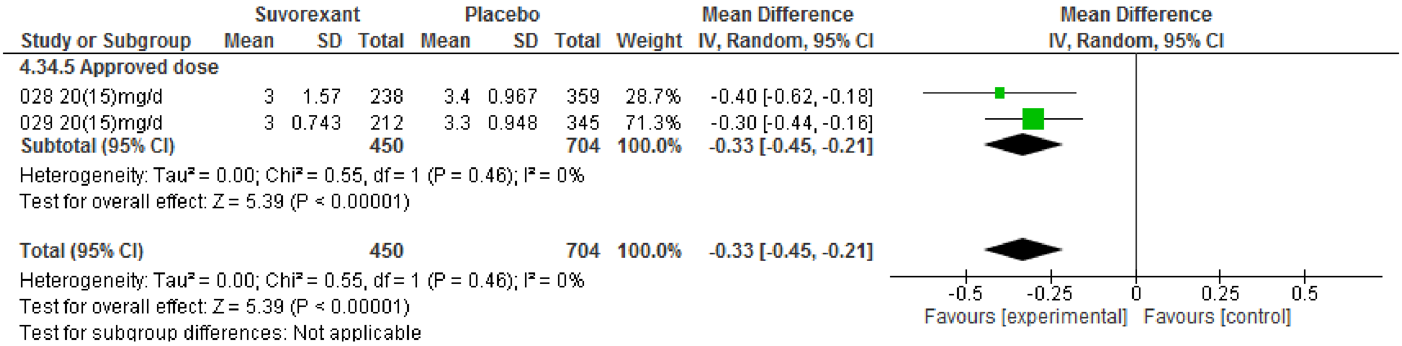

S1-27 Fig. Clinical Global Impression-Improvement scale (1–7 scale) at Month 1

Study or Subgroup	Suvorexant Mean	Suvorexant SD	Suvorexant Total	Placebo Mean	Placebo SD	Placebo Total	Weight	Mean Difference IV, Random, 95% CI
**4.35.5 Approved dose**								
028 20(15)mg/d	2.9	1.58	241	3.3	0.968	360	16.7%	-0.40 [-0.62, -0.18]
029 20(15)mg/d	2.9	0.745	213	3.2	0.95	347	41.6%	-0.30 [-0.44, -0.16]
**Subtotal (95% CI)**			**454**			**707**	**58.3%**	**-0.33 [-0.45, -0.21]**
Heterogeneity: $Tau^2 = 0.00$; $Chi^2 = 0.55$, df = 1 (P = 0.46); $I^2 = 0\%$								
Test for overall effect: Z = 5.39 (P < 0.00001)								
**4.35.6 High dose**								
009 40mg/d	2.9	1.13	492	3.3	0.799	245	41.7%	-0.40 [-0.54, -0.26]
**Subtotal (95% CI)**			**492**			**245**	**41.7%**	**-0.40 [-0.54, -0.26]**
Heterogeneity: Not applicable								
Test for overall effect: Z = 5.55 (P < 0.00001)								
**Total (95% CI)**			**946**			**952**	**100.0%**	**-0.36 [-0.45, -0.27]**
Heterogeneity: $Tau^2 = 0.00$; $Chi^2 = 1.12$, df = 2 (P = 0.57); $I^2 = 0\%$								
Test for overall effect: Z = 7.70 (P < 0.00001)								
Test for subgroup differences: $Chi^2 = 0.57$, df = 1 (P = 0.45), $I^2 = 0\%$								

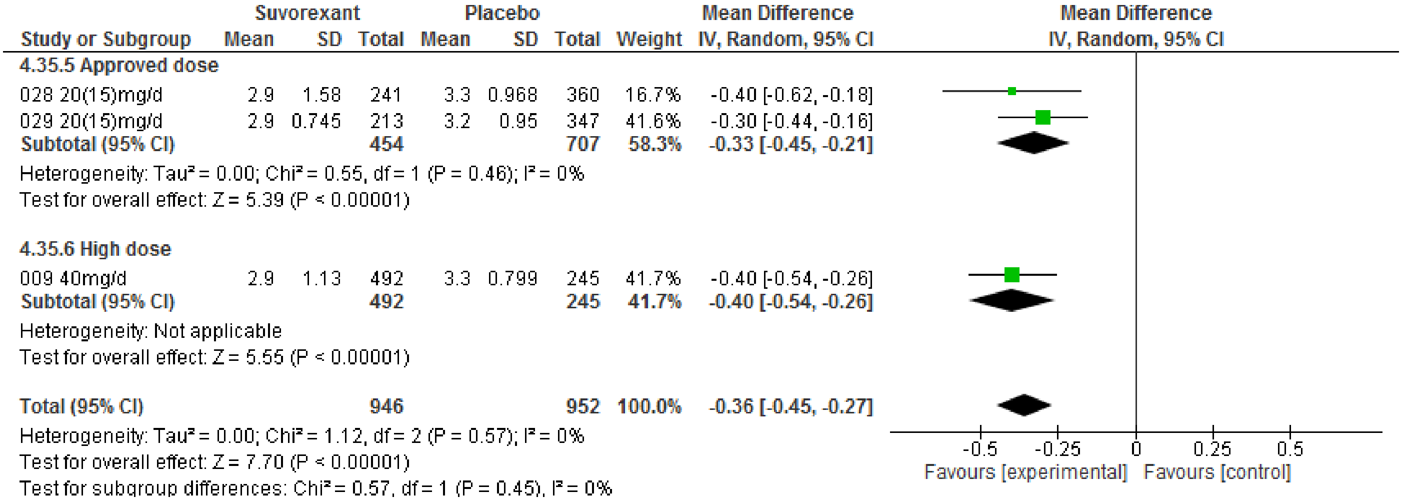

S1-28 Fig. Clinical Global Impression-Improvement scale (1–7 scale) at Month 3

Study or Subgroup	Suvorexant Mean	Suvorexant SD	Suvorexant Total	Placebo Mean	Placebo SD	Placebo Total	Weight	Mean Difference IV, Random, 95% CI
**4.36.5 Approved dose**								
028 20(15)mg/d	2.6	1.54	228	3	0.939	339	50.0%	-0.40 [-0.62, -0.18]
029 20(15)mg/d	2.5	1.43	197	3	0.913	320	50.0%	-0.50 [-0.72, -0.28]
**Subtotal (95% CI)**			**425**			**659**	**100.0%**	**-0.45 [-0.61, -0.29]**
Heterogeneity: $Tau^2 = 0.00$; $Chi^2 = 0.38$, df = 1 (P = 0.54); $I^2 = 0\%$								
Test for overall effect: Z = 5.58 (P < 0.00001)								
**Total (95% CI)**			**425**			**659**	**100.0%**	**-0.45 [-0.61, -0.29]**
Heterogeneity: $Tau^2 = 0.00$; $Chi^2 = 0.38$, df = 1 (P = 0.54); $I^2 = 0\%$								
Test for overall effect: Z = 5.58 (P < 0.00001)								
Test for subgroup differences: Not applicable								

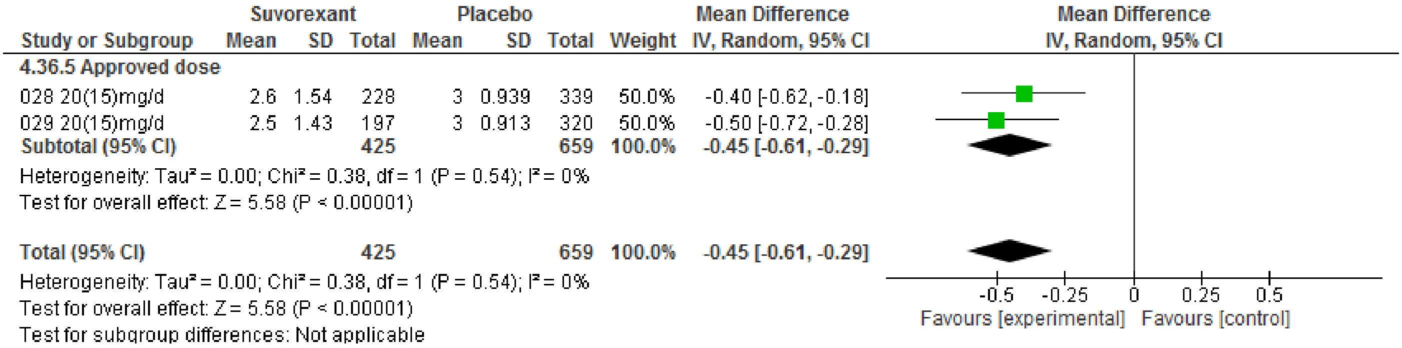

S1-29 Fig. Patient Global Impression-Improvement scale (1–7 scale) at Week 2

Study or Subgroup	Suvorexant Mean	Suvorexant SD	Suvorexant Total	Placebo Mean	Placebo SD	Placebo Total	Weight	Mean Difference IV, Random, 95% CI
**4.37.5 Approved dose**								
028 20(15)mg/d	3	0.787	238	3.4	0.967	359	71.5%	-0.40 [-0.54, -0.26]
029 20(15)mg/d	3	1.49	212	3.3	0.948	345	28.5%	-0.30 [-0.52, -0.08]
**Subtotal (95% CI)**			**450**			**704**	**100.0%**	**-0.37 [-0.49, -0.25]**

Heterogeneity: Tau² = 0.00; Chi² = 0.55, df = 1 (P = 0.46); I² = 0%
Test for overall effect: Z = 6.09 (P < 0.00001)

**Total (95% CI)**			**450**			**704**	**100.0%**	**-0.37 [-0.49, -0.25]**

Heterogeneity: Tau² = 0.00; Chi² = 0.55, df = 1 (P = 0.46); I² = 0%
Test for overall effect: Z = 6.09 (P < 0.00001)
Test for subgroup differences: Not applicable

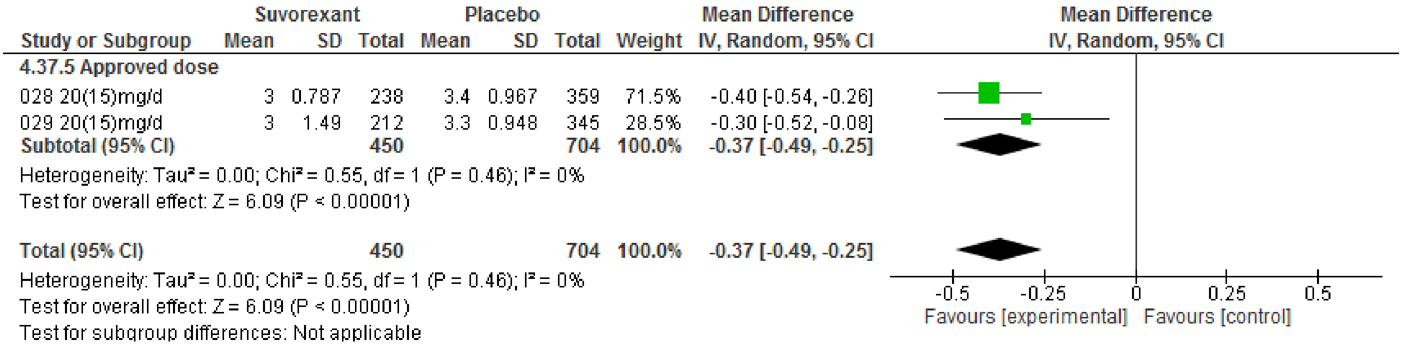

S1-30 Fig. Patient Global Impression-Improvement scale (1–7 scale) at Month 1

Study or Subgroup	Suvorexant Mean	Suvorexant SD	Suvorexant Total	Placebo Mean	Placebo SD	Placebo Total	Weight	Mean Difference IV, Random, 95% CI
**4.38.5 Approved dose**								
028 20(15)mg/d	2.8	1.58	241	3.3	0.968	360	24.1%	-0.50 [-0.72, -0.28]
029 20(15)mg/d	2.8	0.745	213	3.2	0.95	347	51.9%	-0.40 [-0.54, -0.26]
**Subtotal (95% CI)**			**454**			**707**	**76.0%**	**-0.43 [-0.55, -0.31]**

Heterogeneity: Tau² = 0.00; Chi² = 0.55, df = 1 (P = 0.46); I² = 0%
Test for overall effect: Z = 7.03 (P < 0.00001)

**4.38.6 High dose**								
009 40mg/d	2.8	2.26	492	3.4	0.799	245	24.0%	-0.60 [-0.82, -0.38]
**Subtotal (95% CI)**			**492**			**245**	**24.0%**	**-0.60 [-0.82, -0.38]**

Heterogeneity: Not applicable
Test for overall effect: Z = 5.26 (P < 0.00001)

**Total (95% CI)**			**946**			**952**	**100.0%**	**-0.47 [-0.59, -0.36]**

Heterogeneity: Tau² = 0.00; Chi² = 2.31, df = 2 (P = 0.32); I² = 13%
Test for overall effect: Z = 8.00 (P < 0.00001)
Test for subgroup differences: Chi² = 1.76, df = 1 (P = 0.18), I² = 43.1%

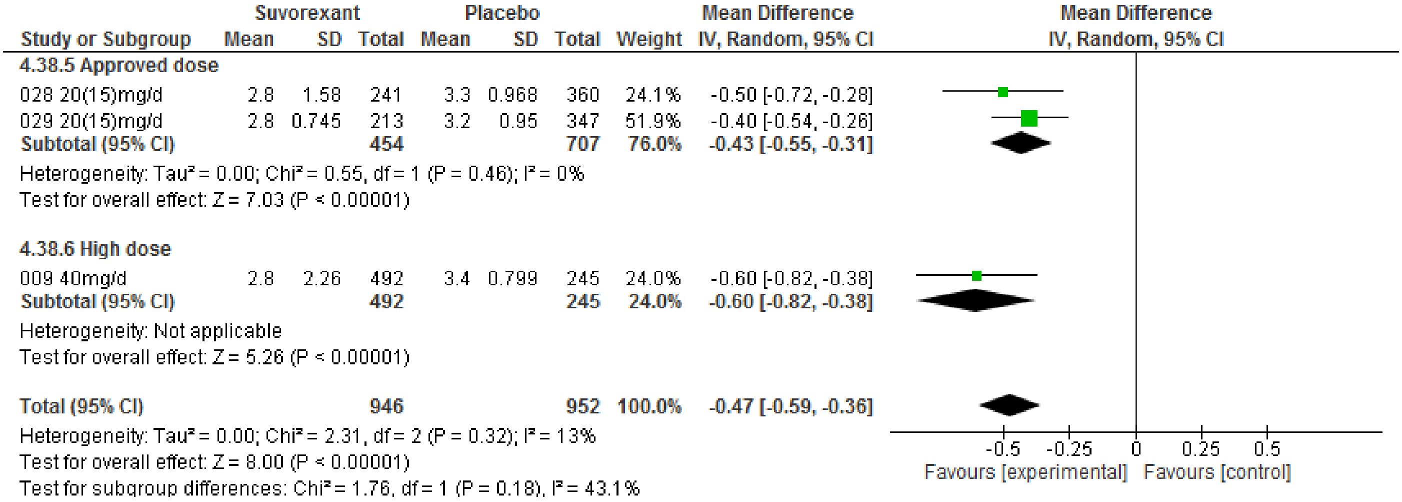

S1-31 Fig. Patient Global Impression-Improvement scale (1–7 scale) at Month 3

Study or Subgroup	Suvorexant Mean	Suvorexant SD	Suvorexant Total	Placebo Mean	Placebo SD	Placebo Total	Weight	Mean Difference IV, Random, 95% CI
**4.39.5 Approved dose**								
028 20(15)mg/d	2.5	1.54	228	2.9	0.939	339	28.6%	-0.40 [-0.62, -0.18]
029 20(15)mg/d	2.6	0.716	197	2.9	0.913	320	71.4%	-0.30 [-0.44, -0.16]
**Subtotal (95% CI)**			**425**			**659**	**100.0%**	**-0.33 [-0.45, -0.21]**

Heterogeneity: Tau² = 0.00; Chi² = 0.55, df = 1 (P = 0.46); I² = 0%
Test for overall effect: Z = 5.39 (P < 0.00001)

**Total (95% CI)**			**425**			**659**	**100.0%**	**-0.33 [-0.45, -0.21]**

Heterogeneity: Tau² = 0.00; Chi² = 0.55, df = 1 (P = 0.46); I² = 0%
Test for overall effect: Z = 5.39 (P < 0.00001)
Test for subgroup differences: Not applicable

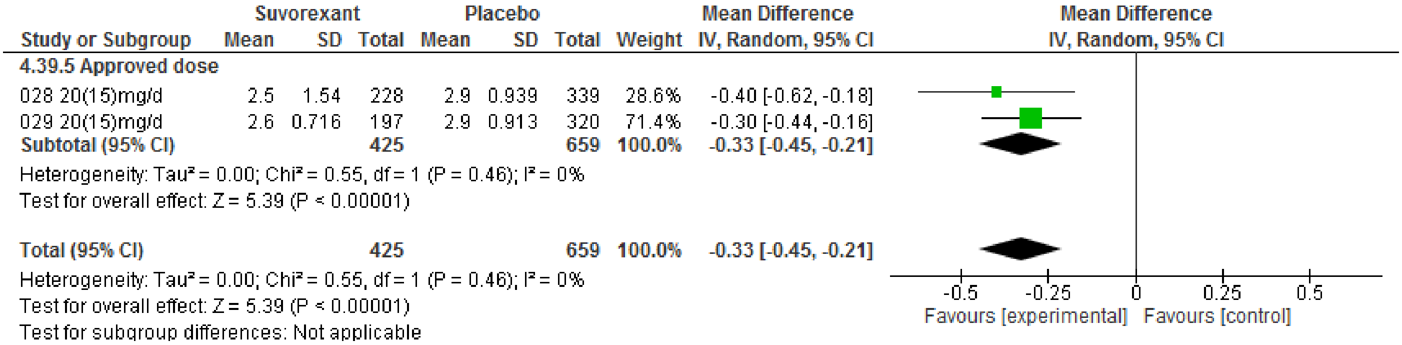

S1-32 Fig. Response rate at Month 3

Study or Subgroup	Suvorexant Events	Suvorexant Total	Placebo Events	Placebo Total	Weight	Risk Ratio M-H, Random, 95% CI
**5.40.3 ALL**						
028 all dose	267	550	199	328	58.2%	0.80 [0.71, 0.90]
029 all dose	212	517	170	310	41.8%	0.75 [0.65, 0.86]
**Subtotal (95% CI)**		**1067**		**638**	**100.0%**	**0.78 [0.71, 0.85]**
Total events	479		369			
Heterogeneity: Tau² = 0.00; Chi² = 0.49, df = 1 (P = 0.48); I² = 0%						
Test for overall effect: Z = 5.27 (P < 0.00001)						
**Total (95% CI)**		**1067**		**638**	**100.0%**	**0.78 [0.71, 0.85]**
Total events	479		369			
Heterogeneity: Tau² = 0.00; Chi² = 0.49, df = 1 (P = 0.48); I² = 0%						
Test for overall effect: Z = 5.27 (P < 0.00001)						
Test for subgroup differences: Not applicable						

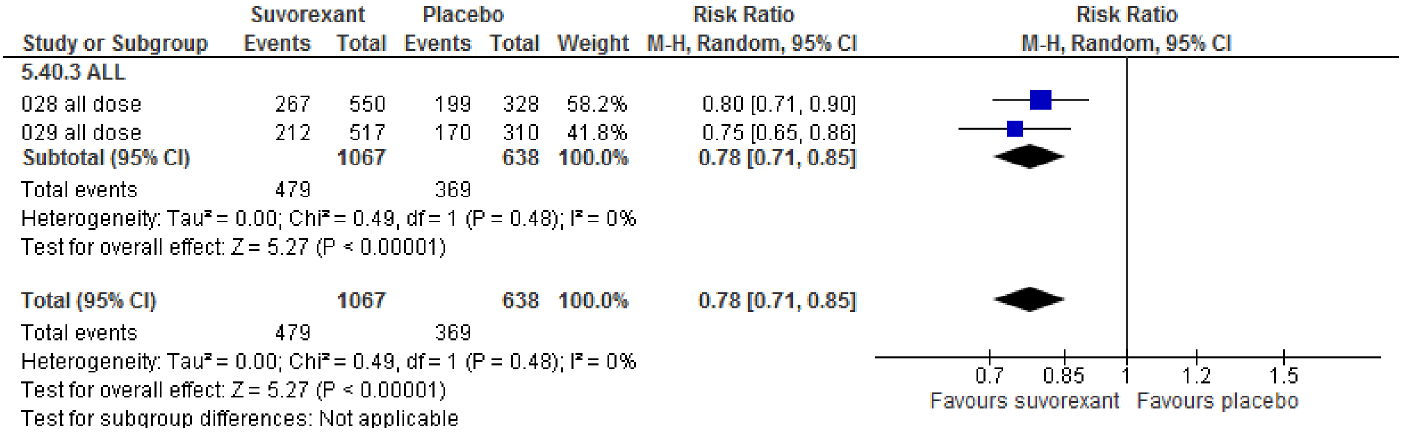

S1-33 Fig. Latency to onset of persistent sleep (minutes) at Day 1

Study or Subgroup	Suvorexant Mean	Suvorexant SD	Suvorexant Total	Placebo Mean	Placebo SD	Placebo Total	Weight	Mean Difference IV, Random, 95% CI
**4.10.5 Approved dose**								
006 20mg/d	39.6	42.57	33	57	48.35	127	6.6%	-17.40 [-34.18, -0.62]
028 20(15)mg/d	-29.9	29.06	193	-20.3	28.67	290	67.0%	-9.60 [-14.86, -4.34]
029 20(15)mg/d	-25.3	41.63	144	-13	42.13	284	26.4%	-12.30 [-20.68, -3.92]
**Subtotal (95% CI)**			**370**			**701**	**100.0%**	**-10.83 [-15.13, -6.52]**
Heterogeneity: Tau² = 0.00; Chi² = 0.92, df = 2 (P = 0.63); I² = 0%								
Test for overall effect: Z = 4.93 (P < 0.00001)								
**Total (95% CI)**			**370**			**701**	**100.0%**	**-10.83 [-15.13, -6.52]**
Heterogeneity: Tau² = 0.00; Chi² = 0.92, df = 2 (P = 0.63); I² = 0%								
Test for overall effect: Z = 4.93 (P < 0.00001)								
Test for subgroup differences: Not applicable								

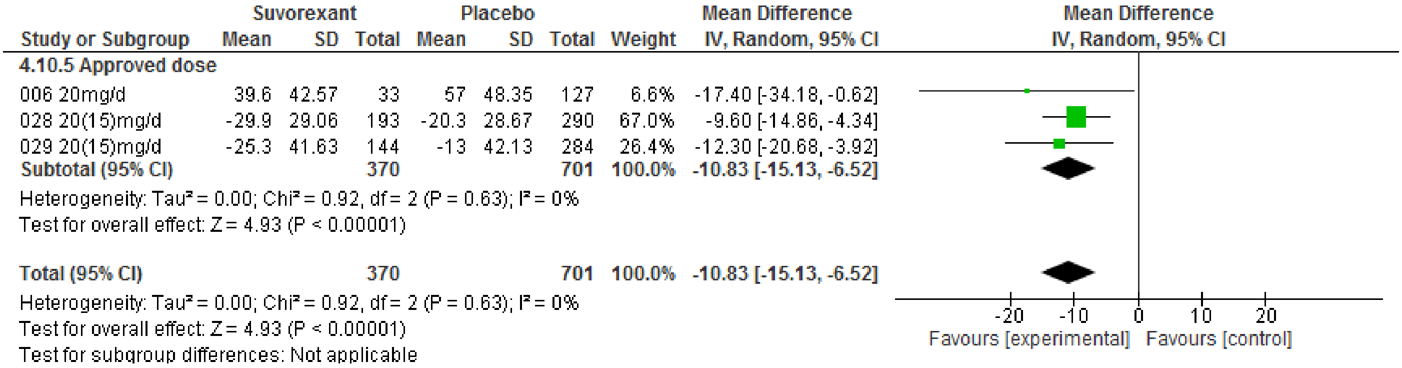

S1-34 Fig. Latency to onset of persistent sleep (minutes) at Month 1

Study or Subgroup	Suvorexant Mean	Suvorexant SD	Suvorexant Total	Placebo Mean	Placebo SD	Placebo Total	Weight	Mean Difference IV, Random, 95% CI
**4.11.5 Approved dose**								
006 20mg/d	27.7	42.93	31	52.4	47.71	116	10.2%	-24.70 [-42.13, -7.27]
028 20(15)mg/d	-33.6	30.53	185	-23.3	30.29	272	50.3%	-10.30 [-15.98, -4.62]
029 20(15)mg/d	-32.5	34.13	133	-24.6	35.28	271	39.5%	-7.90 [-15.06, -0.74]
**Subtotal (95% CI)**			**349**			**659**	**100.0%**	**-10.82 [-16.72, -4.93]**
Heterogeneity: Tau² = 9.58; Chi² = 3.05, df = 2 (P = 0.22); I² = 35%								
Test for overall effect: Z = 3.60 (P = 0.0003)								
**Total (95% CI)**			**349**			**659**	**100.0%**	**-10.82 [-16.72, -4.93]**
Heterogeneity: Tau² = 9.58; Chi² = 3.05, df = 2 (P = 0.22); I² = 35%								
Test for overall effect: Z = 3.60 (P = 0.0003)								
Test for subgroup differences: Not applicable								

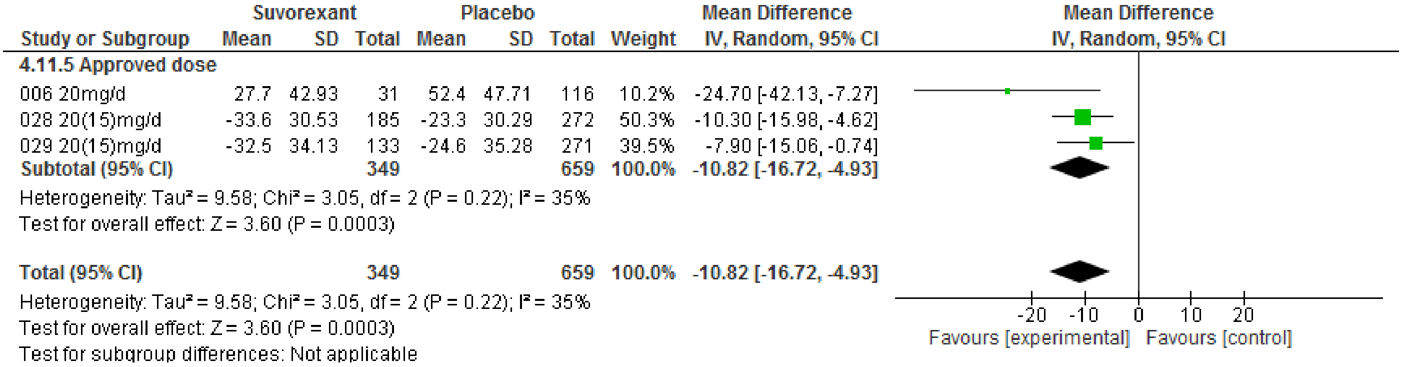

S1-35 Fig. Latency to onset of persistent sleep (minutes) at Month 3

Study or Subgroup	Suvorexant Mean	Suvorexant SD	Suvorexant Total	Placebo Mean	Placebo SD	Placebo Total	Weight	Mean Difference IV, Random, 95% CI
**4.12.5 Approved dose**								
028 20(15)mg/d	-34.7	30.11	172	-26.6	29.91	251	56.3%	-8.10 [-13.93, -2.27]
029 20(15)mg/d	-28.9	37.37	127	-28.6	37.48	255	43.7%	-0.30 [-8.26, 7.66]
**Subtotal (95% CI)**			**299**			**506**	**100.0%**	**-4.69 [-12.27, 2.89]**
Heterogeneity: Tau² = 17.75; Chi² = 2.40, df = 1 (P = 0.12); I² = 58%								
Test for overall effect: Z = 1.21 (P = 0.23)								
**Total (95% CI)**			**299**			**506**	**100.0%**	**-4.69 [-12.27, 2.89]**
Heterogeneity: Tau² = 17.75; Chi² = 2.40, df = 1 (P = 0.12); I² = 58%								
Test for overall effect: Z = 1.21 (P = 0.23)								
Test for subgroup differences: Not applicable								

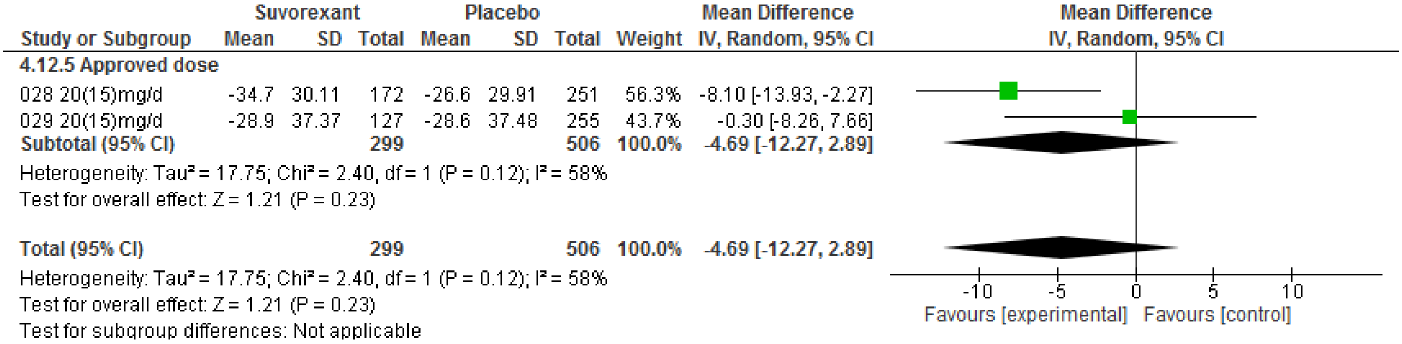

S1-36 Fig. Wakefulness after persistent sleep onset (minutes) at Day 1

Study or Subgroup	Suvorexant Mean	Suvorexant SD	Suvorexant Total	Placebo Mean	Placebo SD	Placebo Total	Weight	Mean Difference IV, Random, 95% CI
**4.13.5 Approved dose**								
028 20(15)mg/d	-52.1	37.47	192	-19.6	37.17	287	59.0%	-32.50 [-39.33, -25.67]
029 20(15)mg/d	-58.3	41.02	144	-21.3	40.34	283	41.0%	-37.00 [-45.18, -28.82]
**Subtotal (95% CI)**			**336**			**570**	**100.0%**	**-34.35 [-39.59, -29.10]**
Heterogeneity: Tau² = 0.00; Chi² = 0.69, df = 1 (P = 0.41); I² = 0%								
Test for overall effect: Z = 12.84 (P < 0.00001)								
**Total (95% CI)**			**336**			**570**	**100.0%**	**-34.35 [-39.59, -29.10]**
Heterogeneity: Tau² = 0.00; Chi² = 0.69, df = 1 (P = 0.41); I² = 0%								
Test for overall effect: Z = 12.84 (P < 0.00001)								
Test for subgroup differences: Not applicable								

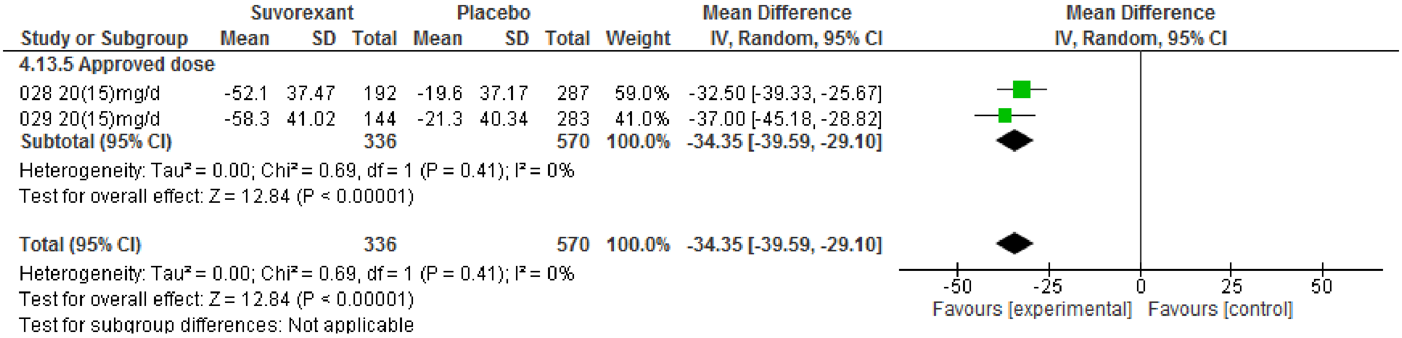

S1-37 Fig. Wakefulness after persistent sleep onset (minutes) at Month 1

Study or Subgroup	Suvorexant Mean	Suvorexant SD	Suvorexant Total	Placebo Mean	Placebo SD	Placebo Total	Weight	Mean Difference IV, Random, 95% CI
**4.14.5 Approved dose**								
028 20(15)mg/d	-45	42.33	185	-18.7	42.91	272	55.6%	-26.30 [-34.25, -18.35]
029 20(15)mg/d	-46.6	42.79	132	-22.5	42.76	270	44.4%	-24.10 [-33.01, -15.19]
**Subtotal (95% CI)**			**317**			**542**	**100.0%**	**-25.32 [-31.25, -19.39]**
Heterogeneity: Tau² = 0.00; Chi² = 0.13, df = 1 (P = 0.72); I² = 0%								
Test for overall effect: Z = 8.37 (P < 0.00001)								
**Total (95% CI)**			**317**			**542**	**100.0%**	**-25.32 [-31.25, -19.39]**
Heterogeneity: Tau² = 0.00; Chi² = 0.13, df = 1 (P = 0.72); I² = 0%								
Test for overall effect: Z = 8.37 (P < 0.00001)								
Test for subgroup differences: Not applicable								

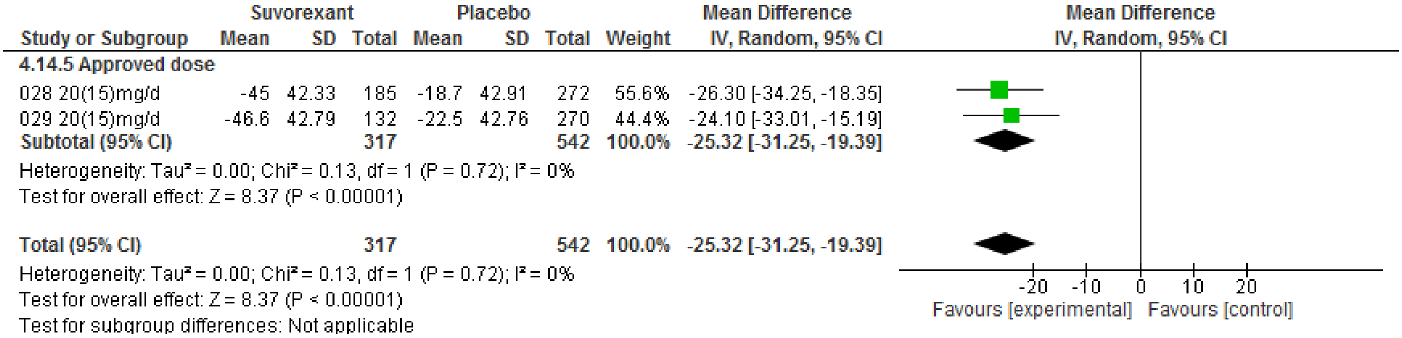

S1-38 Fig. Wakefulness after persistent sleep onset (minutes) at Month 3

Study or Subgroup	Suvorexant Mean	Suvorexant SD	Suvorexant Total	Placebo Mean	Placebo SD	Placebo Total	Weight	Mean Difference IV, Random, 95% CI
**4.15.5 Approved dose**								
028 20(15)mg/d	-41.6	42.82	172	-25	42.03	251	50.8%	-16.60 [-24.85, -8.35]
029 20(15)mg/d	-56	42.55	127	-24.8	42.12	252	49.2%	-31.20 [-40.24, -22.16]
**Subtotal (95% CI)**			**299**			**503**	**100.0%**	**-23.78 [-38.08, -9.47]**
Heterogeneity: Tau² = 87.08; Chi² = 5.47, df = 1 (P = 0.02); I² = 82%								
Test for overall effect: Z = 3.26 (P = 0.001)								
**Total (95% CI)**			**299**			**503**	**100.0%**	**-23.78 [-38.08, -9.47]**
Heterogeneity: Tau² = 87.08; Chi² = 5.47, df = 1 (P = 0.02); I² = 82%								
Test for overall effect: Z = 3.26 (P = 0.001)								
Test for subgroup differences: Not applicable								

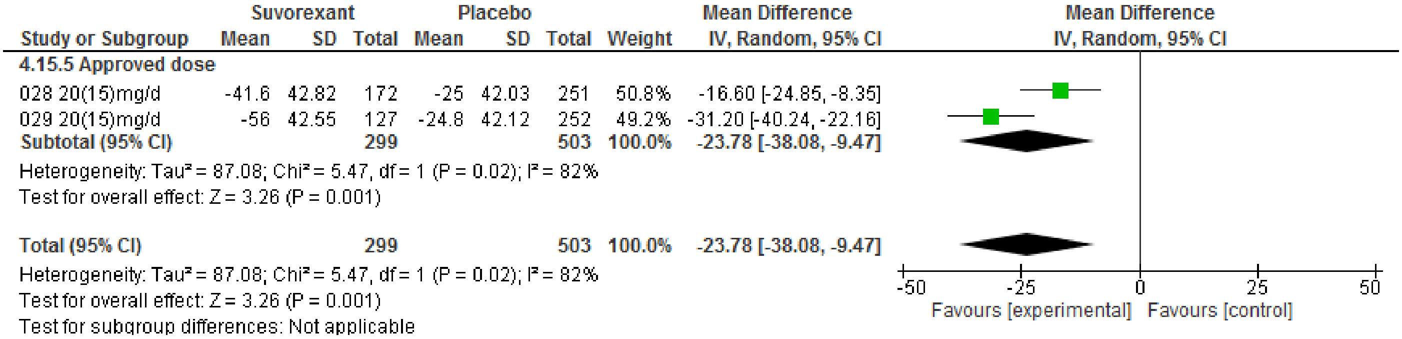

Supplement: S1 Fig — (PDF) [file pone.0136910.s002.pdf]
