# Supplementary material for: Suvorexant for Primary Insomnia: A Systematic Review and Meta-Analysis of Randomized Placebo-Controlled Trials
Source: PLoS One. 2015 Aug 28;10(8):e0136910. doi: 10.1371/journal.pone.0136910 (PMC4552781; doi:10.1371/journal.pone.0136910)

S2 Fig. Forest plots of safety secondary outcomes.

S2-1 Fig. Discontinuation due to all cause

Study or Subgroup	Suvorexant Events	Suvorexant Total	Placebo Events	Placebo Total	Weight	Risk Ratio M-H, Random, 95% CI
**5.1.3 ALL**						
006 all dose	7	127	9	127	2.4%	0.78 [0.30, 2.02]
009 40mg/d	200	522	97	259	60.6%	1.02 [0.84, 1.24]
028 all dose	62	637	43	385	16.4%	0.87 [0.60, 1.26]
029 all dose	75	632	53	387	20.6%	0.87 [0.62, 1.20]
**Subtotal (95% CI)**		**1918**		**1158**	**100.0%**	**0.96 [0.82, 1.11]**
Total events	344		202			
Heterogeneity: Tau² = 0.00; Chi² = 1.28, df = 3 (P = 0.73); I² = 0%						
Test for overall effect: Z = 0.58 (P = 0.56)						
**Total (95% CI)**		**1918**		**1158**	**100.0%**	**0.96 [0.82, 1.11]**
Total events	344		202			
Heterogeneity: Tau² = 0.00; Chi² = 1.28, df = 3 (P = 0.73); I² = 0%						
Test for overall effect: Z = 0.58 (P = 0.56)						
Test for subgroup differences: Not applicable						

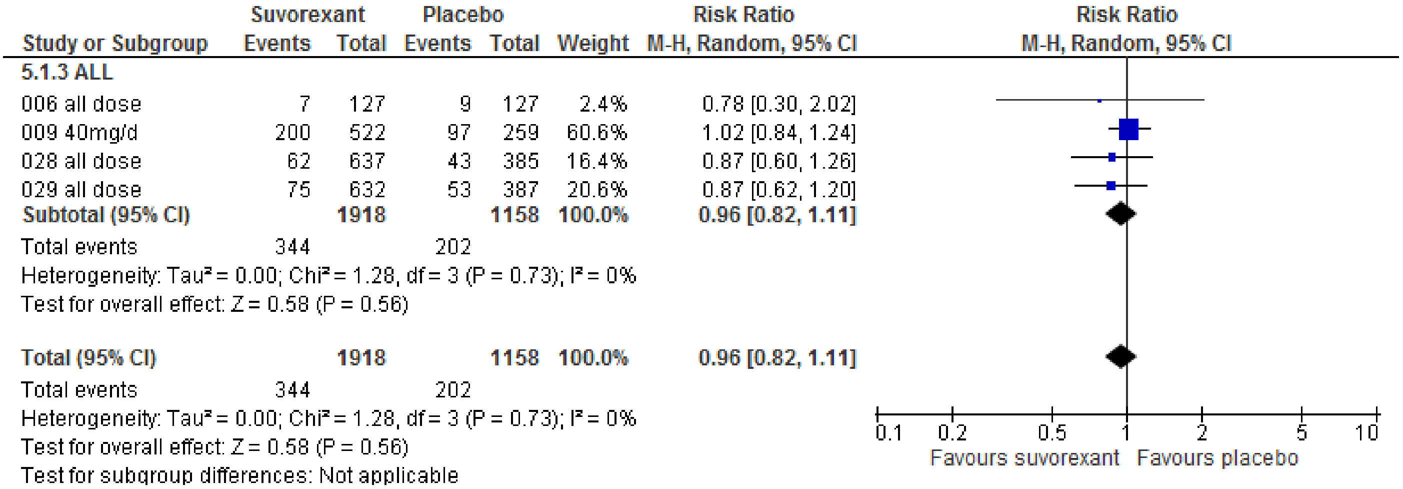

S2-2 Fig. Discontinuation due to intolerability

Study or Subgroup	Suvorexant Events	Suvorexant Total	Placebo Events	Placebo Total	Weight	Risk Ratio M-H, Random, 95% CI
**5.2.3 ALL**						
006 all dose	1	127	3	127	3.7%	0.33 [0.04, 3.16]
009 40mg/d	60	522	22	259	36.8%	1.35 [0.85, 2.15]
028 all dose	21	637	21	385	29.6%	0.60 [0.33, 1.09]
029 all dose	29	632	17	387	29.9%	1.04 [0.58, 1.88]
**Subtotal (95% CI)**		**1918**		**1158**	**100.0%**	**0.94 [0.60, 1.47]**
Total events	111		63			
Heterogeneity: Tau² = 0.09; Chi² = 5.34, df = 3 (P = 0.15); I² = 44%						
Test for overall effect: Z = 0.29 (P = 0.77)						
**Total (95% CI)**		**1918**		**1158**	**100.0%**	**0.94 [0.60, 1.47]**
Total events	111		63			
Heterogeneity: Tau² = 0.09; Chi² = 5.34, df = 3 (P = 0.15); I² = 44%						
Test for overall effect: Z = 0.29 (P = 0.77)						
Test for subgroup differences: Not applicable						

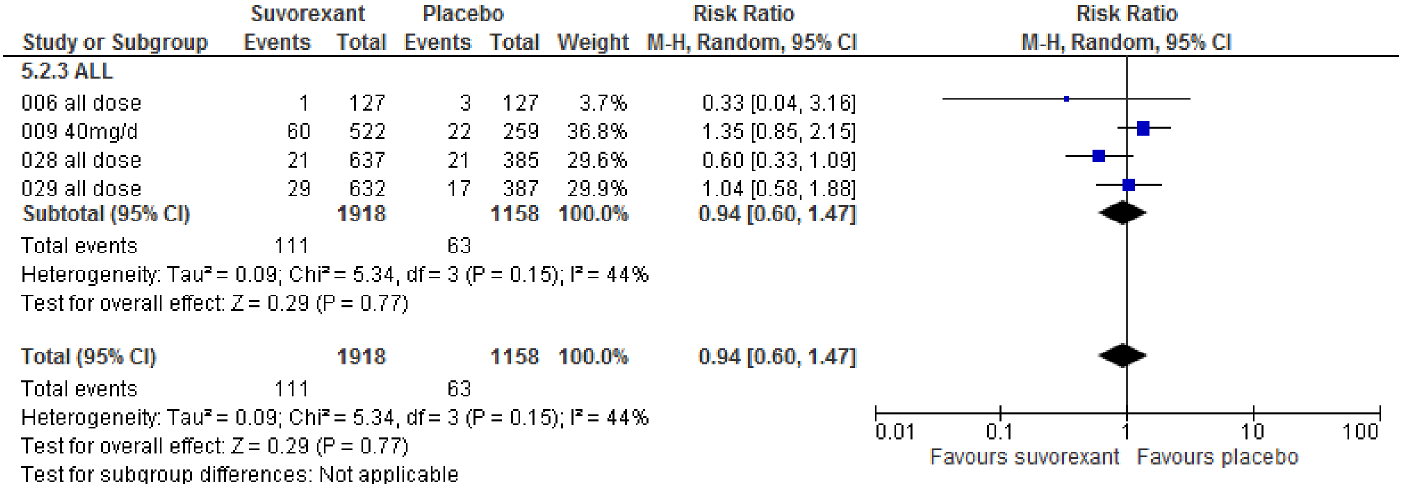

S2-3 Fig. Discontinuation due to inefficacy

Study or Subgroup	Suvorexant Events	Suvorexant Total	Placebo Events	Placebo Total	Weight	Risk Ratio M-H, Random, 95% CI
**5.3.3 ALL**						
006 all dose	1	127	0	127	1.3%	3.00 [0.12, 72.95]
009 40mg/d	44	522	28	259	66.9%	0.78 [0.50, 1.22]
028 all dose	8	637	9	385	15.2%	0.54 [0.21, 1.38]
029 all dose	11	632	8	387	16.6%	0.84 [0.34, 2.07]
**Subtotal (95% CI)**		**1918**		**1158**	**100.0%**	**0.76 [0.53, 1.10]**
Total events	64		45			
Heterogeneity: Tau² = 0.00; Chi² = 1.29, df = 3 (P = 0.73); I² = 0%						
Test for overall effect: Z = 1.46 (P = 0.14)						
**Total (95% CI)**		**1918**		**1158**	**100.0%**	**0.76 [0.53, 1.10]**
Total events	64		45			
Heterogeneity: Tau² = 0.00; Chi² = 1.29, df = 3 (P = 0.73); I² = 0%						
Test for overall effect: Z = 1.46 (P = 0.14)						
Test for subgroup differences: Not applicable						

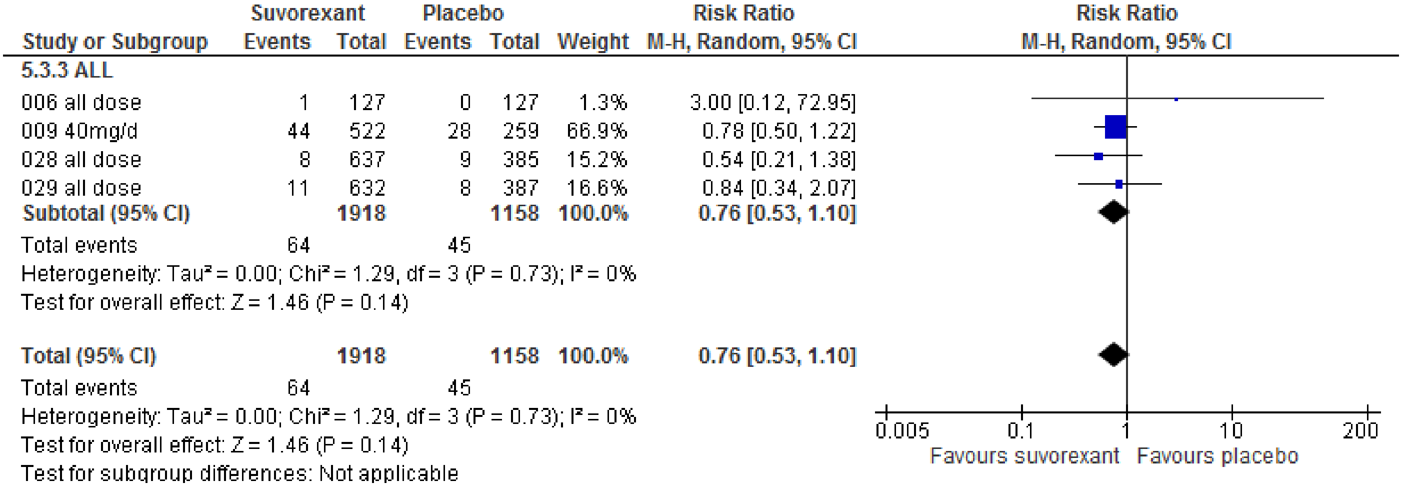

S2-4 Fig. At least one adverse event

Study or Subgroup	Suvorexant Events	Suvorexant Total	Placebo Events	Placebo Total	Weight	Risk Ratio M-H, Random, 95% CI
**5.44.3 ALL**						
009 40mg/d	362	521	164	258	43.0%	1.09 [0.98, 1.22]
028 all dose	324	637	191	384	31.7%	1.02 [0.90, 1.16]
029 all dose	292	626	167	383	25.3%	1.07 [0.93, 1.23]
**Subtotal (95% CI)**		**1784**		**1025**	**100.0%**	**1.06 [0.99, 1.14]**
Total events	978		522			
Heterogeneity: Tau² = 0.00; Chi² = 0.63, df = 2 (P = 0.73); I² = 0%						
Test for overall effect: Z = 1.72 (P = 0.09)						
**Total (95% CI)**		**1784**		**1025**	**100.0%**	**1.06 [0.99, 1.14]**
Total events	978		522			
Heterogeneity: Tau² = 0.00; Chi² = 0.63, df = 2 (P = 0.73); I² = 0%						
Test for overall effect: Z = 1.72 (P = 0.09)						
Test for subgroup differences: Not applicable						

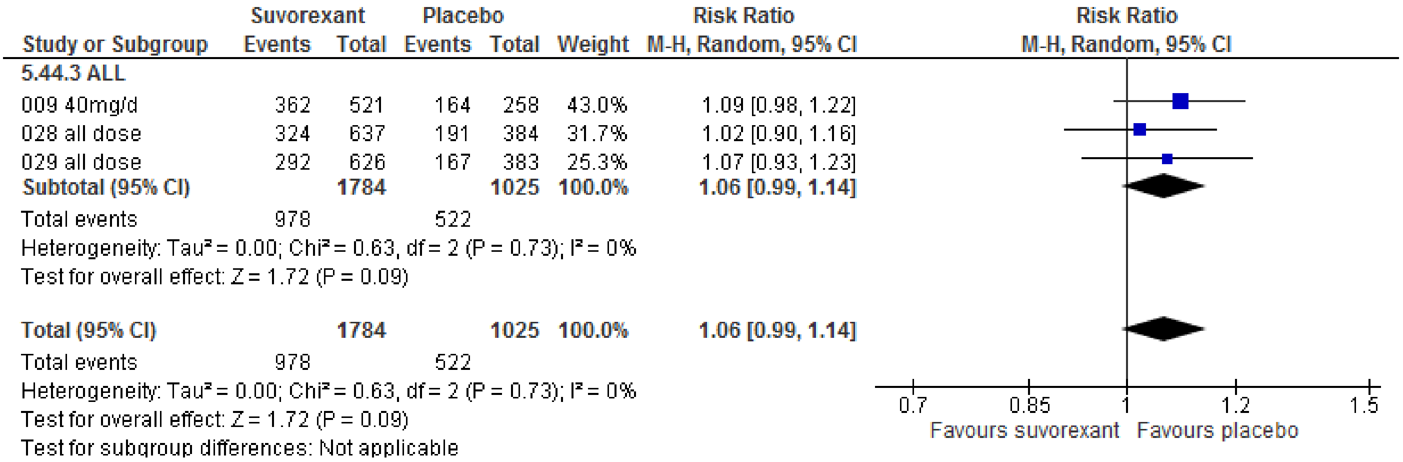

S2-5 Fig. At least one side effect

Study or Subgroup	Suvorexant Events	Suvorexant Total	Placebo Events	Placebo Total	Weight	Risk Ratio M-H, Random, 95% CI
**5.45.1 ALL**						
009 40mg/d	182	521	53	258	34.9%	1.70 [1.30, 2.22]
028 all dose	147	637	53	384	30.2%	1.67 [1.25, 2.23]
029 all dose	144	626	63	383	34.9%	1.40 [1.07, 1.83]
**Subtotal (95% CI)**		**1784**		**1025**	**100.0%**	**1.58 [1.35, 1.85]**
Total events	473		169			
Heterogeneity: Tau² = 0.00; Chi² = 1.24, df = 2 (P = 0.54); I² = 0%						
Test for overall effect: Z = 5.68 (P < 0.00001)						
**Total (95% CI)**		**1784**		**1025**	**100.0%**	**1.58 [1.35, 1.85]**
Total events	473		169			
Heterogeneity: Tau² = 0.00; Chi² = 1.24, df = 2 (P = 0.54); I² = 0%						
Test for overall effect: Z = 5.68 (P < 0.00001)						
Test for subgroup differences: Not applicable						

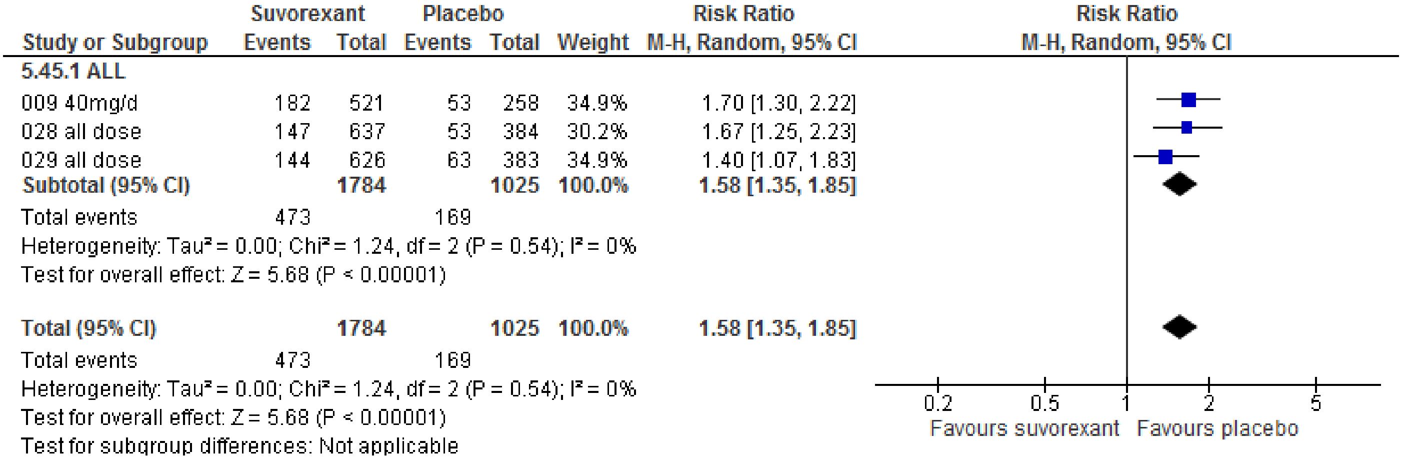

S2-6 Fig. Serious adverse event

Study or Subgroup	Suvorexant Events	Suvorexant Total	Placebo Events	Placebo Total	Weight	Risk Ratio M-H, Random, 95% CI
**5.46.1 ALL**						
009 40mg/d	27	521	17	258	44.2%	0.79 [0.44, 1.42]
028 all dose	1	637	11	384	20.8%	0.05 [0.01, 0.42]
029 all dose	8	626	5	383	35.0%	0.98 [0.32, 2.97]
**Subtotal (95% CI)**		**1784**		**1025**	**100.0%**	**0.49 [0.14, 1.66]**
Total events	36		33			
Heterogeneity: Tau² = 0.80; Chi² = 7.01, df = 2 (P = 0.03); I² = 71%						
Test for overall effect: Z = 1.15 (P = 0.25)						
**Total (95% CI)**		**1784**		**1025**	**100.0%**	**0.49 [0.14, 1.66]**
Total events	36		33			
Heterogeneity: Tau² = 0.80; Chi² = 7.01, df = 2 (P = 0.03); I² = 71%						
Test for overall effect: Z = 1.15 (P = 0.25)						
Test for subgroup differences: Not applicable						

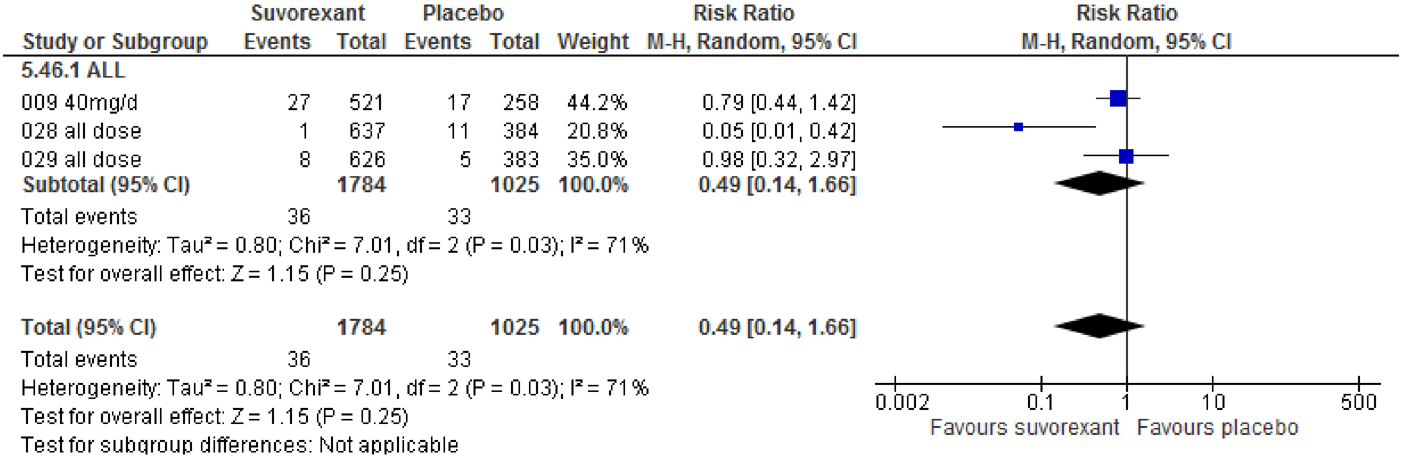

S2-7 Fig. Somnolence

Study or Subgroup	Suvorexant Events	Suvorexant Total	Placebo Events	Placebo Total	Weight	Risk Ratio M-H, Random, 95% CI
**5.48.1 ALL**						
009 40mg/d	69	521	7	258	23.6%	4.88 [2.28, 10.47]
028 all dose	54	637	13	384	39.1%	2.50 [1.39, 4.53]
029 all dose	60	626	12	383	37.3%	3.06 [1.67, 5.61]
**Subtotal (95% CI)**		**1784**		**1025**	**100.0%**	**3.16 [2.18, 4.57]**
Total events	183		32			
Heterogeneity: Tau² = 0.00; Chi² = 1.88, df = 2 (P = 0.39); I² = 0%						
Test for overall effect: Z = 6.08 (P < 0.00001)						
**Total (95% CI)**		**1784**		**1025**	**100.0%**	**3.16 [2.18, 4.57]**
Total events	183		32			
Heterogeneity: Tau² = 0.00; Chi² = 1.88, df = 2 (P = 0.39); I² = 0%						
Test for overall effect: Z = 6.08 (P < 0.00001)						
Test for subgroup differences: Not applicable						

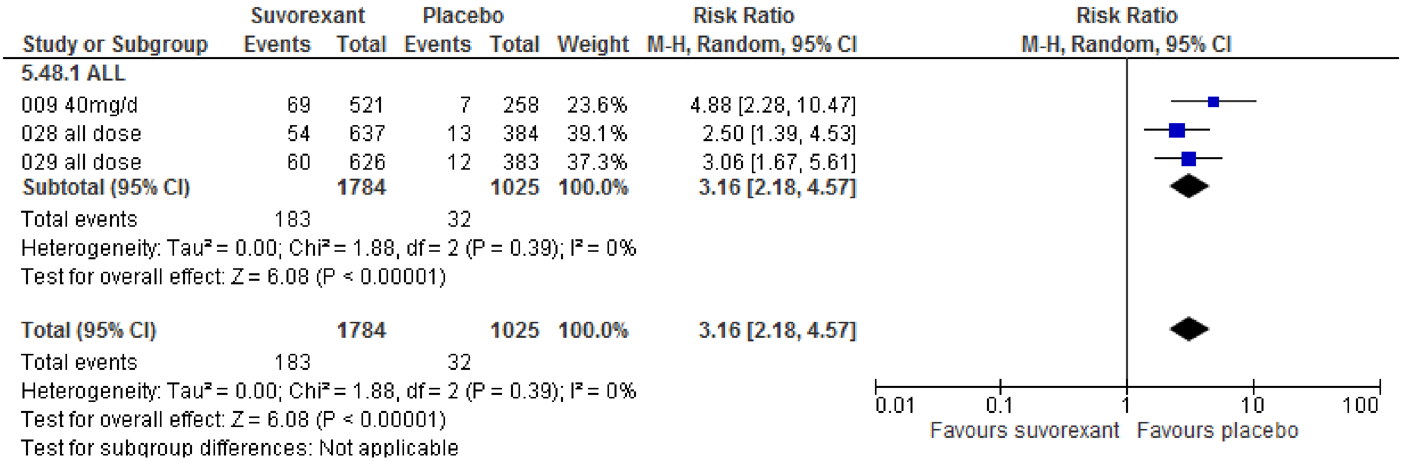

S2-8 Fig. Excessive daytime sleepiness/sedation

Study or Subgroup	Suvorexant Events	Suvorexant Total	Placebo Events	Placebo Total	Weight	Risk Ratio M-H, Random, 95% CI
**5.49.1 ALL**						
009 40mg/d	13	521	2	258	58.1%	3.22 [0.73, 14.16]
028 all dose	4	637	1	384	26.6%	2.41 [0.27, 21.49]
029 all dose	5	626	0	383	15.2%	6.74 [0.37, 121.49]
**Subtotal (95% CI)**		**1784**		**1025**	**100.0%**	**3.34 [1.08, 10.32]**
Total events	22		3			
Heterogeneity: Tau² = 0.00; Chi² = 0.32, df = 2 (P = 0.85); I² = 0%						
Test for overall effect: Z = 2.09 (P = 0.04)						
**Total (95% CI)**		**1784**		**1025**	**100.0%**	**3.34 [1.08, 10.32]**
Total events	22		3			
Heterogeneity: Tau² = 0.00; Chi² = 0.32, df = 2 (P = 0.85); I² = 0%						
Test for overall effect: Z = 2.09 (P = 0.04)						
Test for subgroup differences: Not applicable						

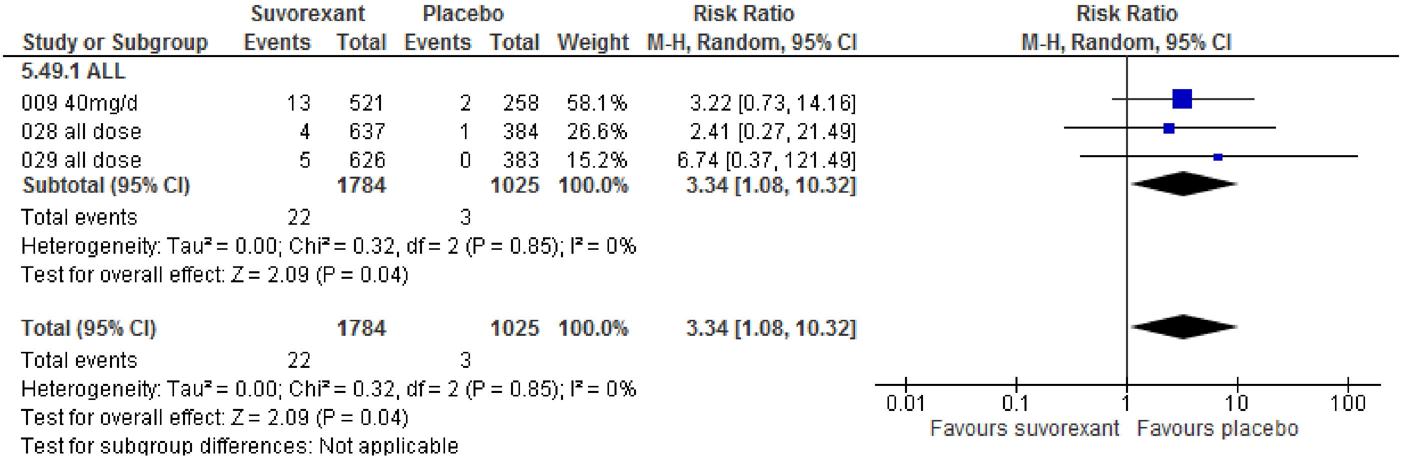

S2-9 Fig. Fatigue

Study or Subgroup	Suvorexant Events	Suvorexant Total	Placebo Events	Placebo Total	Weight	Risk Ratio M-H, Random, 95% CI
**5.50.1 ALL**						
009 40mg/d	34	521	5	258	33.0%	3.37 [1.33, 8.51]
028 all dose	14	637	3	384	21.9%	2.81 [0.81, 9.73]
029 all dose	23	626	11	383	45.1%	1.28 [0.63, 2.59]
**Subtotal (95% CI)**		**1784**		**1025**	**100.0%**	**2.09 [1.08, 4.06]**
Total events	71		19			
Heterogeneity: Tau² = 0.12; Chi² = 3.10, df = 2 (P = 0.21); I² = 35%						
Test for overall effect: Z = 2.18 (P = 0.03)						
**Total (95% CI)**		**1784**		**1025**	**100.0%**	**2.09 [1.08, 4.06]**
Total events	71		19			
Heterogeneity: Tau² = 0.12; Chi² = 3.10, df = 2 (P = 0.21); I² = 35%						
Test for overall effect: Z = 2.18 (P = 0.03)						
Test for subgroup differences: Not applicable						

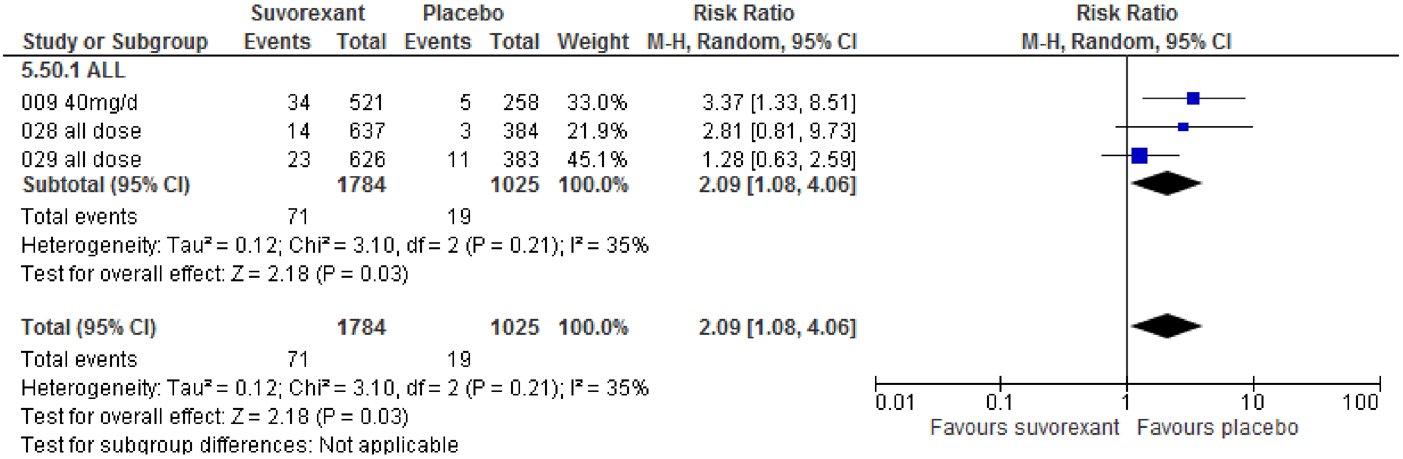

S2-10 Fig. Cataplexy

Study or Subgroup	Suvorexant Events	Suvorexant Total	Placebo Events	Placebo Total	Weight	Risk Ratio M-H, Random, 95% CI
**5.51.1 ALL**						
009 40mg/d	0	521	0	258		Not estimable
028 all dose	0	637	0	384		Not estimable
029 all dose	0	626	0	383		Not estimable
**Subtotal (95% CI)**		**1784**		**1025**		**Not estimable**
Total events	0		0			
Heterogeneity: Not applicable						
Test for overall effect: Not applicable						
**Total (95% CI)**		**1784**		**1025**		**Not estimable**
Total events	0		0			
Heterogeneity: Not applicable						
Test for overall effect: Not applicable						
Test for subgroup differences: Not applicable						

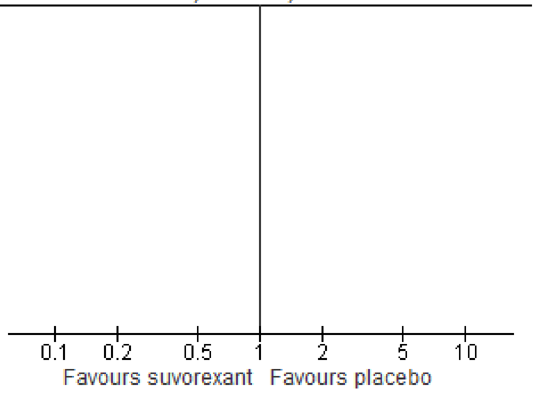

S2-11 Fig. Sleep paralysis

Study or Subgroup	Suvorexant Events	Suvorexant Total	Placebo Events	Placebo Total	Weight	Risk Ratio M-H, Random, 95% CI
**5.52.1 ALL**						
009 40mg/d	2	521	0	258	33.9%	2.48 [0.12, 51.49]
028 all dose	3	637	0	384	35.6%	4.22 [0.22, 81.56]
029 all dose	1	626	0	383	30.5%	1.84 [0.08, 44.99]
**Subtotal (95% CI)**		**1784**		**1025**	**100.0%**	**2.74 [0.47, 16.00]**
Total events	6		0			
Heterogeneity: Tau² = 0.00; Chi² = 0.15, df = 2 (P = 0.93); I² = 0%						
Test for overall effect: Z = 1.12 (P = 0.26)						
**Total (95% CI)**		**1784**		**1025**	**100.0%**	**2.74 [0.47, 16.00]**
Total events	6		0			
Heterogeneity: Tau² = 0.00; Chi² = 0.15, df = 2 (P = 0.93); I² = 0%						
Test for overall effect: Z = 1.12 (P = 0.26)						
Test for subgroup differences: Not applicable						

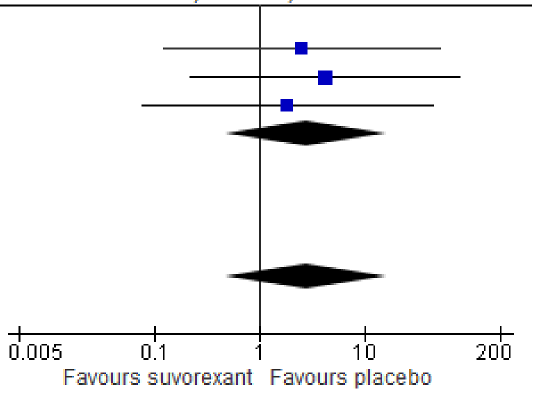

S2-12 Fig. Complex sleep-related behaviours

Study or Subgroup	Suvorexant Events	Suvorexant Total	Placebo Events	Placebo Total	Weight	Risk Ratio M-H, Random, 95% CI
**5.53.1 ALL**						
009 40mg/d	1	521	0	258	50.0%	1.49 [0.06, 36.41]
028 all dose	0	637	0	384		Not estimable
029 all dose	1	626	0	383	50.0%	1.84 [0.08, 44.99]
**Subtotal (95% CI)**		**1784**		**1025**	**100.0%**	**1.65 [0.17, 15.86]**
Total events	2		0			
Heterogeneity: Tau² = 0.00; Chi² = 0.01, df = 1 (P = 0.93); I² = 0%						
Test for overall effect: Z = 0.44 (P = 0.66)						
**Total (95% CI)**		**1784**		**1025**	**100.0%**	**1.65 [0.17, 15.86]**
Total events	2		0			
Heterogeneity: Tau² = 0.00; Chi² = 0.01, df = 1 (P = 0.93); I² = 0%						
Test for overall effect: Z = 0.44 (P = 0.66)						
Test for subgroup differences: Not applicable						

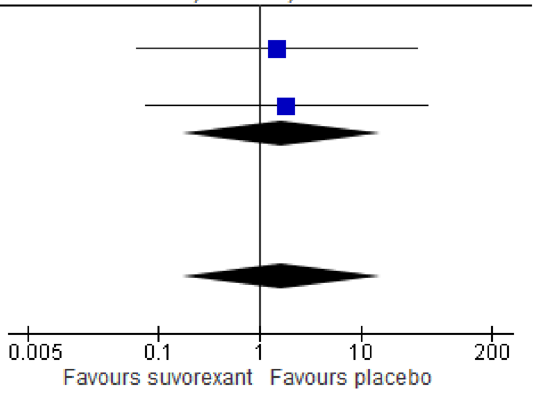

S2-13 Fig. Hypnagogic hallucination

Study or Subgroup	Suvorexant Events	Suvorexant Total	Placebo Events	Placebo Total	Weight	Risk Ratio M-H, Random, 95% CI
**5.54.1 ALL**						
009 40mg/d	3	521	0	258	36.9%	3.47 [0.18, 66.99]
028 all dose	1	637	0	384	31.6%	1.81 [0.07, 44.33]
029 all dose	1	626	0	383	31.6%	1.84 [0.08, 44.99]
**Subtotal (95% CI)**		**1784**		**1025**	**100.0%**	**2.31 [0.38, 13.95]**
Total events	5		0			
Heterogeneity: Tau² = 0.00; Chi² = 0.12, df = 2 (P = 0.94); I² = 0%						
Test for overall effect: Z = 0.91 (P = 0.36)						
**Total (95% CI)**		**1784**		**1025**	**100.0%**	**2.31 [0.38, 13.95]**
Total events	5		0			
Heterogeneity: Tau² = 0.00; Chi² = 0.12, df = 2 (P = 0.94); I² = 0%						
Test for overall effect: Z = 0.91 (P = 0.36)						
Test for subgroup differences: Not applicable						

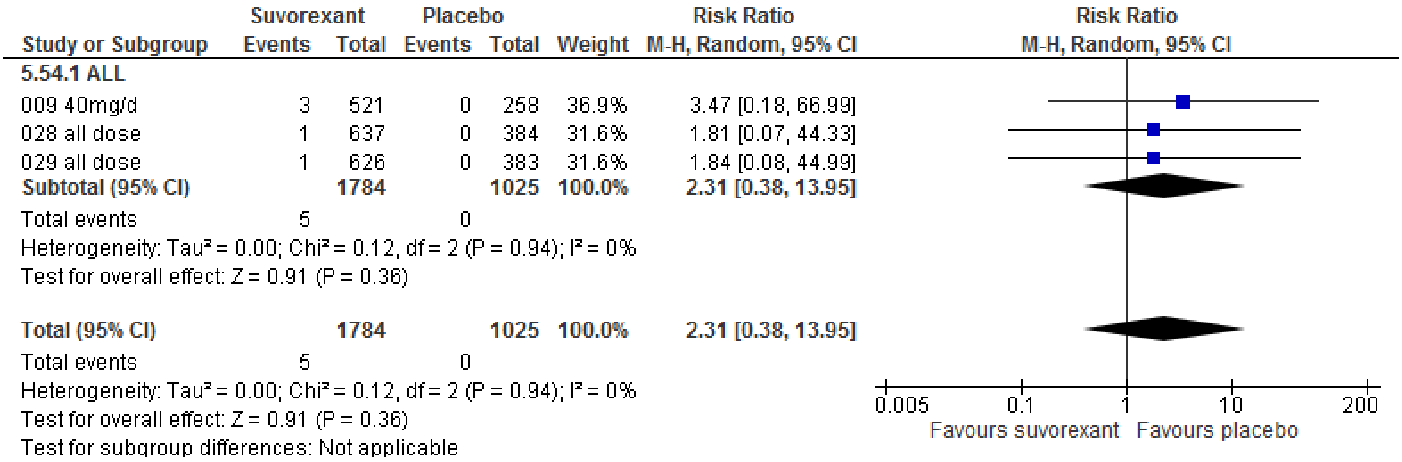

S2-14 Fig. Hypnopompic hallucination

Study or Subgroup	Suvorexant Events	Suvorexant Total	Placebo Events	Placebo Total	Weight	Risk Ratio M-H, Random, 95% CI
**5.55.1 ALL**						
009 40mg/d	1	521	0	258	50.0%	1.49 [0.06, 36.41]
028 all dose	0	637	0	384		Not estimable
029 all dose	1	626	0	383	50.0%	1.84 [0.08, 44.99]
**Subtotal (95% CI)**		**1784**		**1025**	**100.0%**	**1.65 [0.17, 15.86]**
Total events	2		0			
Heterogeneity: Tau² = 0.00; Chi² = 0.01, df = 1 (P = 0.93); I² = 0%						
Test for overall effect: Z = 0.44 (P = 0.66)						
**Total (95% CI)**		**1784**		**1025**	**100.0%**	**1.65 [0.17, 15.86]**
Total events	2		0			
Heterogeneity: Tau² = 0.00; Chi² = 0.01, df = 1 (P = 0.93); I² = 0%						
Test for overall effect: Z = 0.44 (P = 0.66)						
Test for subgroup differences: Not applicable						

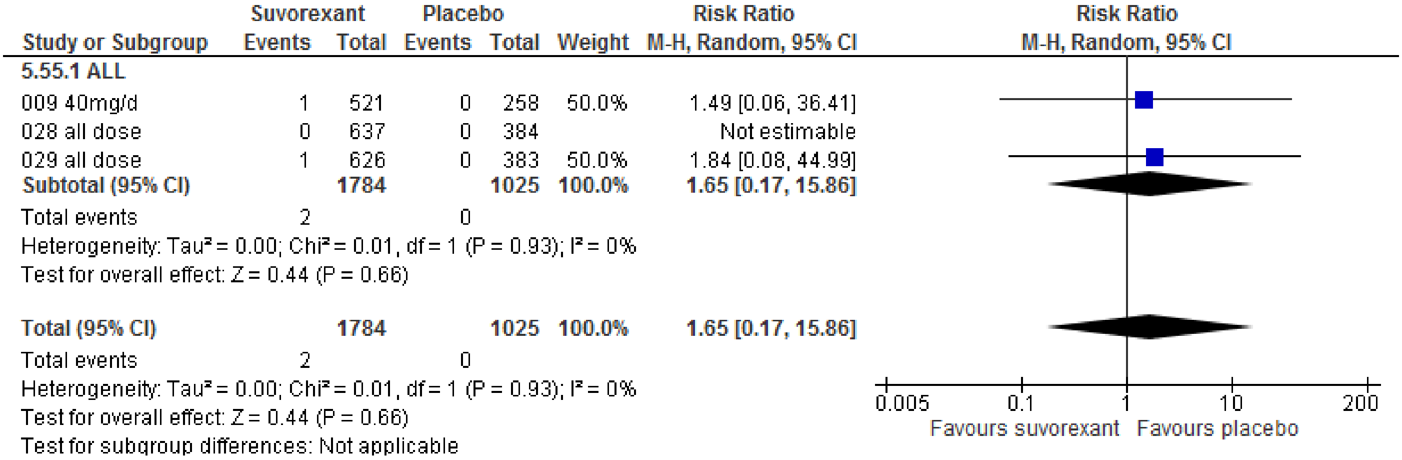

S2-15 Fig. Abnormal dream

Study or Subgroup	Suvorexant Events	Suvorexant Total	Placebo Events	Placebo Total	Weight	Risk Ratio M-H, Random, 95% CI
**5.56.1 ALL**						
028 all dose	12	637	3	384	58.4%	2.41 [0.68, 8.49]
029 all dose	12	626	2	383	41.6%	3.67 [0.83, 16.31]
**Subtotal (95% CI)**		**1263**		**767**	**100.0%**	**2.87 [1.10, 7.52]**
Total events	24		5			
Heterogeneity: Tau² = 0.00; Chi² = 0.18, df = 1 (P = 0.67); I² = 0%						
Test for overall effect: Z = 2.15 (P = 0.03)						
**Total (95% CI)**		**1263**		**767**	**100.0%**	**2.87 [1.10, 7.52]**
Total events	24		5			
Heterogeneity: Tau² = 0.00; Chi² = 0.18, df = 1 (P = 0.67); I² = 0%						
Test for overall effect: Z = 2.15 (P = 0.03)						
Test for subgroup differences: Not applicable						

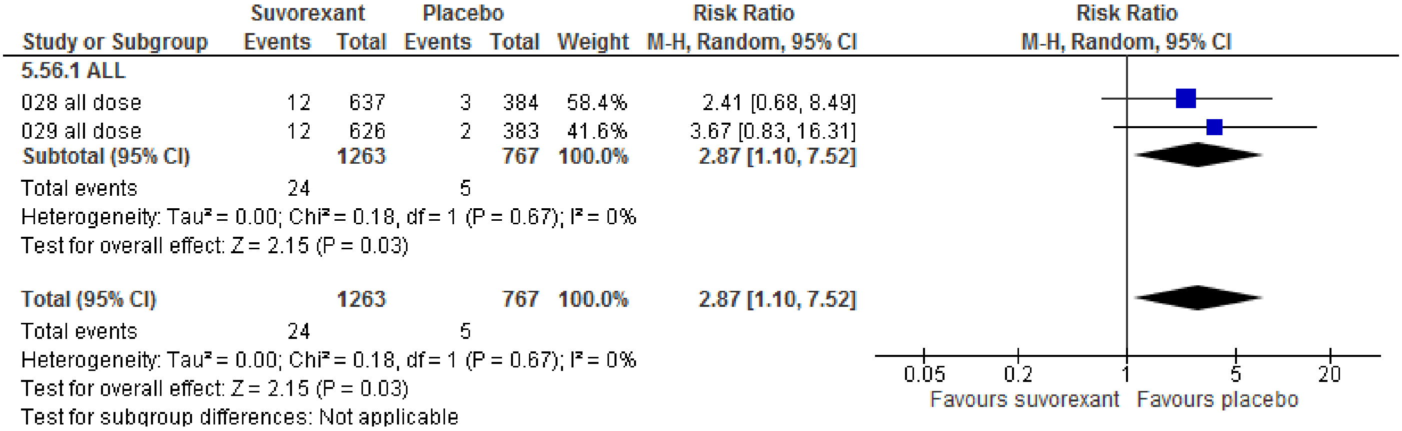

S2-16 Fig. Suicidal ideation

Study or Subgroup	Suvorexant Events	Suvorexant Total	Placebo Events	Placebo Total	Weight	Risk Ratio M-H, Random, 95% CI
**5.57.1 ALL**						
009 40mg/d	4	521	0	258	35.2%	4.47 [0.24, 82.63]
028 all dose	0	637	1	384	30.4%	0.20 [0.01, 4.93]
029 all dose	3	626	0	383	34.4%	4.29 [0.22, 82.77]
**Subtotal (95% CI)**		**1784**		**1025**	**100.0%**	**1.72 [0.24, 12.13]**
Total events	7		1			
Heterogeneity: Tau² = 0.61; Chi² = 2.51, df = 2 (P = 0.28); I² = 20%						
Test for overall effect: Z = 0.54 (P = 0.59)						
**Total (95% CI)**		**1784**		**1025**	**100.0%**	**1.72 [0.24, 12.13]**
Total events	7		1			
Heterogeneity: Tau² = 0.61; Chi² = 2.51, df = 2 (P = 0.28); I² = 20%						
Test for overall effect: Z = 0.54 (P = 0.59)						
Test for subgroup differences: Not applicable						

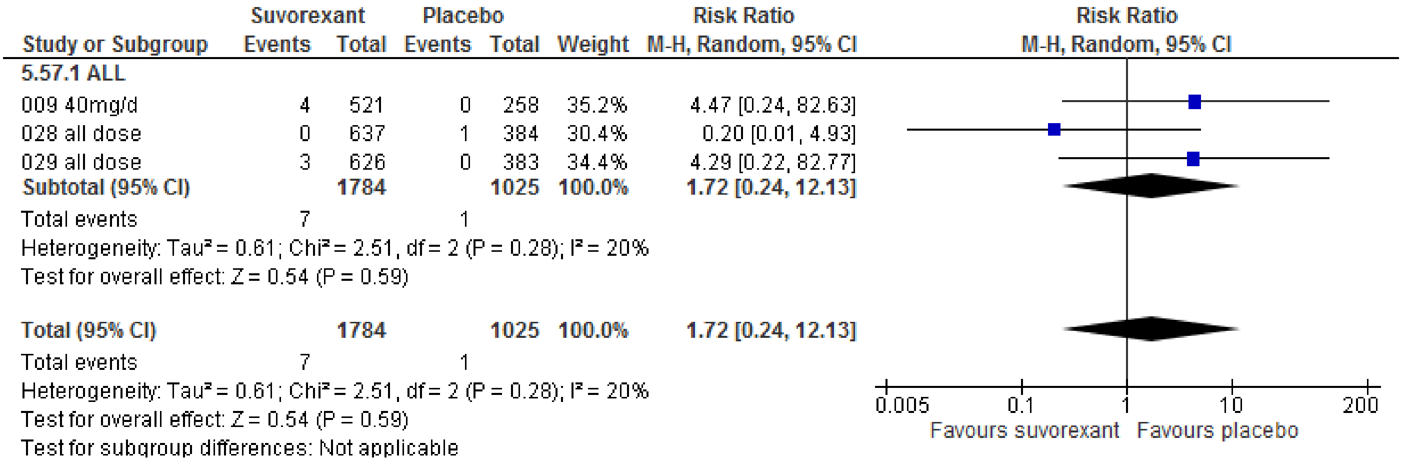

S2-17 Fig. Events suggesting drug-abuse potential

Study or Subgroup	Suvorexant Events	Suvorexant Total	Placebo Events	Placebo Total	Weight	Risk Ratio M-H, Random, 95% CI
**5.58.1 ALL**						
009 40mg/d	18	521	10	258	35.6%	0.89 [0.42, 1.90]
028 all dose	19	637	10	384	35.9%	1.15 [0.54, 2.44]
029 all dose	15	626	8	383	28.5%	1.15 [0.49, 2.68]
**Subtotal (95% CI)**		**1784**		**1025**	**100.0%**	**1.05 [0.67, 1.65]**
Total events	52		28			
Heterogeneity: Tau² = 0.00; Chi² = 0.27, df = 2 (P = 0.87); I² = 0%						
Test for overall effect: Z = 0.20 (P = 0.84)						
**Total (95% CI)**		**1784**		**1025**	**100.0%**	**1.05 [0.67, 1.65]**
Total events	52		28			
Heterogeneity: Tau² = 0.00; Chi² = 0.27, df = 2 (P = 0.87); I² = 0%						
Test for overall effect: Z = 0.20 (P = 0.84)						
Test for subgroup differences: Not applicable						

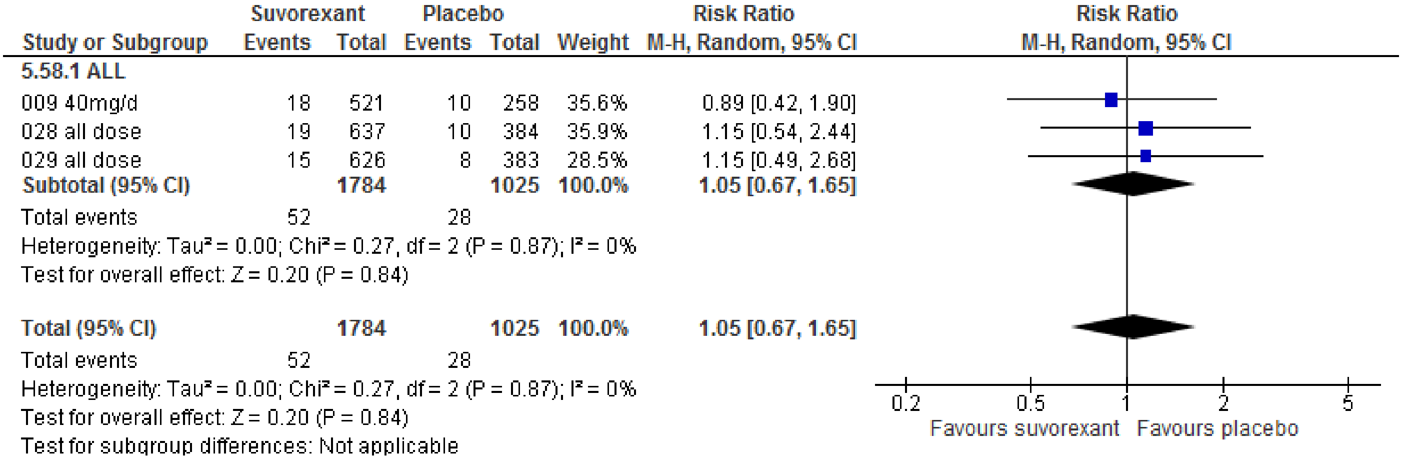

S2-18 Fig. Fall

Study or Subgroup	Suvorexant Events	Suvorexant Total	Placebo Events	Placebo Total	Weight	Risk Ratio M-H, Random, 95% CI
**5.60.1 ALL**						
009 40mg/d	12	521	8	258	54.7%	0.74 [0.31, 1.79]
028 all dose	5	637	5	384	28.0%	0.60 [0.18, 2.07]
029 all dose	7	626	2	383	17.3%	2.14 [0.45, 10.25]
**Subtotal (95% CI)**		**1784**		**1025**	**100.0%**	**0.84 [0.44, 1.62]**
Total events	24		15			
Heterogeneity: Tau² = 0.00; Chi² = 1.74, df = 2 (P = 0.42); I² = 0%						
Test for overall effect: Z = 0.52 (P = 0.60)						
**Total (95% CI)**		**1784**		**1025**	**100.0%**	**0.84 [0.44, 1.62]**
Total events	24		15			
Heterogeneity: Tau² = 0.00; Chi² = 1.74, df = 2 (P = 0.42); I² = 0%						
Test for overall effect: Z = 0.52 (P = 0.60)						
Test for subgroup differences: Not applicable						

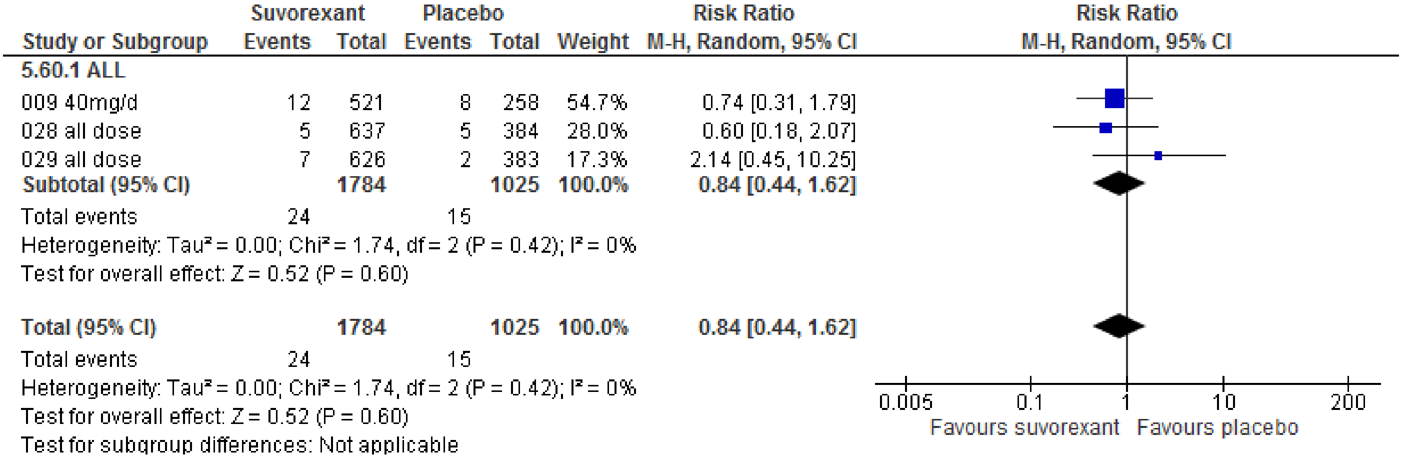

S2-19 Fig. Headache

Study or Subgroup	Suvorexant Events	Suvorexant Total	Placebo Events	Placebo Total	Weight	Risk Ratio M-H, Random, 95% CI
**5.63.1 ALL**						
009 40mg/d	43	521	22	258	33.1%	0.97 [0.59, 1.58]
028 all dose	43	637	23	384	33.3%	1.13 [0.69, 1.84]
029 all dose	48	626	22	383	33.6%	1.33 [0.82, 2.18]
**Subtotal (95% CI)**		**1784**		**1025**	**100.0%**	**1.13 [0.85, 1.51]**
Total events	134		67			
Heterogeneity: Tau² = 0.00; Chi² = 0.83, df = 2 (P = 0.66); I² = 0%						
Test for overall effect: Z = 0.87 (P = 0.38)						
**Total (95% CI)**		**1784**		**1025**	**100.0%**	**1.13 [0.85, 1.51]**
Total events	134		67			
Heterogeneity: Tau² = 0.00; Chi² = 0.83, df = 2 (P = 0.66); I² = 0%						
Test for overall effect: Z = 0.87 (P = 0.38)						
Test for subgroup differences: Not applicable						

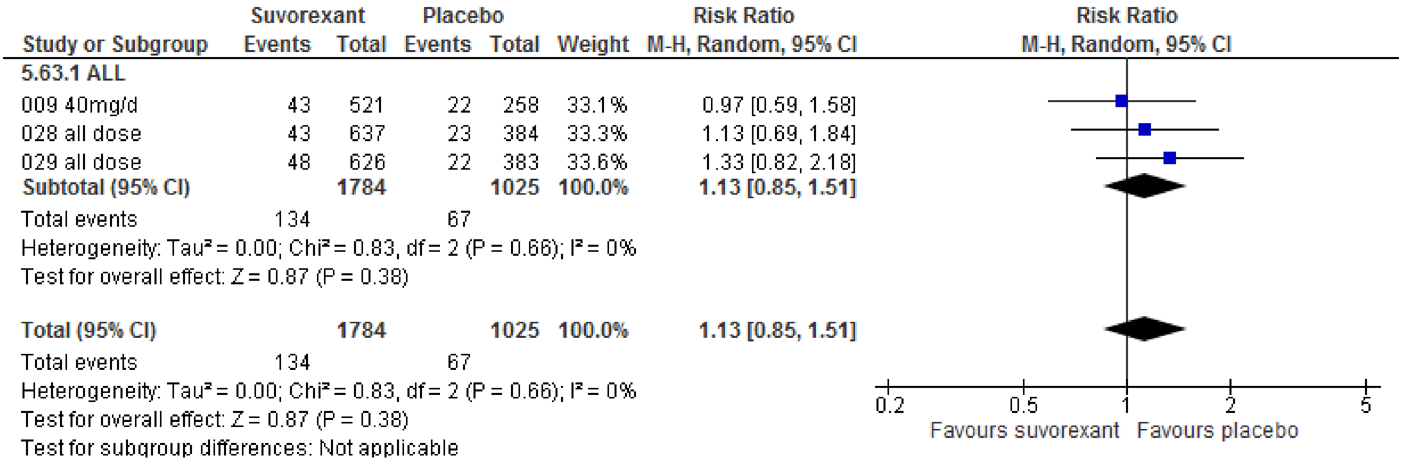

S2-20 Fig. Dizziness

Study or Subgroup	Suvorexant Events	Suvorexant Total	Placebo Events	Placebo Total	Weight	Risk Ratio M-H, Random, 95% CI
**5.65.1 ALL**						
009 40mg/d	24	521	16	258	45.7%	0.74 [0.40, 1.37]
028 all dose	17	637	11	384	30.9%	0.93 [0.44, 1.97]
029 all dose	14	626	8	383	23.4%	1.07 [0.45, 2.53]
**Subtotal (95% CI)**		**1784**		**1025**	**100.0%**	**0.87 [0.57, 1.31]**
Total events	55		35			
Heterogeneity: Tau² = 0.00; Chi² = 0.51, df = 2 (P = 0.77); I² = 0%						
Test for overall effect: Z = 0.67 (P = 0.50)						
**Total (95% CI)**		**1784**		**1025**	**100.0%**	**0.87 [0.57, 1.31]**
Total events	55		35			
Heterogeneity: Tau² = 0.00; Chi² = 0.51, df = 2 (P = 0.77); I² = 0%						
Test for overall effect: Z = 0.67 (P = 0.50)						
Test for subgroup differences: Not applicable						

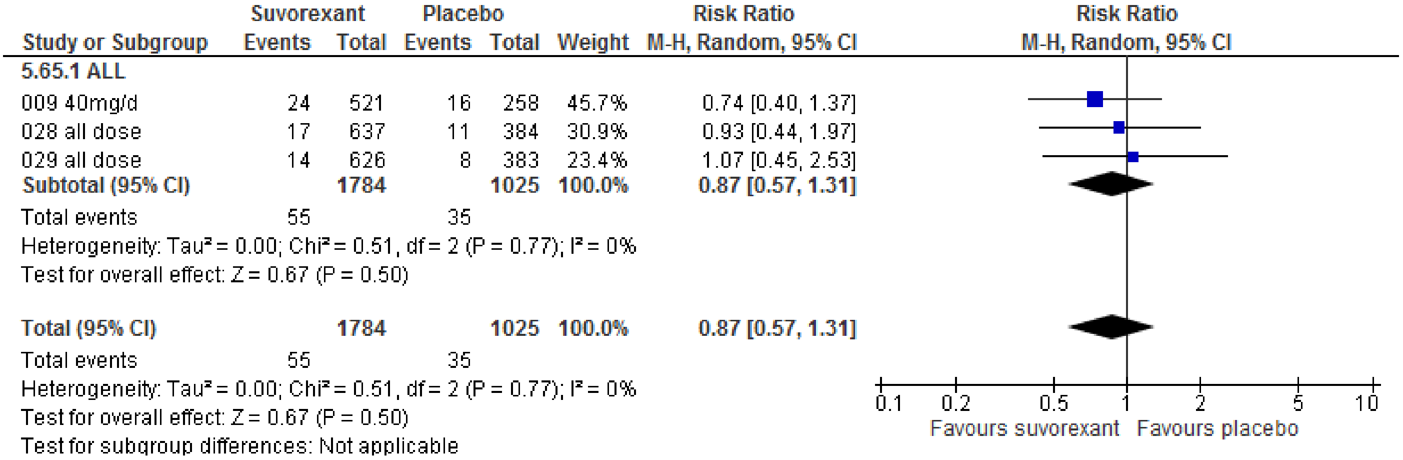

S2-21 Fig. Back pain

Study or Subgroup	Suvorexant Events	Suvorexant Total	Placebo Events	Placebo Total	Weight	Risk Ratio M-H, Random, 95% CI
**5.64.1 ALL**						
009 40mg/d	0	521	1	258	4.0%	0.17 [0.01, 4.05]
028 all dose	6	637	8	384	36.7%	0.45 [0.16, 1.29]
029 all dose	11	626	11	383	59.4%	0.61 [0.27, 1.40]
**Subtotal (95% CI)**		**1784**		**1025**	**100.0%**	**0.52 [0.28, 0.98]**
Total events	17		20			
Heterogeneity: Tau² = 0.00; Chi² = 0.71, df = 2 (P = 0.70); I² = 0%						
Test for overall effect: Z = 2.01 (P = 0.04)						
**Total (95% CI)**		**1784**		**1025**	**100.0%**	**0.52 [0.28, 0.98]**
Total events	17		20			
Heterogeneity: Tau² = 0.00; Chi² = 0.71, df = 2 (P = 0.70); I² = 0%						
Test for overall effect: Z = 2.01 (P = 0.04)						
Test for subgroup differences: Not applicable						

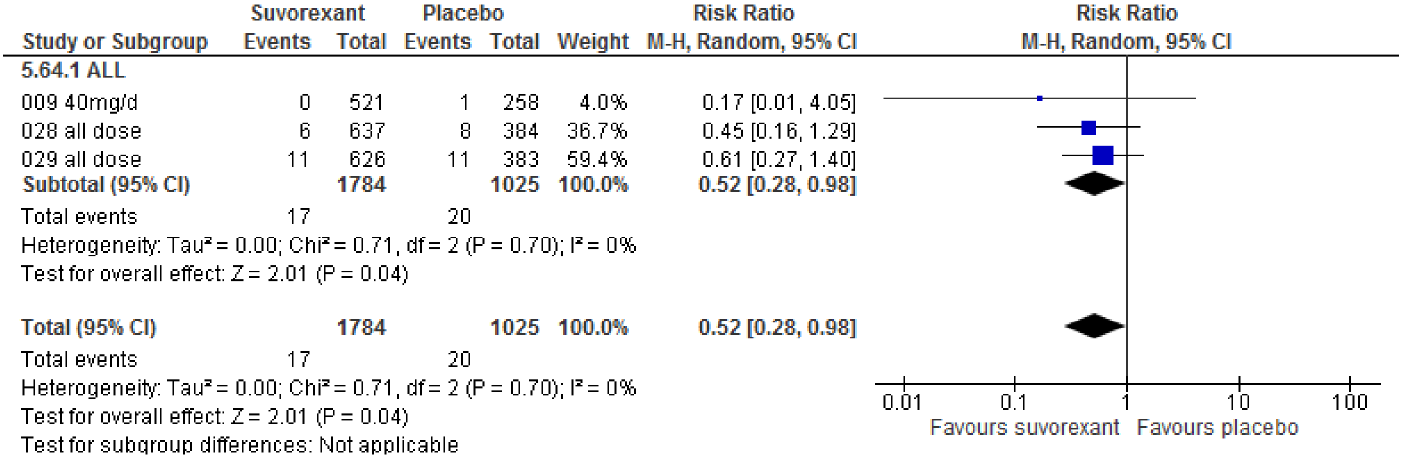

S2-22 Fig. Dry mouth

Study or Subgroup	Suvorexant Events	Suvorexant Total	Placebo Events	Placebo Total	Weight	Risk Ratio M-H, Random, 95% CI
**5.66.1 ALL**						
009 40mg/d	26	521	4	258	32.5%	3.22 [1.14, 9.13]
028 all dose	10	637	4	384	26.6%	1.51 [0.48, 4.77]
029 all dose	16	626	6	383	40.9%	1.63 [0.64, 4.13]
**Subtotal (95% CI)**		**1784**		**1025**	**100.0%**	**1.99 [1.10, 3.61]**
Total events	52		14			
Heterogeneity: Tau² = 0.00; Chi² = 1.23, df = 2 (P = 0.54); I² = 0%						
Test for overall effect: Z = 2.27 (P = 0.02)						
**Total (95% CI)**		**1784**		**1025**	**100.0%**	**1.99 [1.10, 3.61]**
Total events	52		14			
Heterogeneity: Tau² = 0.00; Chi² = 1.23, df = 2 (P = 0.54); I² = 0%						
Test for overall effect: Z = 2.27 (P = 0.02)						
Test for subgroup differences: Not applicable						

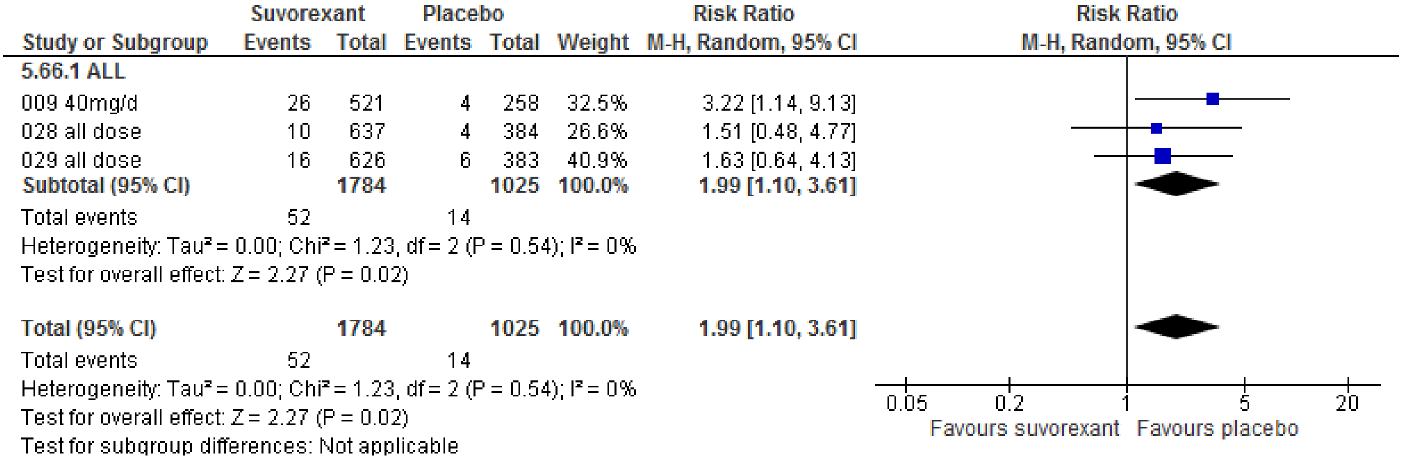

S2-23 Fig. Nasopharyngitis

Study or Subgroup	Suvorexant Events	Suvorexant Total	Placebo Events	Placebo Total	Weight	Risk Ratio M-H, Random, 95% CI
**5.67.1 ALL**						
009 40mg/d	42	521	20	258	32.6%	1.04 [0.62, 1.73]
028 all dose	50	637	34	384	49.0%	0.89 [0.58, 1.35]
029 all dose	21	626	13	383	18.4%	0.99 [0.50, 1.95]
**Subtotal (95% CI)**		**1784**		**1025**	**100.0%**	**0.95 [0.71, 1.28]**
Total events	113		67			
Heterogeneity: Tau² = 0.00; Chi² = 0.24, df = 2 (P = 0.89); I² = 0%						
Test for overall effect: Z = 0.33 (P = 0.74)						
**Total (95% CI)**		**1784**		**1025**	**100.0%**	**0.95 [0.71, 1.28]**
Total events	113		67			
Heterogeneity: Tau² = 0.00; Chi² = 0.24, df = 2 (P = 0.89); I² = 0%						
Test for overall effect: Z = 0.33 (P = 0.74)						
Test for subgroup differences: Not applicable						

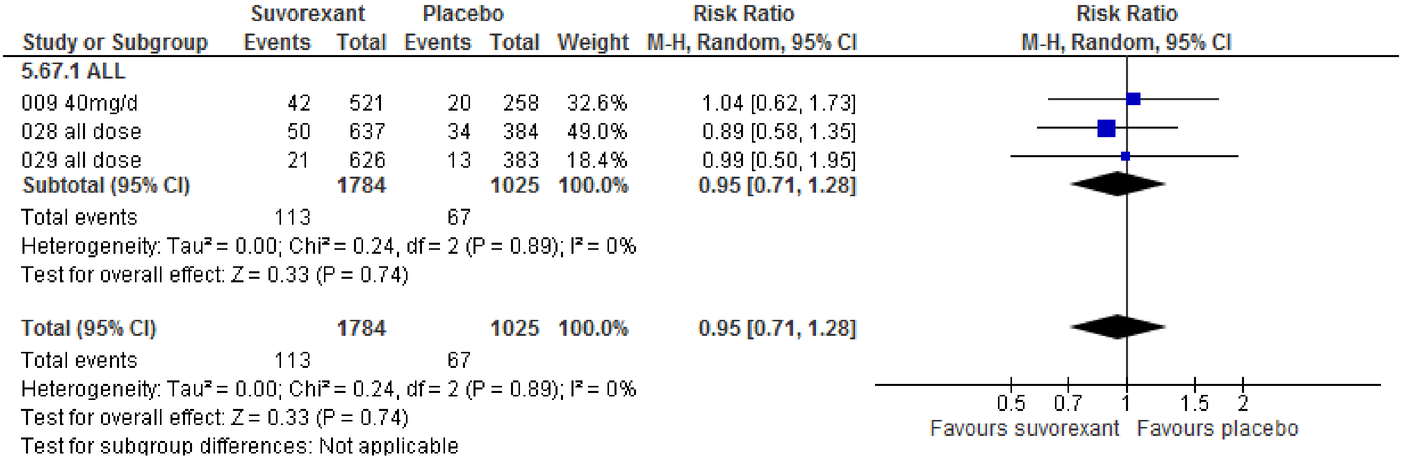

S2-24 Fig. Motor vehicle accidents/violations

Study or Subgroup	Suvorexant Events	Suvorexant Total	Placebo Events	Placebo Total	Weight	Risk Ratio M-H, Random, 95% CI
**5.61.1 ALL**						
009 40mg/d	2	521	0	258	6.8%	2.48 [0.12, 51.49]
028 all dose	11	637	9	384	59.8%	0.74 [0.31, 1.76]
029 all dose	11	626	3	383	33.4%	2.24 [0.63, 7.99]
**Subtotal (95% CI)**		**1784**		**1025**	**100.0%**	**1.16 [0.52, 2.60]**
Total events	24		12			
Heterogeneity: Tau² = 0.08; Chi² = 2.33, df = 2 (P = 0.31); I² = 14%						
Test for overall effect: Z = 0.36 (P = 0.72)						
**Total (95% CI)**		**1784**		**1025**	**100.0%**	**1.16 [0.52, 2.60]**
Total events	24		12			
Heterogeneity: Tau² = 0.08; Chi² = 2.33, df = 2 (P = 0.31); I² = 14%						
Test for overall effect: Z = 0.36 (P = 0.72)						
Test for subgroup differences: Not applicable						

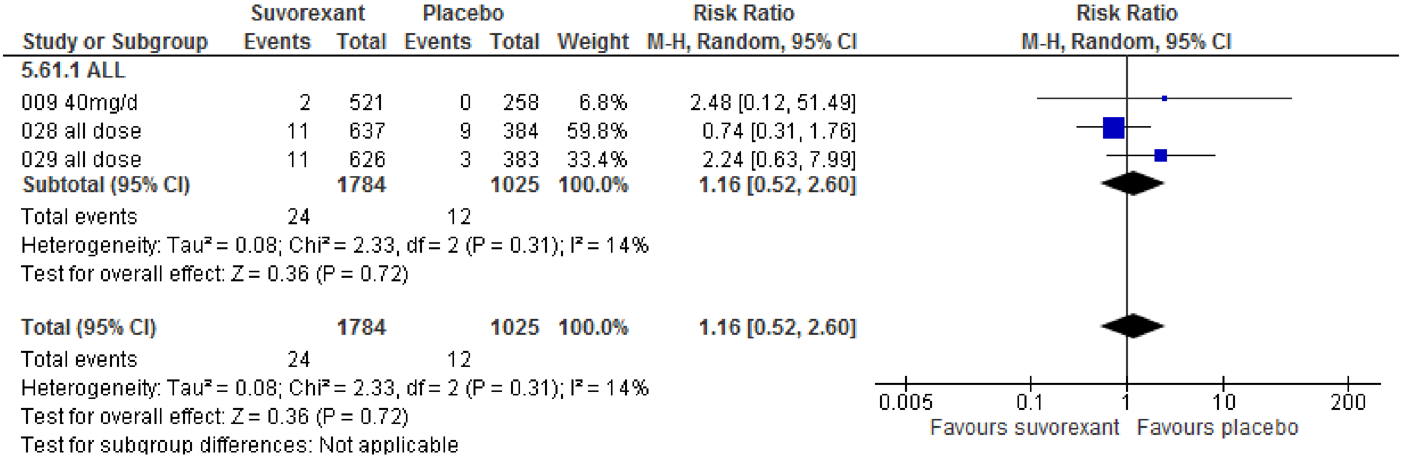

Supplement: S2 Fig — (PDF) [file pone.0136910.s003.pdf]
