# Supplementary material for: Suvorexant for Primary Insomnia: A Systematic Review and Meta-Analysis of Randomized Placebo-Controlled Trials
Source: PLoS One. 2015 Aug 28;10(8):e0136910. doi: 10.1371/journal.pone.0136910 (PMC4552781; doi:10.1371/journal.pone.0136910)

S3 Fig. Forest plots of study run-out phase outcomes.

S3-1 Fig. Discontinuation due to all cause

Study or Subgroup	SUV-->SUV Events	SUV-->SUV Total	SUV-->PLA Events	SUV-->PLA Total	Weight	Risk Ratio M-H, Random, 95% CI
**6.1.3 ALL**						
009 40mg/d	4	156	5	166	50.7%	0.85 [0.23, 3.11]
028 all dose	2	261	2	274	22.4%	1.05 [0.15, 7.40]
029 all dose	2	270	3	279	26.9%	0.69 [0.12, 4.09]
**Subtotal (95% CI)**		**687**		**719**	**100.0%**	**0.84 [0.33, 2.12]**
Total events	8		10			
Heterogeneity: Tau² = 0.00; Chi² = 0.10, df = 2 (P = 0.95); I² = 0%						
Test for overall effect: Z = 0.36 (P = 0.72)						
**Total (95% CI)**		**687**		**719**	**100.0%**	**0.84 [0.33, 2.12]**
Total events	8		10			
Heterogeneity: Tau² = 0.00; Chi² = 0.10, df = 2 (P = 0.95); I² = 0%						
Test for overall effect: Z = 0.36 (P = 0.72)						
Test for subgroup differences: Not applicable						

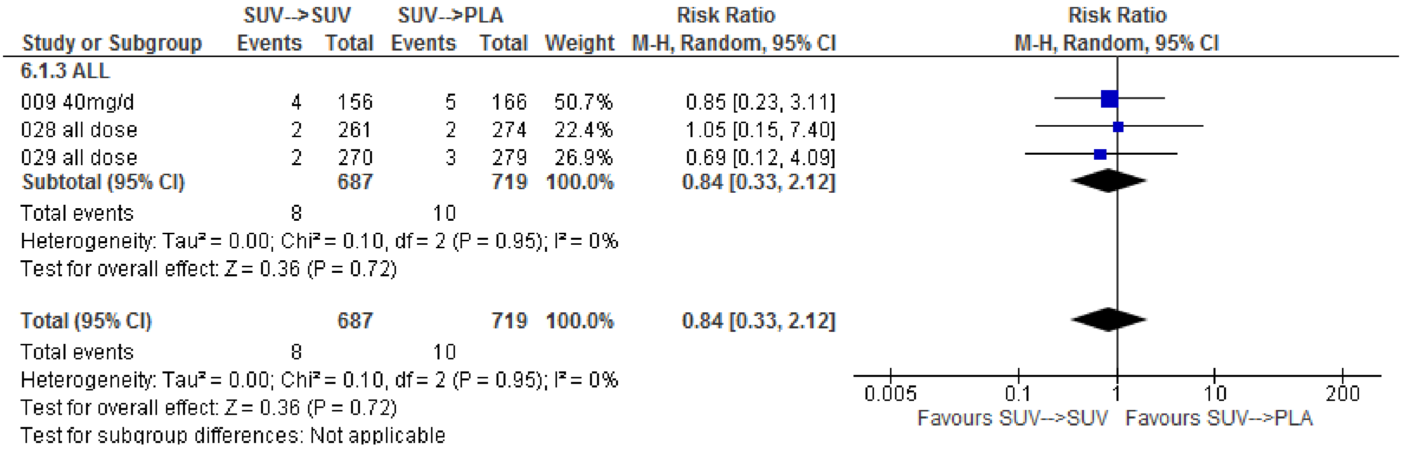

S3-2 Fig. Discontinuation due to intolerability

Study or Subgroup	SUV-->SUV Events	SUV-->SUV Total	SUV-->PLA Events	SUV-->PLA Total	Weight	Risk Ratio M-H, Random, 95% CI
**6.2.3 ALL**						
009 40mg/d	0	156	3	166	53.9%	0.15 [0.01, 2.92]
028 all dose	0	261	1	274	46.1%	0.35 [0.01, 8.55]
029 all dose	0	270	0	279		Not estimable
**Subtotal (95% CI)**		**687**		**719**	**100.0%**	**0.22 [0.03, 1.95]**
Total events	0		4			
Heterogeneity: Tau² = 0.00; Chi² = 0.14, df = 1 (P = 0.71); I² = 0%						
Test for overall effect: Z = 1.35 (P = 0.18)						
**Total (95% CI)**		**687**		**719**	**100.0%**	**0.22 [0.03, 1.95]**
Total events	0		4			
Heterogeneity: Tau² = 0.00; Chi² = 0.14, df = 1 (P = 0.71); I² = 0%						
Test for overall effect: Z = 1.35 (P = 0.18)						
Test for subgroup differences: Not applicable						

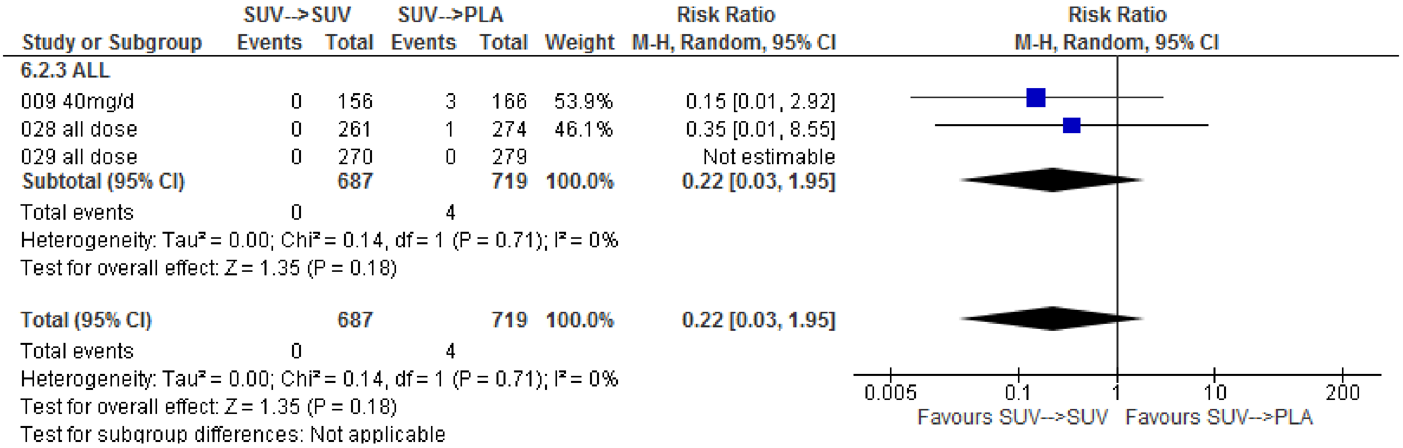

S3-3 Fig. Discontinuation due to inefficacy

Study or Subgroup	SUV-->SUV Events	SUV-->SUV Total	SUV-->PLA Events	SUV-->PLA Total	Weight	Risk Ratio M-H, Random, 95% CI
**6.3.3 ALL**						
009 40mg/d	0	156	1	166	100.0%	0.35 [0.01, 8.64]
028 all dose	0	261	0	274		Not estimable
029 all dose	0	270	0	279		Not estimable
**Subtotal (95% CI)**		**687**		**719**	**100.0%**	**0.35 [0.01, 8.64]**
Total events	0		1			
Heterogeneity: Not applicable						
Test for overall effect: Z = 0.64 (P = 0.52)						
**Total (95% CI)**		**687**		**719**	**100.0%**	**0.35 [0.01, 8.64]**
Total events	0		1			
Heterogeneity: Not applicable						
Test for overall effect: Z = 0.64 (P = 0.52)						
Test for subgroup differences: Not applicable						

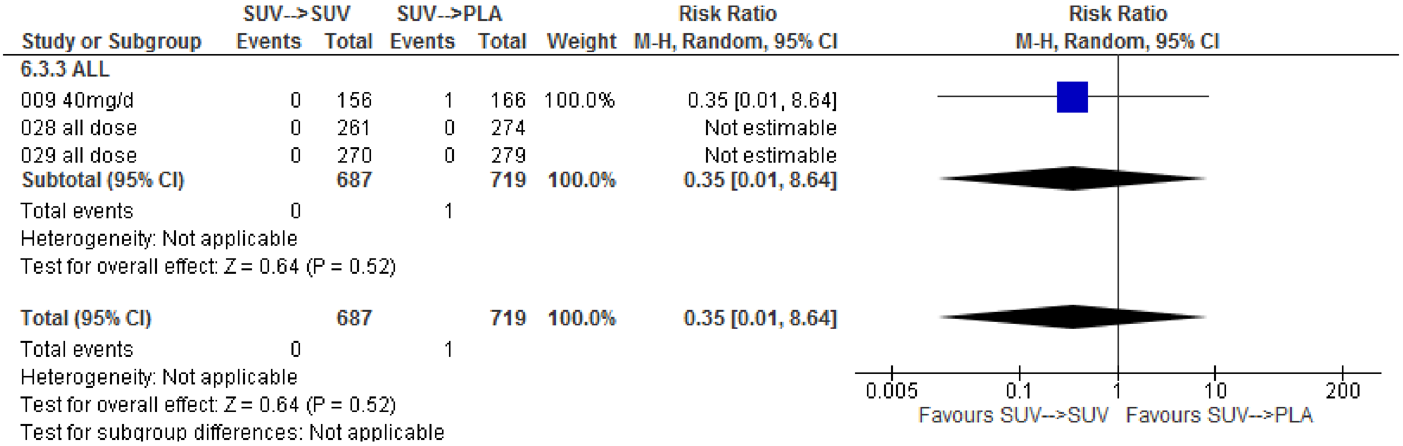

S3-4 Fig. Withdrawal (Tyrer Withdrawal Symptom Questionnaire) at Day 1

Study or Subgroup	SUV-->SUV Events	SUV-->SUV Total	SUV-->PLA Events	SUV-->PLA Total	Weight	Risk Ratio M-H, Random, 95% CI
**6.4.3 ALL**						
009 40mg/d	1	121	2	122	9.1%	0.50 [0.05, 5.49]
028 all dose	5	223	5	226	34.6%	1.01 [0.30, 3.45]
029 all dose	6	211	12	229	56.2%	0.54 [0.21, 1.42]
**Subtotal (95% CI)**		**555**		**577**	**100.0%**	**0.67 [0.33, 1.38]**
Total events	12		19			
Heterogeneity: Tau² = 0.00; Chi² = 0.68, df = 2 (P = 0.71); I² = 0%						
Test for overall effect: Z = 1.09 (P = 0.28)						
**Total (95% CI)**		**555**		**577**	**100.0%**	**0.67 [0.33, 1.38]**
Total events	12		19			
Heterogeneity: Tau² = 0.00; Chi² = 0.68, df = 2 (P = 0.71); I² = 0%						
Test for overall effect: Z = 1.09 (P = 0.28)						
Test for subgroup differences: Not applicable						

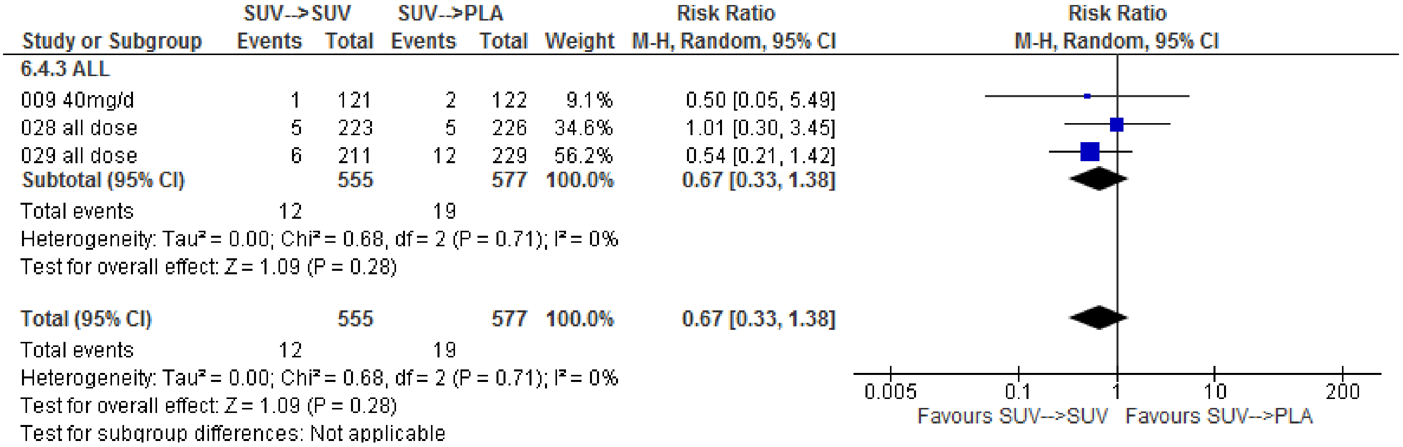

S3-5 Fig. Withdrawal (Tyrer Withdrawal Symptom Questionnaire) at Day 2

Study or Subgroup	SUV-->SUV Events	SUV-->SUV Total	SUV-->PLA Events	SUV-->PLA Total	Weight	Risk Ratio M-H, Random, 95% CI
**6.5.3 ALL**						
009 40mg/d	1	121	4	124	6.8%	0.26 [0.03, 2.26]
028 all dose	7	220	10	218	35.7%	0.69 [0.27, 1.79]
029 all dose	13	215	13	227	57.6%	1.06 [0.50, 2.23]
**Subtotal (95% CI)**		**556**		**569**	**100.0%**	**0.83 [0.47, 1.45]**
Total events	21		27			
Heterogeneity: Tau² = 0.00; Chi² = 1.67, df = 2 (P = 0.43); I² = 0%						
Test for overall effect: Z = 0.66 (P = 0.51)						
**Total (95% CI)**		**556**		**569**	**100.0%**	**0.83 [0.47, 1.45]**
Total events	21		27			
Heterogeneity: Tau² = 0.00; Chi² = 1.67, df = 2 (P = 0.43); I² = 0%						
Test for overall effect: Z = 0.66 (P = 0.51)						
Test for subgroup differences: Not applicable						

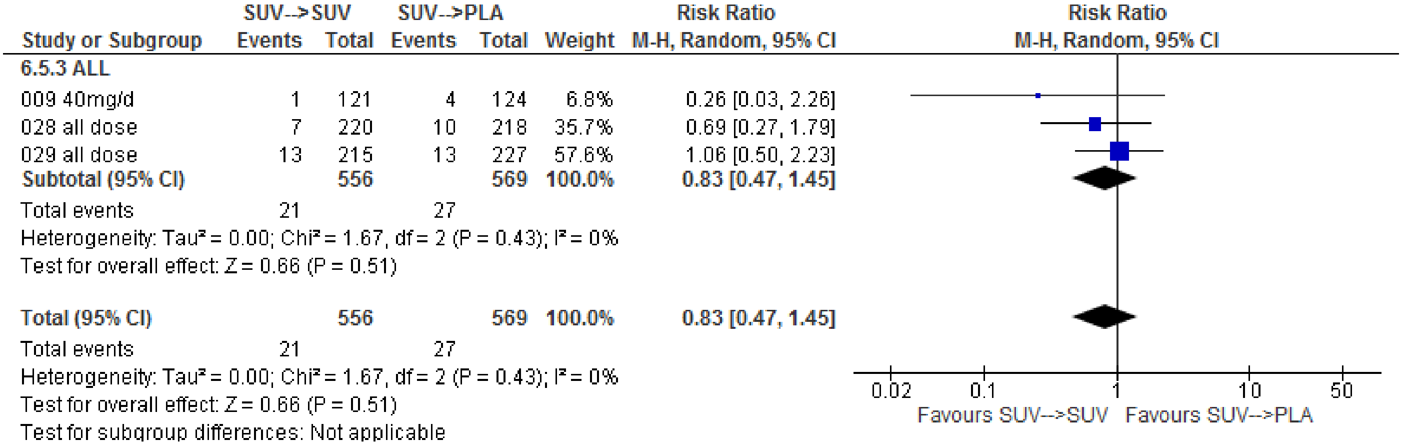

S3-6 Fig. Withdrawal (Tyrer Withdrawal Symptom Questionnaire) at Day 3

Study or Subgroup	SUV-->SUV Events	SUV-->SUV Total	SUV-->PLA Events	SUV-->PLA Total	Weight	Risk Ratio M-H, Random, 95% CI
**6.6.3 ALL**						
009 40mg/d	2	116	3	120	14.5%	0.69 [0.12, 4.05]
028 all dose	5	220	4	218	26.8%	1.24 [0.34, 4.55]
029 all dose	9	202	10	225	58.7%	1.00 [0.42, 2.42]
**Subtotal (95% CI)**		**538**		**563**	**100.0%**	**1.01 [0.51, 1.97]**
Total events	16		17			
Heterogeneity: Tau² = 0.00; Chi² = 0.27, df = 2 (P = 0.87); I² = 0%						
Test for overall effect: Z = 0.01 (P = 0.99)						
**Total (95% CI)**		**538**		**563**	**100.0%**	**1.01 [0.51, 1.97]**
Total events	16		17			
Heterogeneity: Tau² = 0.00; Chi² = 0.27, df = 2 (P = 0.87); I² = 0%						
Test for overall effect: Z = 0.01 (P = 0.99)						
Test for subgroup differences: Not applicable						

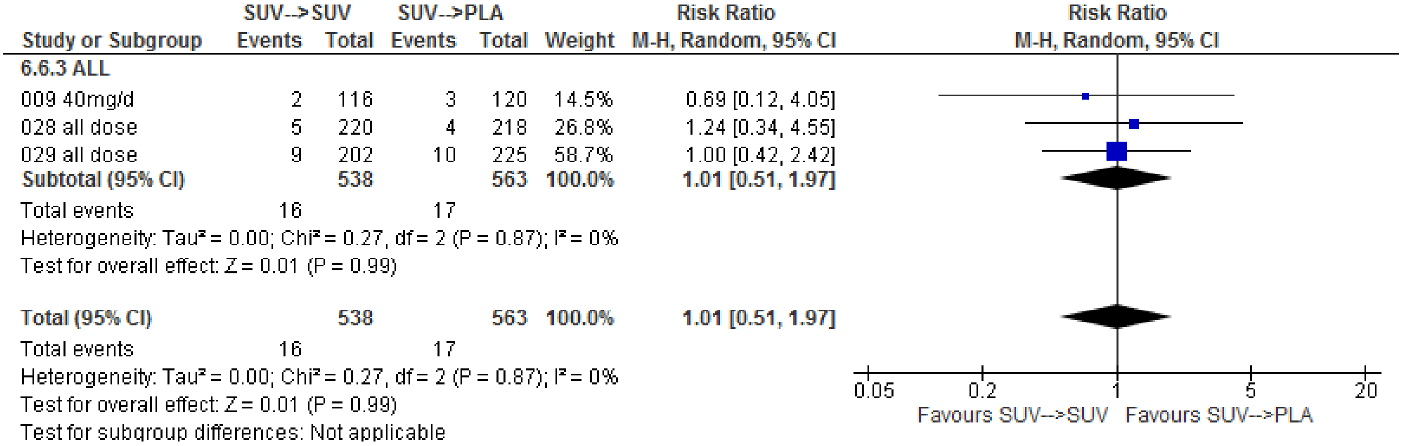

S3-7 Fig. Withdrawal (Tyrer Withdrawal Symptom Questionnaire) at Day 1-3

Study or Subgroup	SUV-->SUV Events	Total	SUV-->PLA Events	Total	Weight	Risk Ratio M-H, Random, 95% CI
**6.7.3 ALL**						
009 40mg/d	8	129	8	129	20.4%	1.00 [0.39, 2.58]
028 all dose	11	240	11	238	27.5%	0.99 [0.44, 2.24]
029 all dose	19	227	21	244	52.1%	0.97 [0.54, 1.76]
**Subtotal (95% CI)**		**596**		**611**	**100.0%**	**0.98 [0.64, 1.51]**
Total events	38		40			
Heterogeneity: Tau² = 0.00; Chi² = 0.00, df = 2 (P = 1.00); I² = 0%						
Test for overall effect: Z = 0.08 (P = 0.94)						
**Total (95% CI)**		**596**		**611**	**100.0%**	**0.98 [0.64, 1.51]**
Total events	38		40			
Heterogeneity: Tau² = 0.00; Chi² = 0.00, df = 2 (P = 1.00); I² = 0%						
Test for overall effect: Z = 0.08 (P = 0.94)						
Test for subgroup differences: Not applicable						

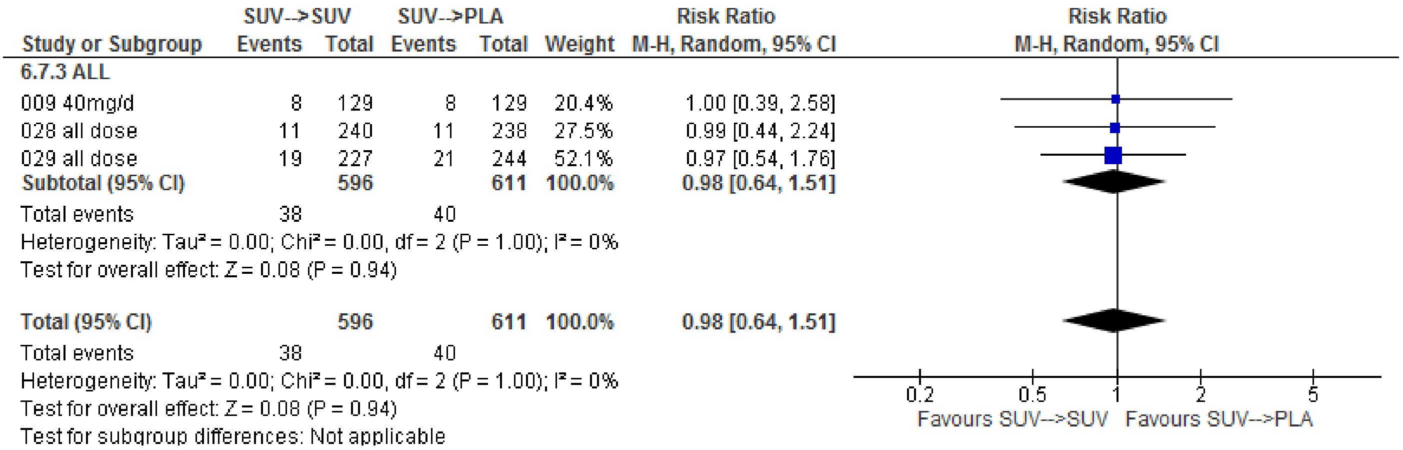

S3-8 Fig. Rebound insomnia (subjective total sleep time) at Day 1

Study or Subgroup	SUV-->PLA Events	Total	PLA-->PLA Events	Total	Weight	Risk Ratio M-H, Random, 95% CI
**6.8.3 ALL**						
009 40mg/d	48	142	40	139	21.6%	1.17 [0.83, 1.66]
028 all dose	86	256	83	298	41.5%	1.21 [0.94, 1.55]
029 all dose	78	256	78	307	36.9%	1.20 [0.92, 1.57]
**Subtotal (95% CI)**		**654**		**744**	**100.0%**	**1.20 [1.02, 1.41]**
Total events	212		201			
Heterogeneity: Tau² = 0.00; Chi² = 0.01, df = 2 (P = 0.99); I² = 0%						
Test for overall effect: Z = 2.18 (P = 0.03)						
**Total (95% CI)**		**654**		**744**	**100.0%**	**1.20 [1.02, 1.41]**
Total events	212		201			
Heterogeneity: Tau² = 0.00; Chi² = 0.01, df = 2 (P = 0.99); I² = 0%						
Test for overall effect: Z = 2.18 (P = 0.03)						
Test for subgroup differences: Not applicable						

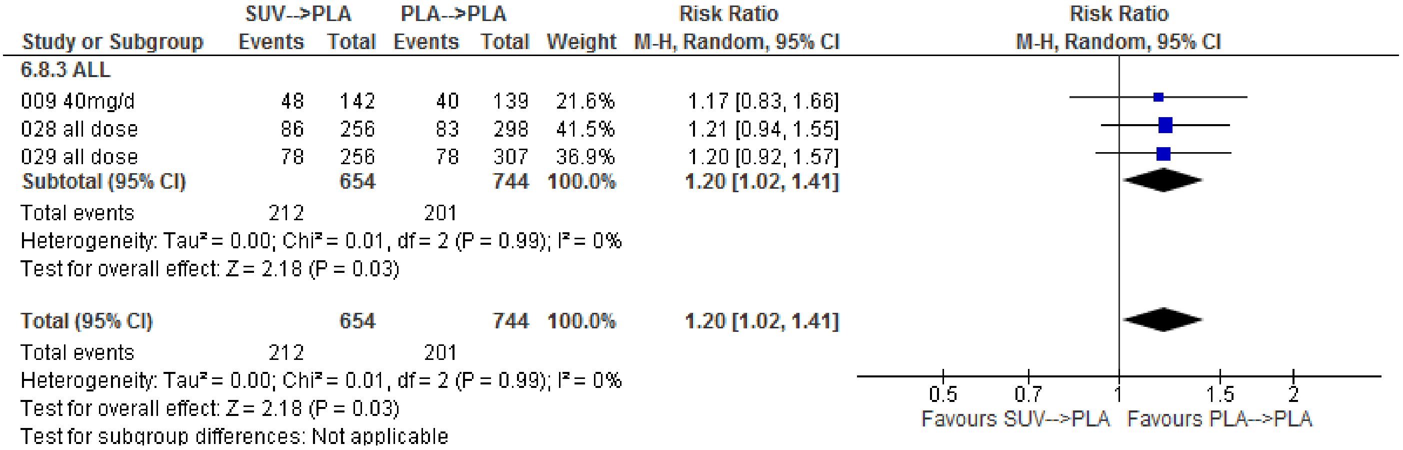

S3-9 Fig. Rebound insomnia (subjective total sleep time) at Day 2

Study or Subgroup	SUV-->PLA Events	Total	PLA-->PLA Events	Total	Weight	Risk Ratio M-H, Random, 95% CI
**6.9.3 ALL**						
009 40mg/d	52	146	39	145	29.2%	1.32 [0.94, 1.87]
028 all dose	61	243	74	302	40.2%	1.02 [0.76, 1.37]
029 all dose	58	255	51	308	30.6%	1.37 [0.98, 1.93]
**Subtotal (95% CI)**		**644**		**755**	**100.0%**	**1.21 [1.00, 1.46]**
Total events	171		164			
Heterogeneity: Tau² = 0.00; Chi² = 2.04, df = 2 (P = 0.36); I² = 2%						
Test for overall effect: Z = 1.97 (P = 0.05)						
**Total (95% CI)**		**644**		**755**	**100.0%**	**1.21 [1.00, 1.46]**
Total events	171		164			
Heterogeneity: Tau² = 0.00; Chi² = 2.04, df = 2 (P = 0.36); I² = 2%						
Test for overall effect: Z = 1.97 (P = 0.05)						
Test for subgroup differences: Not applicable						

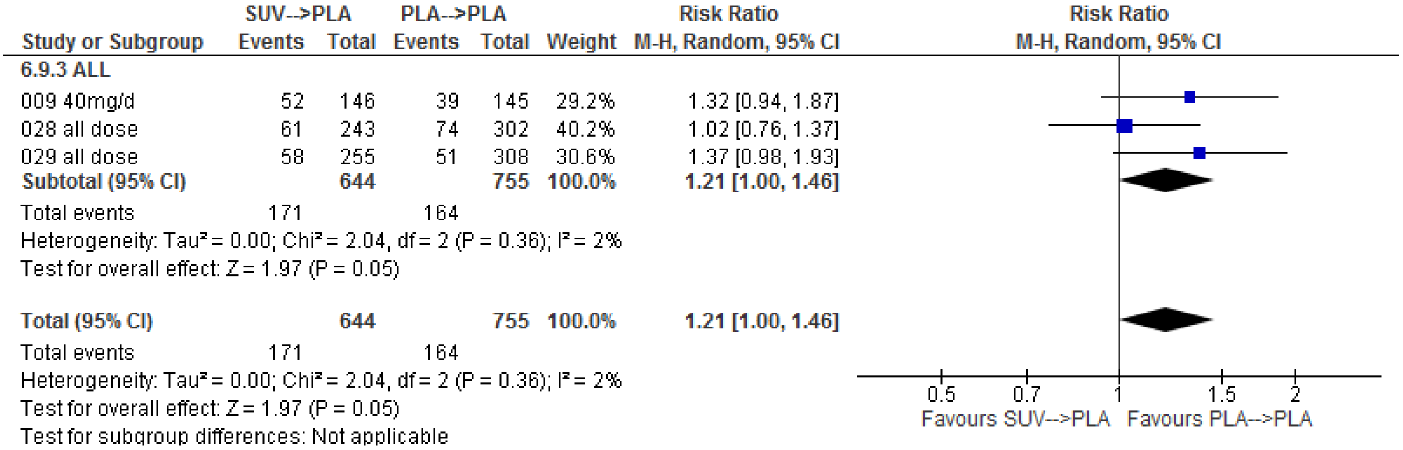

S3-10 Fig. Rebound insomnia (subjective total sleep time) at Day 3

Study or Subgroup	SUV-->PLA Events	SUV-->PLA Total	PLA-->PLA Events	PLA-->PLA Total	Weight	Risk Ratio M-H, Random, 95% CI
**6.10.3 ALL**						
009 40mg/d	55	146	44	139	31.1%	1.19 [0.86, 1.64]
028 all dose	62	253	64	294	34.3%	1.13 [0.83, 1.53]
029 all dose	72	255	57	300	34.6%	1.49 [1.10, 2.02]
**Subtotal (95% CI)**		**654**		**733**	**100.0%**	**1.26 [1.05, 1.51]**
Total events	189		165			
Heterogeneity: Tau² = 0.00; Chi² = 1.77, df = 2 (P = 0.41); I² = 0%						
Test for overall effect: Z = 2.53 (P = 0.01)						
**Total (95% CI)**		**654**		**733**	**100.0%**	**1.26 [1.05, 1.51]**
Total events	189		165			
Heterogeneity: Tau² = 0.00; Chi² = 1.77, df = 2 (P = 0.41); I² = 0%						
Test for overall effect: Z = 2.53 (P = 0.01)						
Test for subgroup differences: Not applicable						

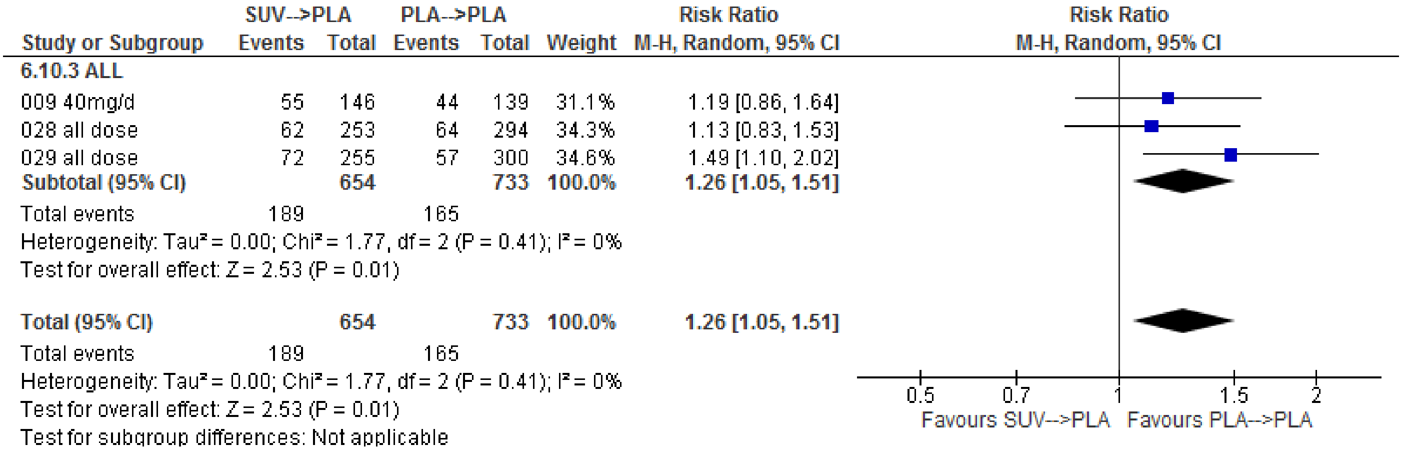

S3-11 Fig. Rebound insomnia (subjective total sleep time) at Day 1-3

Study or Subgroup	SUV-->PLA Events	SUV-->PLA Total	PLA-->PLA Events	PLA-->PLA Total	Weight	Risk Ratio M-H, Random, 95% CI
**6.11.3 ALL**						
009 40mg/d	80	157	61	152	23.9%	1.27 [0.99, 1.63]
028 all dose	116	265	124	319	39.0%	1.13 [0.93, 1.37]
029 all dose	125	271	110	322	37.1%	1.35 [1.11, 1.65]
**Subtotal (95% CI)**		**693**		**793**	**100.0%**	**1.24 [1.10, 1.40]**
Total events	321		295			
Heterogeneity: Tau² = 0.00; Chi² = 1.69, df = 2 (P = 0.43); I² = 0%						
Test for overall effect: Z = 3.48 (P = 0.0005)						
**Total (95% CI)**		**693**		**793**	**100.0%**	**1.24 [1.10, 1.40]**
Total events	321		295			
Heterogeneity: Tau² = 0.00; Chi² = 1.69, df = 2 (P = 0.43); I² = 0%						
Test for overall effect: Z = 3.48 (P = 0.0005)						
Test for subgroup differences: Not applicable						

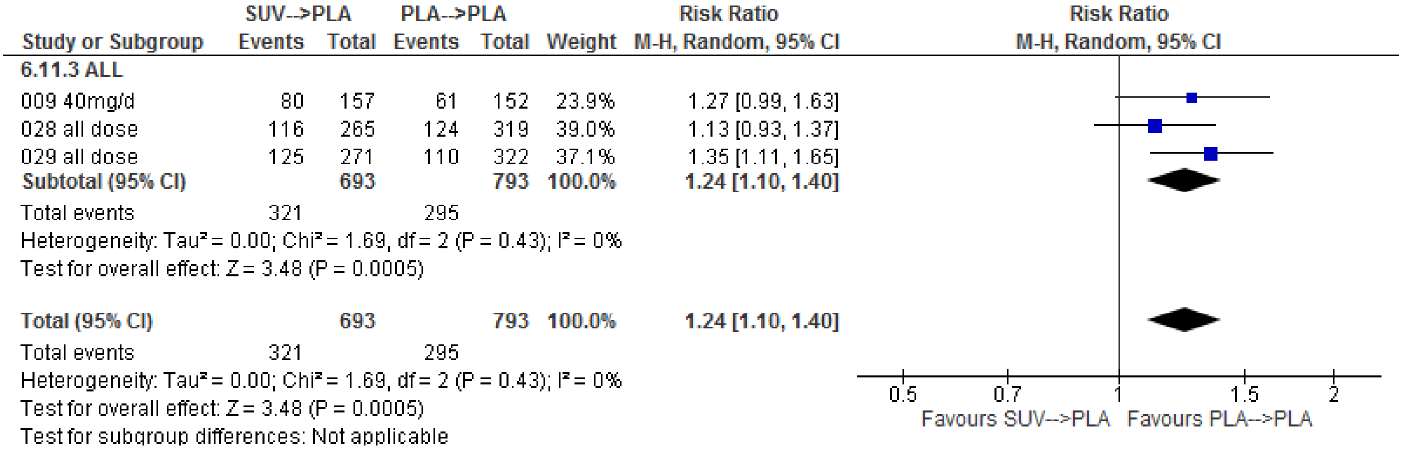

S3-12 Fig. Rebound insomnia (subjective time to sleep onset) at Day 1

Study or Subgroup	SUV-->PLA Events	SUV-->PLA Total	PLA-->PLA Events	PLA-->PLA Total	Weight	Risk Ratio M-H, Random, 95% CI
**6.12.3 ALL**						
009 40mg/d	38	142	31	139	20.3%	1.20 [0.79, 1.81]
028 all dose	71	256	68	298	41.9%	1.22 [0.91, 1.62]
029 all dose	57	256	74	307	37.8%	0.92 [0.68, 1.25]
**Subtotal (95% CI)**		**654**		**744**	**100.0%**	**1.09 [0.91, 1.32]**
Total events	166		173			
Heterogeneity: Tau² = 0.00; Chi² = 1.91, df = 2 (P = 0.39); I² = 0%						
Test for overall effect: Z = 0.94 (P = 0.35)						
**Total (95% CI)**		**654**		**744**	**100.0%**	**1.09 [0.91, 1.32]**
Total events	166		173			
Heterogeneity: Tau² = 0.00; Chi² = 1.91, df = 2 (P = 0.39); I² = 0%						
Test for overall effect: Z = 0.94 (P = 0.35)						
Test for subgroup differences: Not applicable						

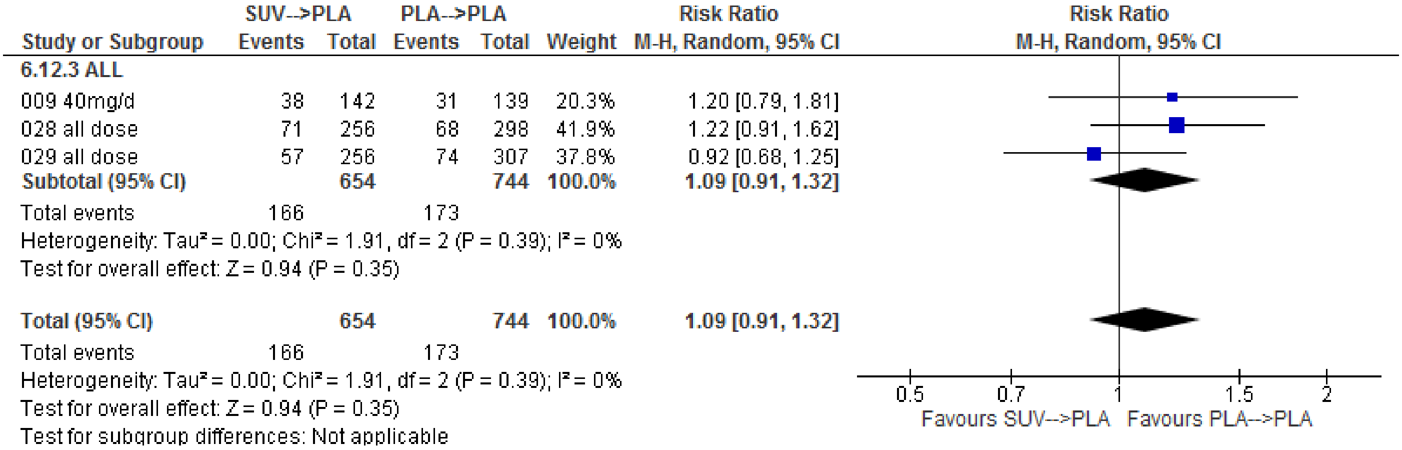

S3-13 Fig. Rebound insomnia (subjective time to sleep onset) at Day 2

	SUV-->PLA		PLA-->PLA			Risk Ratio
**Study or Subgroup**	**Events**	**Total**	**Events**	**Total**	**Weight**	**M-H, Random, 95% CI**
**6.13.3 ALL**						
009 40mg/d	44	146	36	145	31.1%	1.21 [0.83, 1.77]
028 all dose	48	243	56	302	36.6%	1.07 [0.75, 1.51]
029 all dose	41	255	55	308	32.3%	0.90 [0.62, 1.30]
**Subtotal (95% CI)**		**644**		**755**	**100.0%**	**1.05 [0.85, 1.30]**
Total events	133		147			
Heterogeneity: Tau² = 0.00; Chi² = 1.25, df = 2 (P = 0.54); I² = 0%						
Test for overall effect: Z = 0.46 (P = 0.64)						
**Total (95% CI)**		**644**		**755**	**100.0%**	**1.05 [0.85, 1.30]**
Total events	133		147			
Heterogeneity: Tau² = 0.00; Chi² = 1.25, df = 2 (P = 0.54); I² = 0%						
Test for overall effect: Z = 0.46 (P = 0.64)						
Test for subgroup differences: Not applicable						

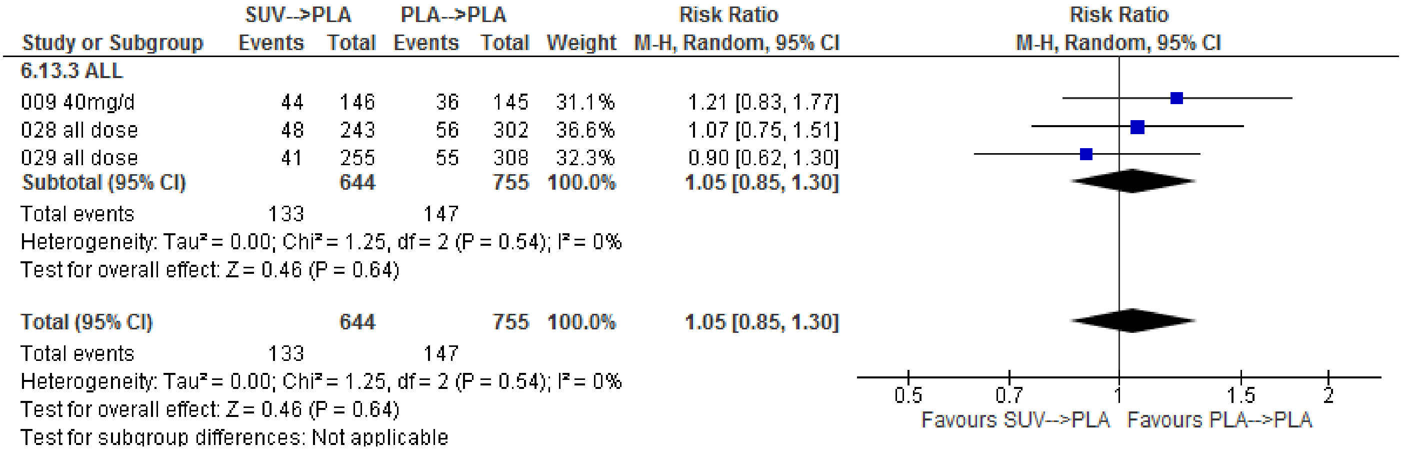

S3-14 Fig. Rebound insomnia (subjective time to sleep onset) at Day 3

	SUV-->PLA		PLA-->PLA			Risk Ratio
**Study or Subgroup**	**Events**	**Total**	**Events**	**Total**	**Weight**	**M-H, Random, 95% CI**
**6.14.3 ALL**						
009 40mg/d	44	146	35	139	28.3%	1.20 [0.82, 1.75]
028 all dose	58	253	56	294	35.3%	1.20 [0.87, 1.67]
029 all dose	51	255	70	300	36.4%	0.86 [0.62, 1.18]
**Subtotal (95% CI)**		**654**		**733**	**100.0%**	**1.06 [0.85, 1.33]**
Total events	153		161			
Heterogeneity: Tau² = 0.01; Chi² = 2.67, df = 2 (P = 0.26); I² = 25%						
Test for overall effect: Z = 0.52 (P = 0.60)						
**Total (95% CI)**		**654**		**733**	**100.0%**	**1.06 [0.85, 1.33]**
Total events	153		161			
Heterogeneity: Tau² = 0.01; Chi² = 2.67, df = 2 (P = 0.26); I² = 25%						
Test for overall effect: Z = 0.52 (P = 0.60)						
Test for subgroup differences: Not applicable						

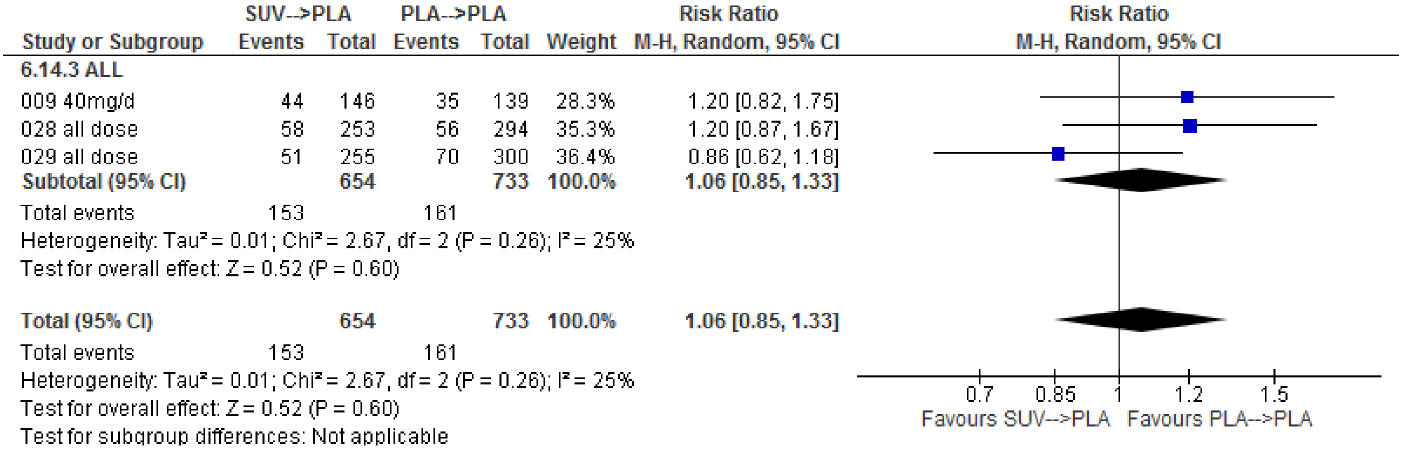

S3-15 Fig. Rebound insomnia (subjective time to sleep onset) at Day 1-3

	SUV-->PLA		PLA-->PLA			Risk Ratio
**Study or Subgroup**	**Events**	**Total**	**Events**	**Total**	**Weight**	**M-H, Random, 95% CI**
**6.15.3 ALL**						
009 40mg/d	64	157	55	152	24.5%	1.13 [0.85, 1.50]
028 all dose	102	265	101	319	37.8%	1.22 [0.97, 1.52]
029 all dose	92	271	114	322	37.7%	0.96 [0.77, 1.20]
**Subtotal (95% CI)**		**693**		**793**	**100.0%**	**1.09 [0.94, 1.26]**
Total events	258		270			
Heterogeneity: Tau² = 0.00; Chi² = 2.26, df = 2 (P = 0.32); I² = 12%						
Test for overall effect: Z = 1.17 (P = 0.24)						
**Total (95% CI)**		**693**		**793**	**100.0%**	**1.09 [0.94, 1.26]**
Total events	258		270			
Heterogeneity: Tau² = 0.00; Chi² = 2.26, df = 2 (P = 0.32); I² = 12%						
Test for overall effect: Z = 1.17 (P = 0.24)						
Test for subgroup differences: Not applicable						

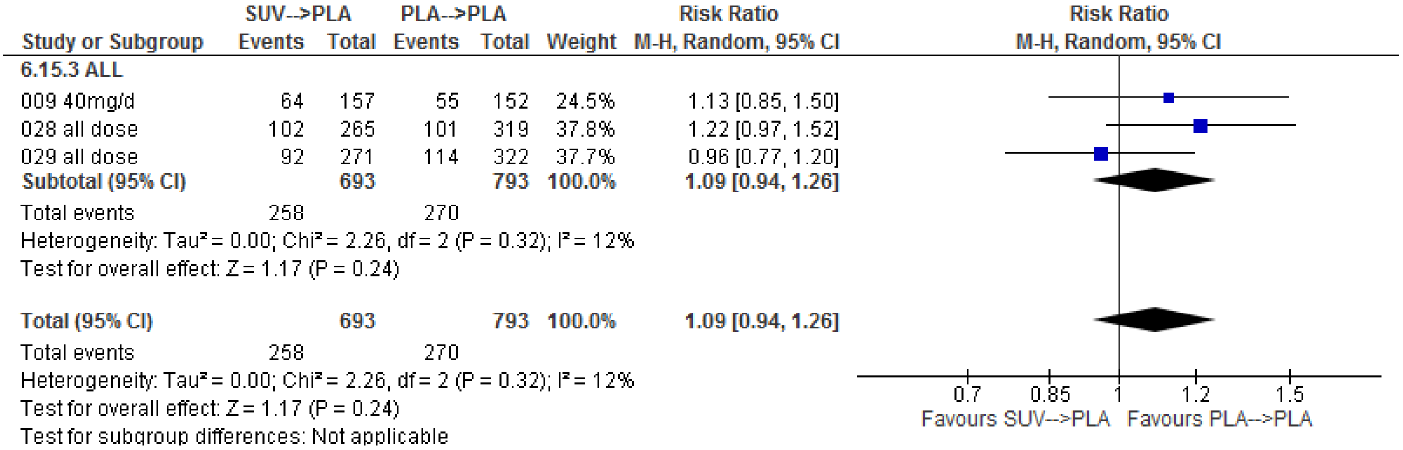

Supplement: S3 Fig — (PDF) [file pone.0136910.s004.pdf]
