# Supplementary material for: Suvorexant for Primary Insomnia: A Systematic Review and Meta-Analysis of Randomized Placebo-Controlled Trials
Source: PLoS One. 2015 Aug 28;10(8):e0136910. doi: 10.1371/journal.pone.0136910 (PMC4552781; doi:10.1371/journal.pone.0136910)
Supplement: S1 Table — (PDF) [file pone.0136910.s005.pdf]

	006 study	009 study	028 study	029 study
Subjective total sleep time, Week 1		diary (change scores)	diary (change scores)	diary (change scores)
Subjective total sleep time, Month 1*		diary (change scores)	diary (change scores)	diary (change scores)
Subjective total sleep time, Month 3			diary (change scores)	diary (change scores)
Subjective time-to-sleep onset, Week 1		diary (change scores)	diary (change scores)	diary (change scores)
Subjective time-to-sleep onset, Month 1*		diary (change scores)	diary (change scores)	diary (change scores)
Subjective time-to-sleep onset, Month 3			diary (change scores)	diary (change scores)
Subjective wake after sleep onset, Week 1			diary (change scores)	diary (change scores)
Subjective wake after sleep onset, Month 1		diary (change scores)	diary (change scores)	diary (change scores)
Subjective wake after sleep onset, Month 3			diary (change scores)	diary (change scores)
Latency to persistent sleep, Day 1	PSG (endpoint scores, period 1)		PSG (change scores)	PSG (change scores)
Latency to persistent sleep, Month 1	PSG (endpoint scores, period 1)		PSG (change scores)	PSG (change scores)
Latency to persistent sleep, Month 3			PSG (change scores)	PSG (change scores)
Wake after sleep onset, Day 1			PSG (change scores)	PSG (change scores)
Wake after sleep onset, Month 1			PSG (change scores)	PSG (change scores)
Wake after sleep onset, Month 3			PSG (change scores)	PSG (change scores)
Subjective quality of sleep, Week 1			diary (change scores)	diary (change scores)
Subjective quality of sleep, Month 1		diary (change scores)	diary (change scores)	diary (change scores)
Subjective quality of sleep, Month 3			diary (change scores)	diary (change scores)
Subjective refreshed feeling on waking, Month 1		diary (change scores)	diary (change scores)	diary (change scores)
Subjective refreshed feeling on waking, Month 3			diary (change scores)	diary (change scores)
Subjective number of awakenings, Week 1			diary (change scores)	diary (change scores)
Subjective number of awakenings, Month 1		diary (change scores)	diary (change scores)	diary (change scores)
Subjective number of awakenings, Month 3			diary (change scores)	diary (change scores)
Insomnia Severity Index, Month 1			rating scale (change scores)	rating scale (change scores)
Insomnia Severity Index, Month 3			rating scale (change scores)	rating scale (change scores)

	006 study	009 study	028 study	029 study
Clinician global impression of severity, Week 2			rating scale (change scores)	rating scale (change scores)
Clinician global impression of severity, Month 1		rating scale (change scores)	rating scale (change scores)	rating scale (change scores)
Clinician global impression of severity, Month 3			rating scale (change scores)	rating scale (change scores)
Patient global impression of severity, Week 2			rating scale (change scores)	rating scale (change scores)
Patient global impression of severity, Month 1		rating scale (change scores)	rating scale (change scores)	rating scale (change scores)
Patient global impression of severity, Month 3			rating scale (change scores)	rating scale (change scores)
Clinician global impression of improvement, Week 2			rating scale (change scores)	rating scale (change scores)
Clinician global impression of improvement, Month 1		rating scale (change scores)	rating scale (change scores)	rating scale (change scores)
Clinician global impression of improvement, Month 3			rating scale (change scores)	rating scale (change scores)
Patient global impression of improvement, Week 2			rating scale (change scores)	rating scale (change scores)
Patient global impression of improvement, Month 1		rating scale (change scores)	rating scale (change scores)	rating scale (change scores)
Patient global impression of improvement, Month 3			rating scale (change scores)	rating scale (change scores)

S1 Table. Data synthesis.

PSG: polysomnography

* The primary outcomes for measuring efficacy were either subjective total sleep time or subjective time-to-sleep onset at 1 month.
