# Supplementary material for: Suvorexant for Primary Insomnia: A Systematic Review and Meta-Analysis of Randomized Placebo-Controlled Trials
Source: PLoS One. 2015 Aug 28;10(8):e0136910. doi: 10.1371/journal.pone.0136910 (PMC4552781; doi:10.1371/journal.pone.0136910)
Supplement: S2 Table — (PDF) [file pone.0136910.s006.pdf]

Study	Total n	Patients	Duration	Age (mean+/-SD)	Male, %	White (%)	Drug	n	Outcomes[a]
006 (2012). USA and Japan. Industry. NCT00792298	254	Primary insomnia (DSM-IV-TR). age: 18 to 64 years. 1-week, SB, PLA run-in period (PSG assessments: the first night and 1 week later. LPS>20 mins. on both PSG nights and mean WASO≥60 mins on both nights with neither night<45 min)	4 weeks	SUV10: 45.1+/-11.5,<br>SUV20: 44.3+/-11.1,<br>SUV40: 44.5+/-11.3,<br>SUV80: 43.8+/-12.1,<br>PLA: 44.3+/-11.5	SUV10: 45.2,<br>SUV20: 34.4,<br>SUV40: 45.8,<br>SUV80: 44.3,<br>PLA: 41.0	SUV10: 71.0<br>SUV20: 63.9,<br>SUV40: 71.2,<br>SUV80: 73.8,<br>PLA: 70.3	SUV10 mg/d (period 1/2)<br>SUV20 mg/d (period 1/2)<br>SUV40 mg/d (period 1/2)<br>SUV80 mg/d (period 1/2)<br>PLA (period 1/2)	63 (31/32)<br>65 (33/32)<br>64 (32/32/)<br>62 (31/31)<br>253 (127/126)	all dose>PLA: SE, WASO and TST, only SUV20, 40 and 80>PLA: ISI, only SUV40 and 80>PLA: sTST, sTSO, sQUAL, only SUV20>PLA: LPS, all dose=PLA: NAW and sFRESH
009 (2014). USA, Australia, Europe, and South Africa. Industry. NCT01021813	781	Primary insomnia (DSM-IV-TR, assessed by a clinical interview and a structured sleep diagnostic interview). age≥18 years.	52 weeks	SUV40: 61.3+/-14.5,<br>PLA: 62.0+/-14.6<br><65 years<br>SUV40: 213 (41%)<br>PLA: 107 (42%)<br>≥65 years<br>SUV40: 308 (59%)<br>PLA: 151 (59%)	SUV40: 44.9,<br>PLA: 42.3	SUV40: 91.4,<br>PLA:89.5	SUV40 mg/d<br>PLA	522<br>258	SUV40>PLA: sTST, sTSO, sWASO, sNAW, sQUAL, sFRESH, ISI, CGI-S, PGI-S, CGI-I and PGI-I
028 (2014). USA, Europe, Asia, and South Africa. Industry. NCT01097616	1022	Primary insomnia (DSM-IV-TR). age≥18 years. 2-weeks, SB, PLA run-in period (Q-cohort: sTST<6.5 hrs and sTSO>30 mins on ≥ 4 of 7 nights, PQ-cohort: LPS>20 mins and WASO≥60 min on combined nights with neither night≤45 mins.	3 months	SUV20: 55+/-16,<br>SUV40: 56+/-15,<br>PLA: 56+/-15<br><65 years<br>SUV20: 147 (57.9%)<br>SUV40: 222 (58.0%)<br>PLA: 223 (58.1%)<br>≥65 years<br>SUV20: 107 (42.1%)	SUV20: 36.2<br>SUV40: 39.9<br>PLA: 36.2	SUV20: 66.1<br>SUV40: 66.1<br>PLA: 63.5	SUV20 mg/d<br>SUV40 mg/d<br>PLA	254<br>383<br>385	all dose>PLA: sTST, sTSO, LPS, WASO, ISI, CGI-S, PGI-S, CGI-I and PGI-I, only SUV40>PLA: sWASO and sQUAL, all dose=PLA: sNAW and sFRESH

				SUV40: 161 (42.0%)<br>PLA: 161 (41.9%)					
Study	Total n	Patients	Duration	Age (mean+/-SD)	Male, %	White (%)	Drug	n	Outcomes[a]
029 (2014). USA, Australia, Europe, Asia, and South Africa.<br>Industry.<br>NCT01097629	1019	Primary insomnia (DSM-IV-TR). age≥18 years. 2-weeks, SB, PLA run-in period (Q-cohort: sTST<6.5 hrs and sTSO>30 mins on ≥ 4 of 7 nights, PQ-cohort: LPS>20 mins and WASO≥60 min on combined nights with neither night≤45 mins.	3 months	SUV20: 56+/-16,<br>SUV20: 57+/-15,<br>PLA: 57+/-15<br><65 years<br>SUV20: 144 (60.3%)<br>SUV40: 229 (59.2%)<br>PLA: 226 (59.0%)<br>≥65 years<br>SUV20: 95 (39.7%)<br>SUV40: 158 (40.8%)<br>PLA: 157 (41.0%)	SUV20: 34.3<br>SUV40: 31.0<br>PLA: 35.5	SUV20: 79.5<br>SUV40: 80.1<br>PLA: 80.7	SUV20 mg/d<br>SUV40 mg/d<br>PLA	240<br>392<br>387	all dose>PLA: sTST, sTSO, sWASO, sQUAL, sFRESH, WASO, ISI, CGI-S, PGI-S, CGI-I and PGI-I, all dose=PLA: LPS and sNAW

S2 Table. Study, patient, and treatment characteristics for the analyzed randomized, controlled trials.

a The primary outcome of 009 study was the safety and tolerability of SUV.

Q-cohort: Patients received only subjective assessment, PQ-cohort: Patients received subjective and objective assessment.

CGI-I: Clinical Global Impression-Improvement scale, CGI-S: Clinical Global Impression-Severity scale, d: day, DSM-IV-TR:, hr: hour, ISI: Insomnia Severity Index, LPS: latency to onset of persistent sleep, min: minute, sNAW: (subjective) number of wakenings, PGI-I: Patient Global Impression-Improvement scale, PGI-S: Patient Global Impression-Severity scale, PLA: placebo, PSG: polysomnography, SB: single-blind, SE: sleep efficiency, sFRESH: subjective refreshed feeling on waking, sQUAL: subjective quality of sleep, TSO: subjective time to sleep onset, (s)TST: (subjective) total sleep time, SUV: suvorexant, (s)WASO: (subjective) wakefulness after persistent sleep onset,
